# Supplementary material for: Reaction of Pyrrolobenzothiazines with Schiff Bases and Carbodiimides: Approach to Angular 6/5/5/5-Tetracyclic Spiroheterocycles
Source: Molecules. 2024 May 1;29(9):2089. doi: 10.3390/molecules29092089 (PMC11085407; doi:10.3390/molecules29092089)
Supplement: Supplementary file 1 [file molecules-29-02089-s001.zip › SI3.pdf]

# Supporting information

## For

# Reaction of Pyrrolobenzothiazines with Schiff Bases and Carbodiimides: Approach to Angular 6/5/5/5-Tetracyclic Spiroheterocycles


Ekaterina A. Lystsova [1], Anastasia D. Novokshonova [2,3], Pavel V. Khramtsov [2,3], Alexander S. Novikov [4,5], Maksim V. Dmitriev [1],

Andrey N. Maslivets [1] and Ekaterina E. Khramtsova [1,*]

[1] Department of Chemistry, Perm State University, ul. Bukireva, 15, 614990 Perm, Russia, caterina.stepanova@psu.ru

[2] Department of Biology, Perm State University, ul. Bukireva, 15, 614990 Perm, Russia

[3] Institute of Ecology and Genetics of Microorganisms, Perm Federal Research Center, The Ural Branch of Russian Academy of Sciences, ul. Goleva, 13, 614081 Perm, Russia

[4] Institute of Chemistry, Saint Petersburg State University, Universitetskaya nab. 7/9, 199034 St. Petersburg, Russia

[5] Research Institute of Chemistry, Peoples' Friendship University of Russia (RUDN University), ul. Miklukho-Maklaya, 6, 117198 Moscow, Russia

***** Correspondence: caterina.stepanova@psu.ru

## Table of contents

NMR charts of compounds **3a-m, 3'k,j, 8a-j, 4a,b, 6d** ……………………………………………………………..…..….S3
Calculated total electronic energies (E, in Hartree), enthalpies (H, in Hartree),
Gibbs free energies (G, in Hartree), and entropies (S, cal/mol•K) for optimized equilibrium model structures….….S86
ORTEP images of X-ray crystal structures of compounds **3a, 4b, 6d, 8a**….…………………………………..……..…S87

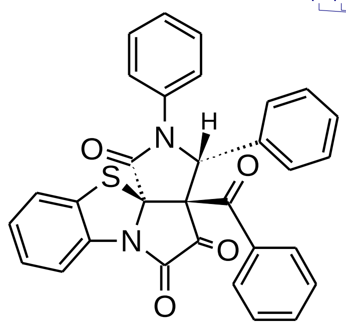

$^1$H NMR of **3a** (400 MHz, DMSO-$d_6$)

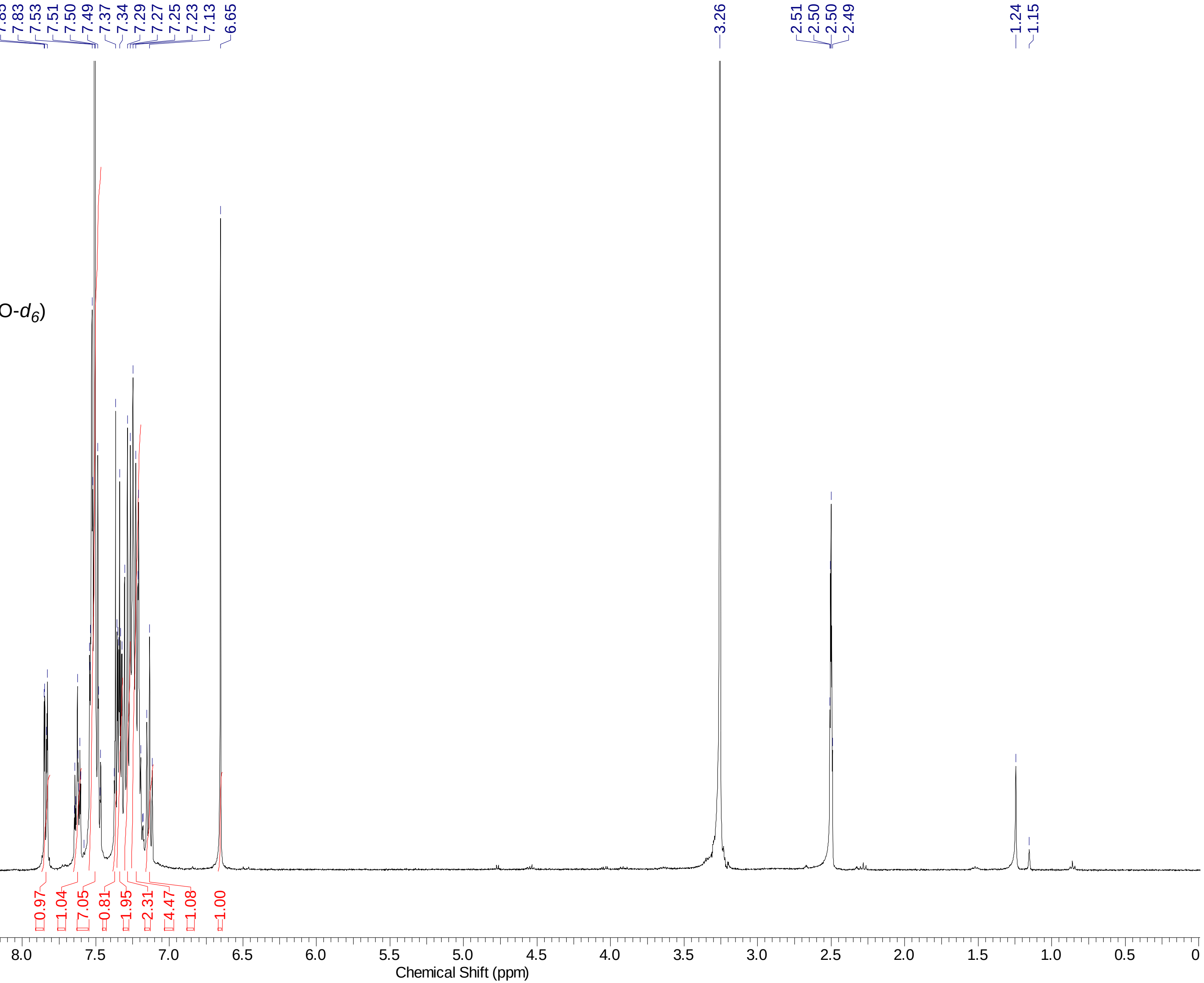

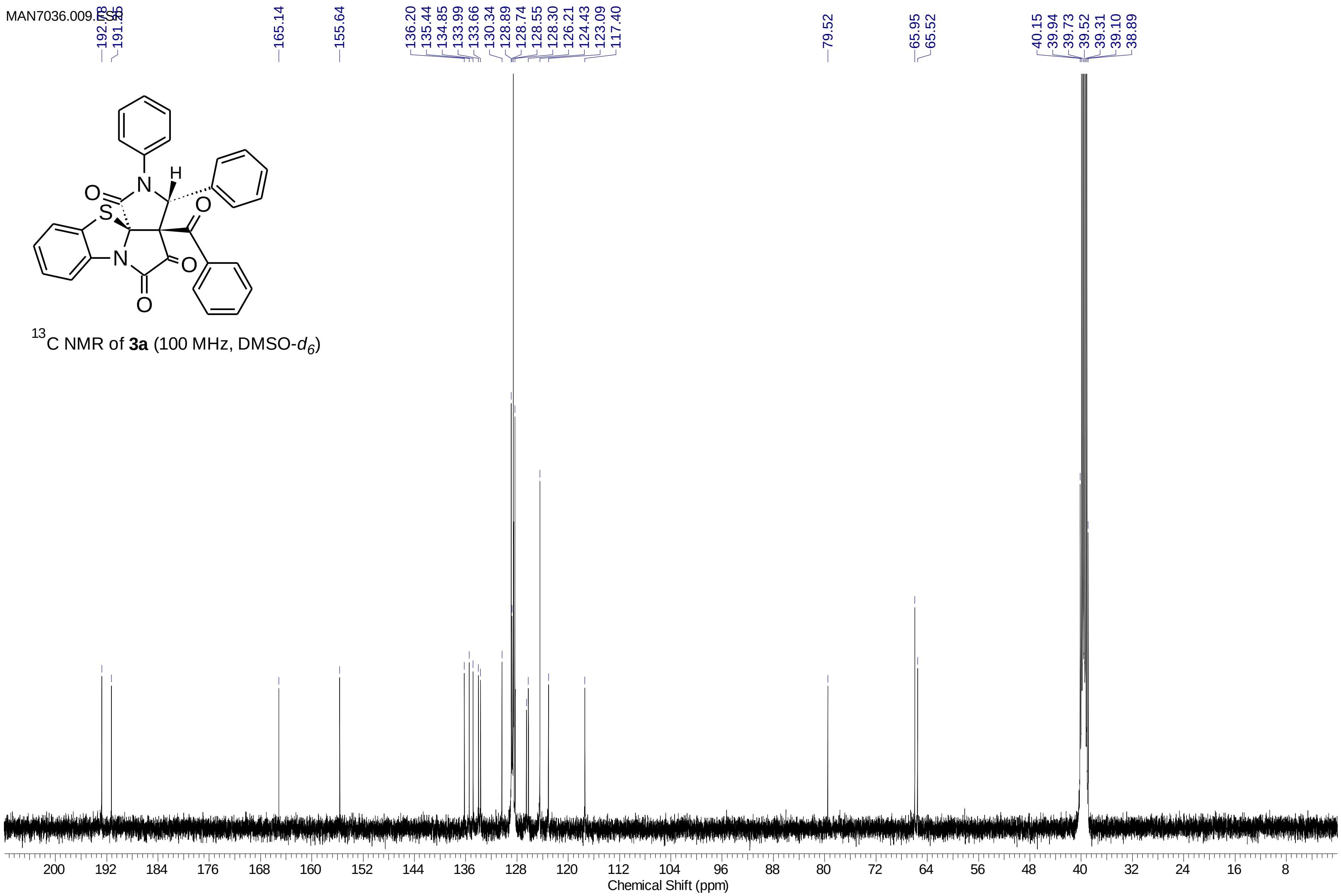


$^{13}$C NMR of **3a** (100 MHz, DMSO-$d_6$)

MAN7036.010.ESP

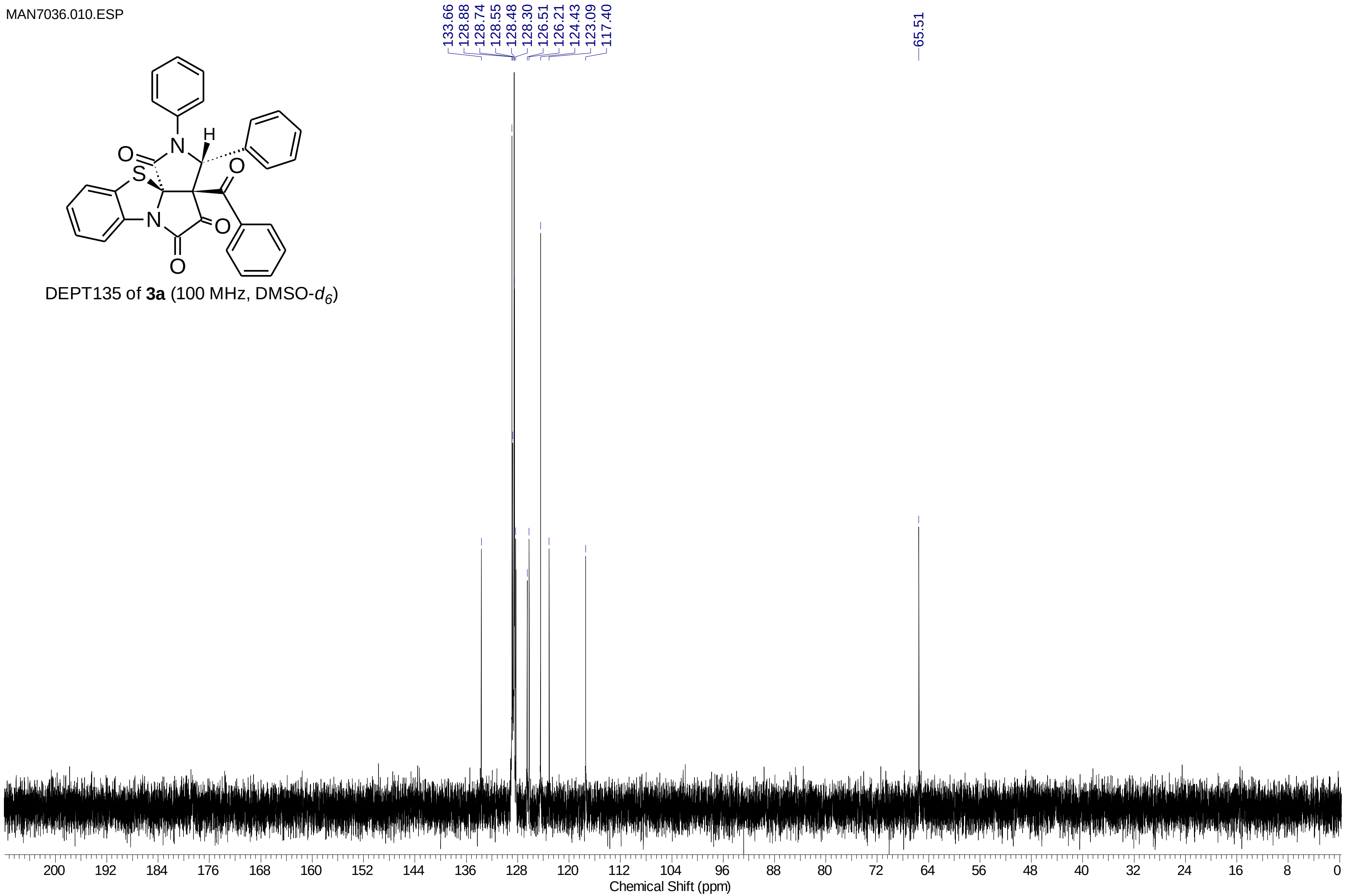


DEPT135 of **3a** (100 MHz, DMSO-$d_6$)

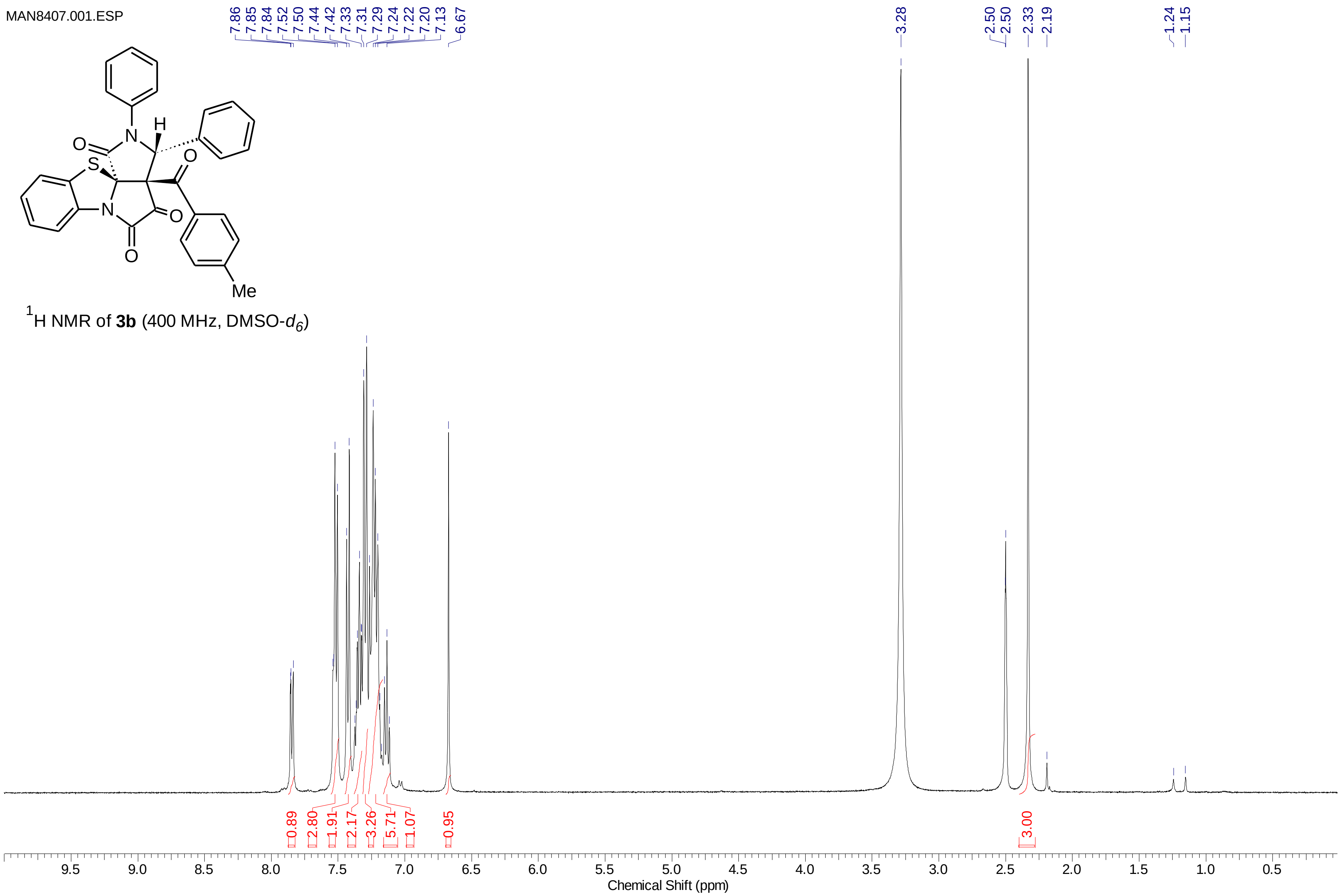


$^{1}$H NMR of **3b** (400 MHz, DMSO-$d_6$)

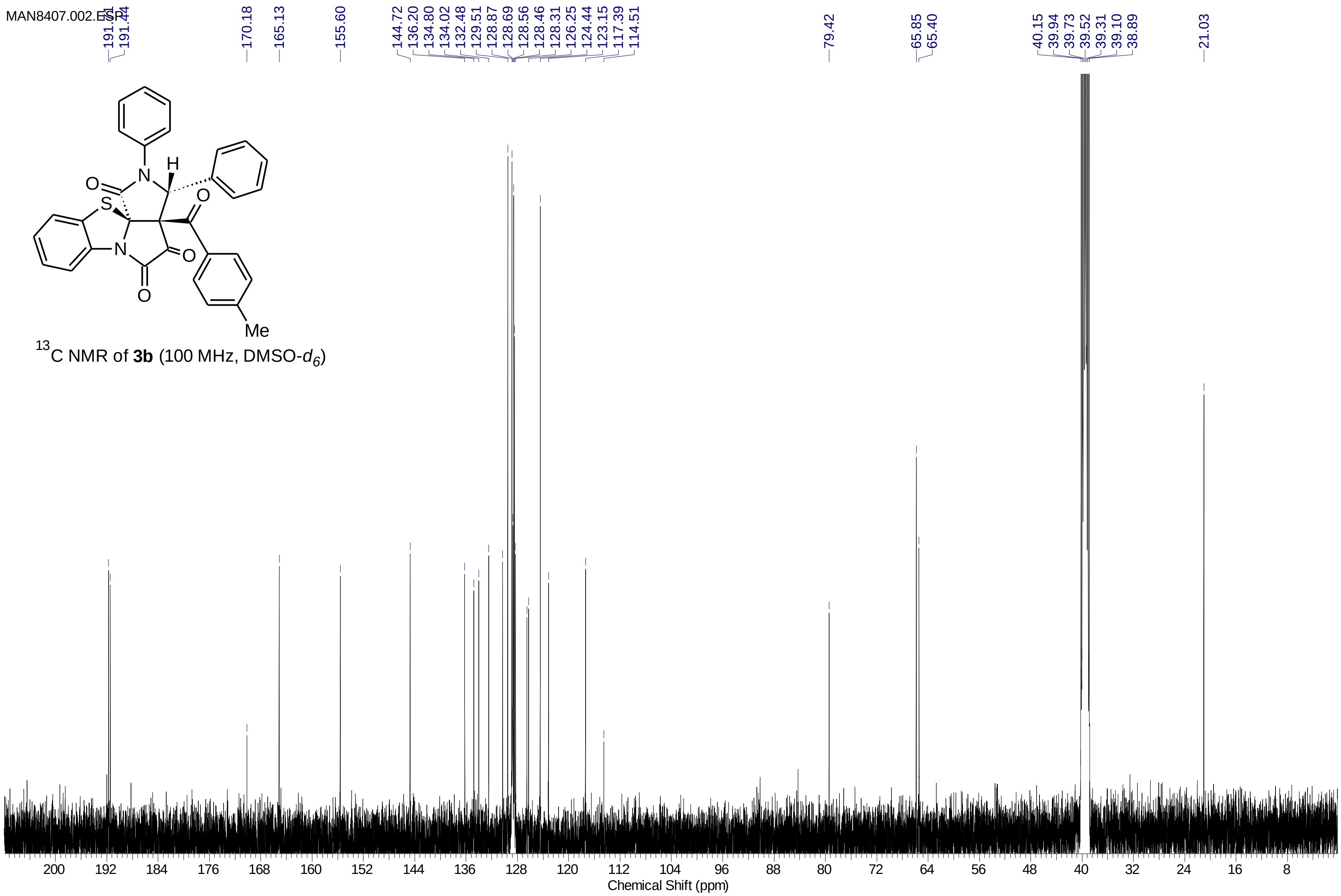


$^{13}$C NMR of **3b** (100 MHz, DMSO-$d_6$)

MAN8407.003.ESP

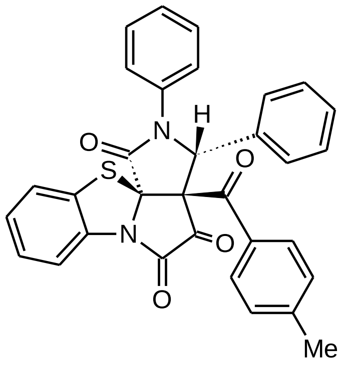


DEPT135 of **3b** (100 MHz, DMSO-$d_6$)

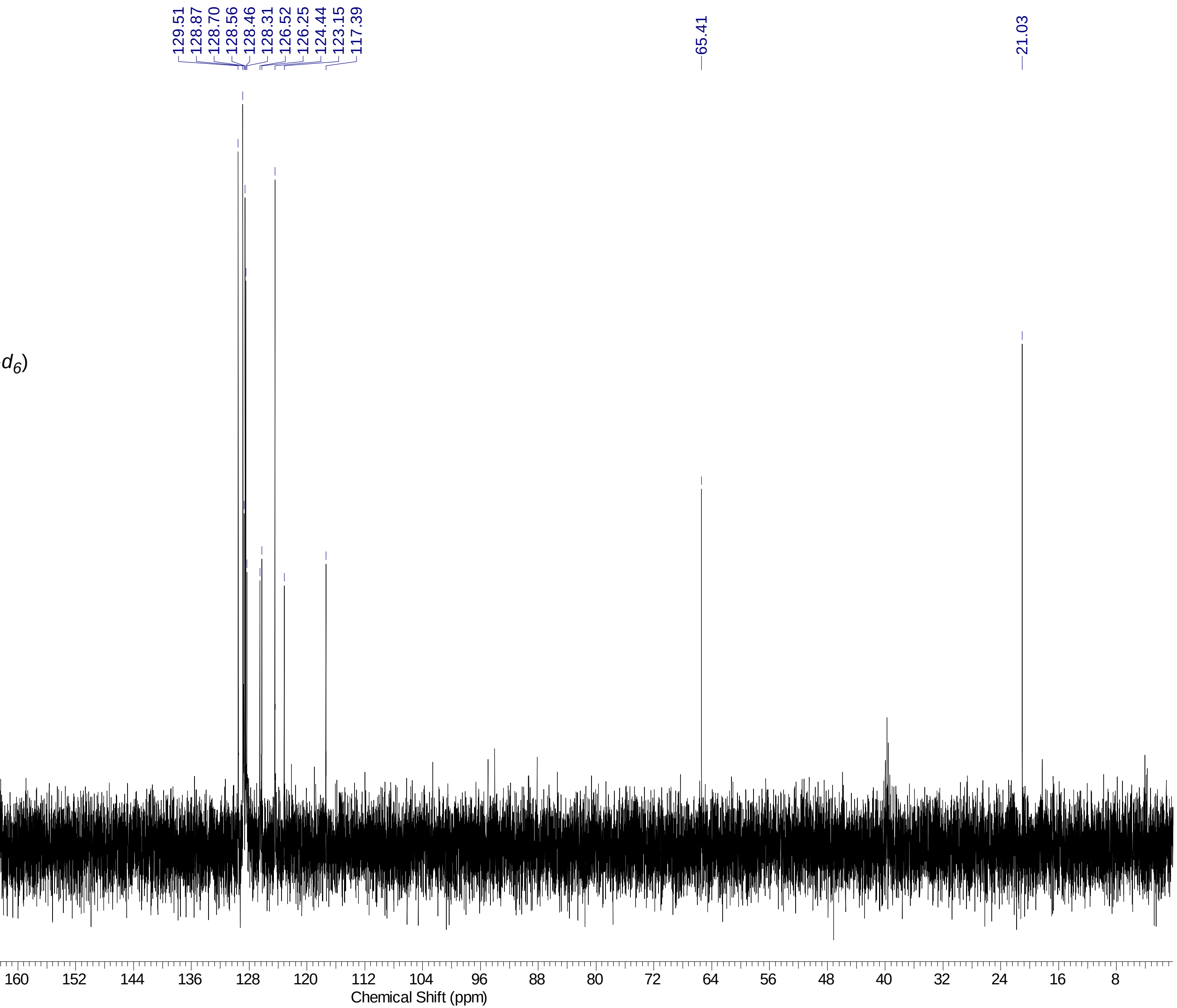

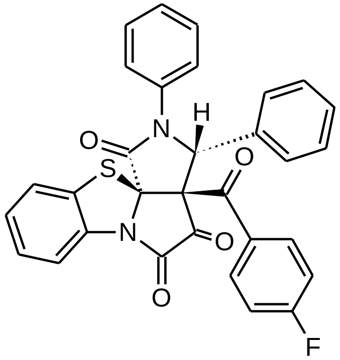


$^{1}$H NMR of **3c** (400 MHz, DMSO-$d_6$)
(solvate with toluene)

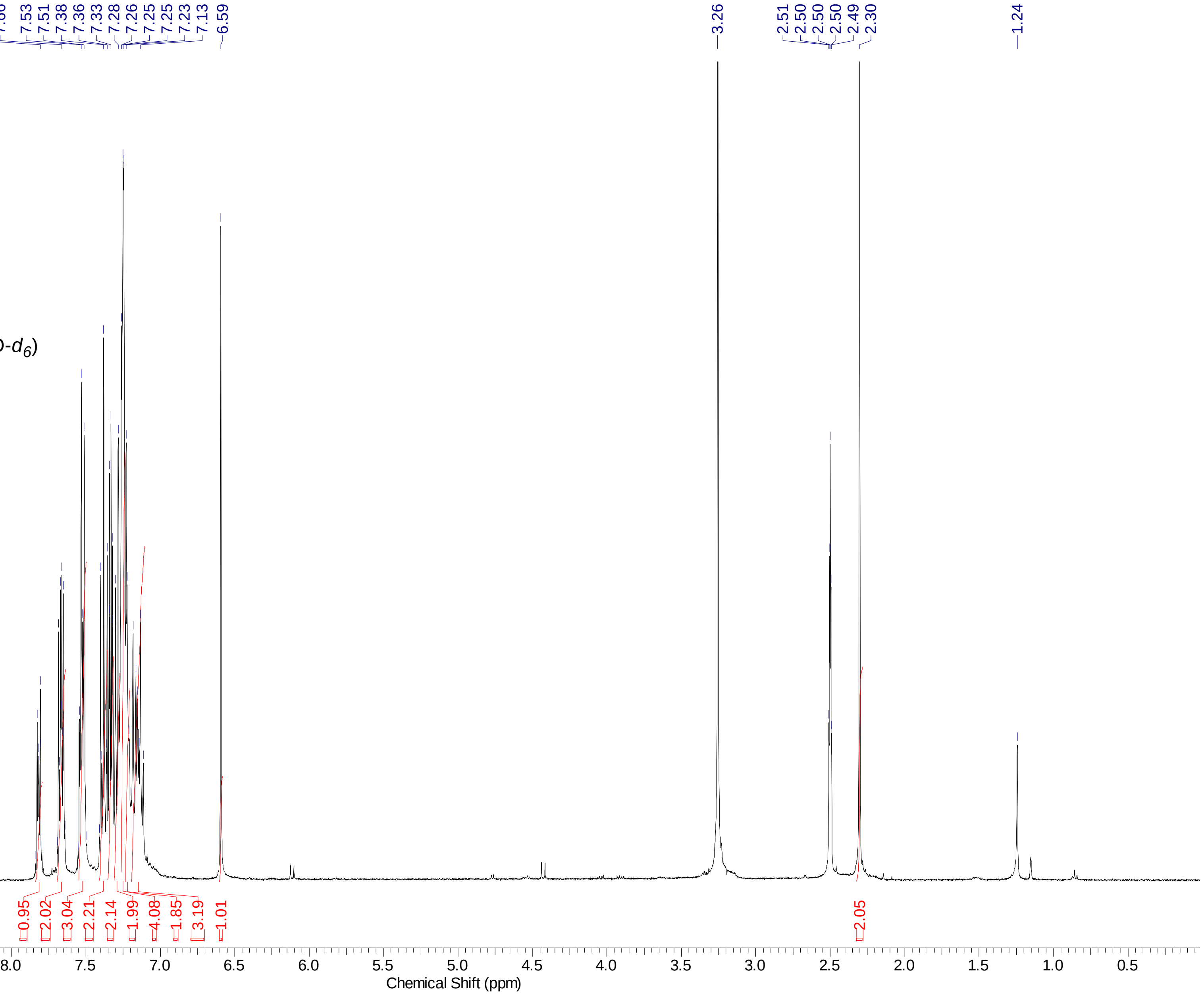

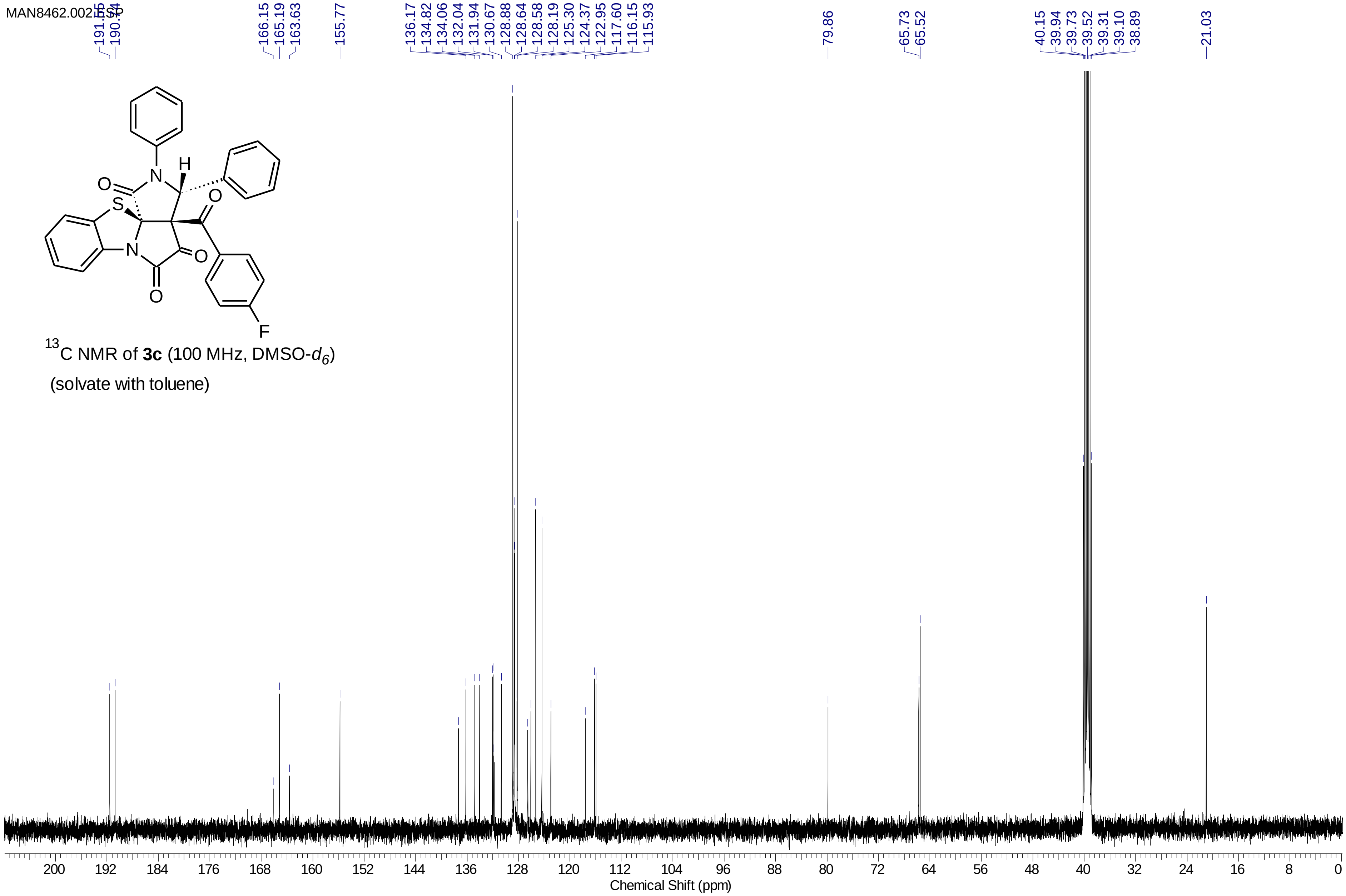


$^{13}$C NMR of **3c** (100 MHz, DMSO-$d_6$)
(solvate with toluene)

MAN8462.003.ESP

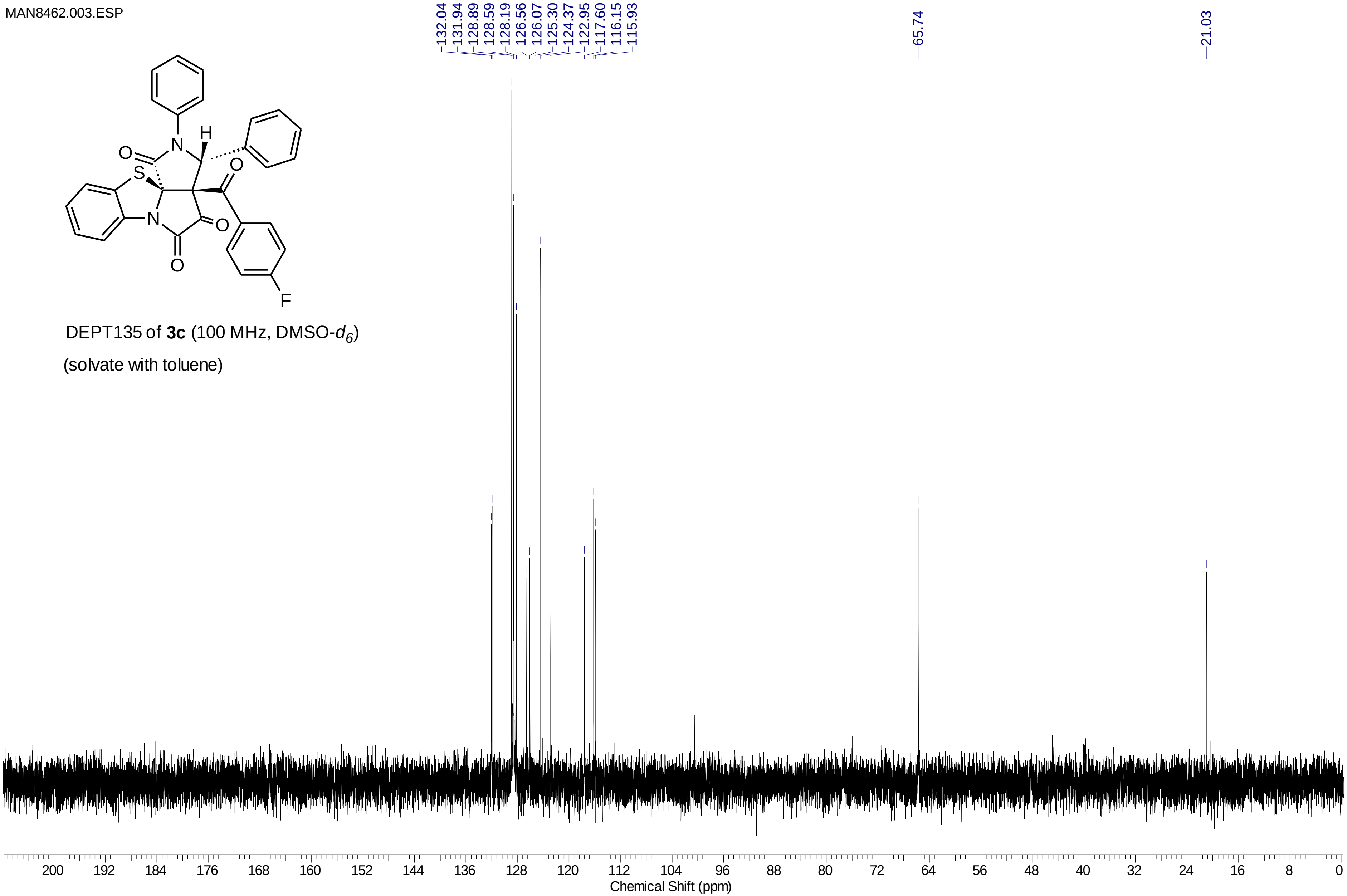


DEPT135 of **3c** (100 MHz, DMSO-$d_6$)

(solvate with toluene)

MAN8462.004.ESP

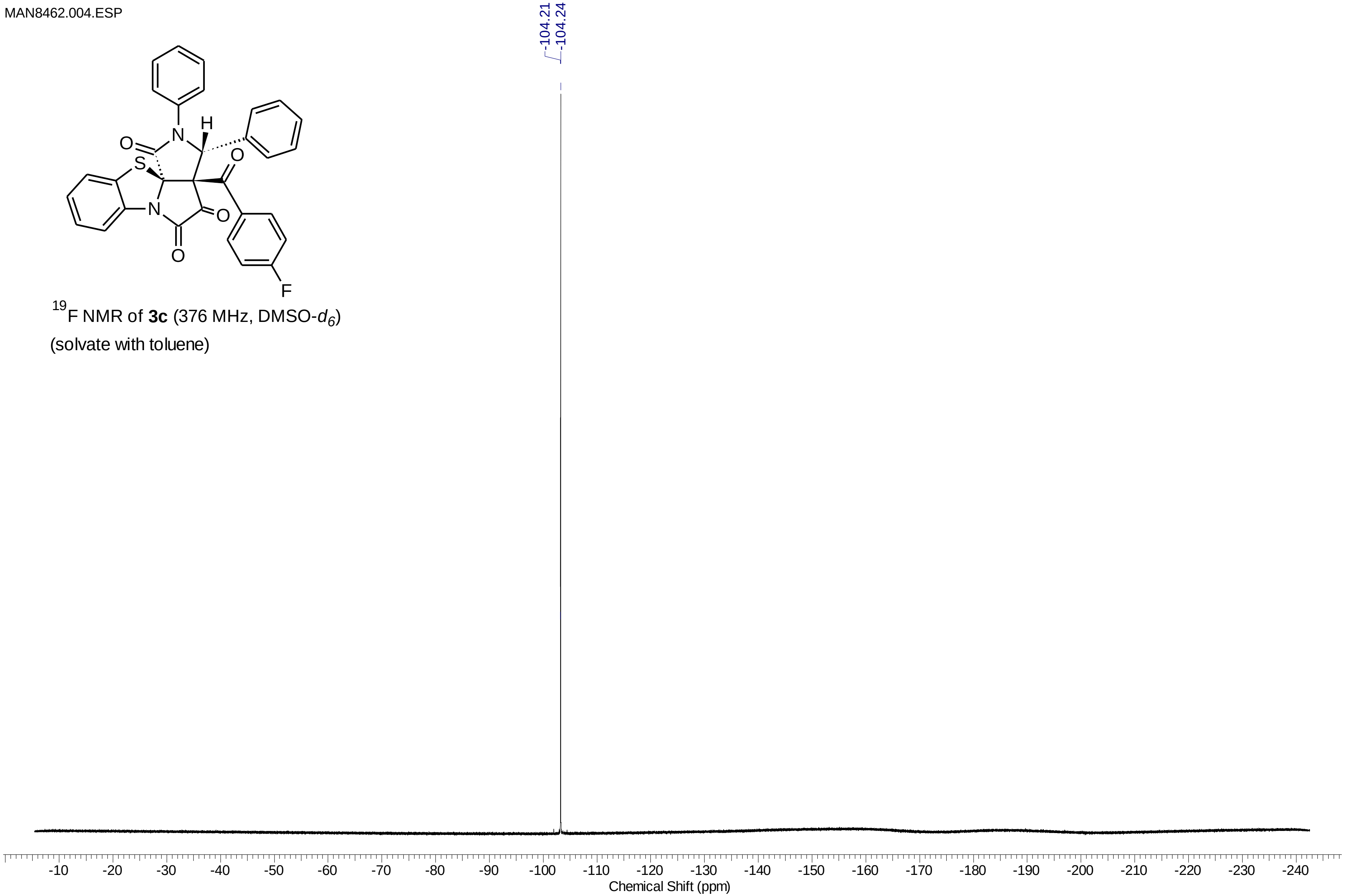


$^{19}$F NMR of **3c** (376 MHz, DMSO-$d_6$)

(solvate with toluene)

MAN8468.001.ESP

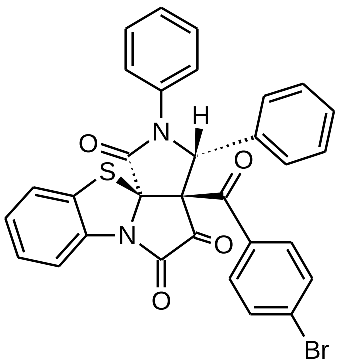


$^{1}$H NMR of **3d** (400 MHz, DMSO-$d_6$)

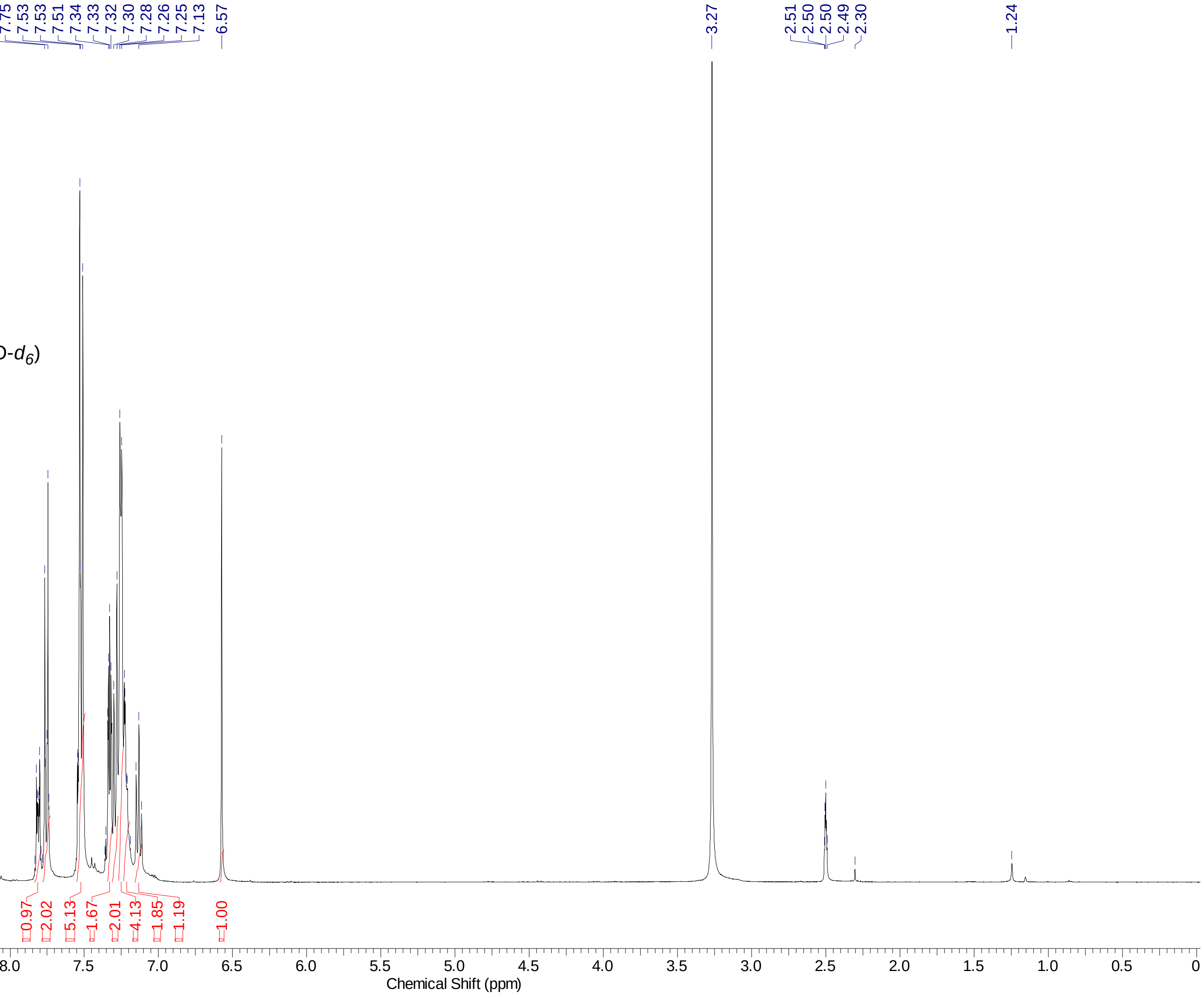

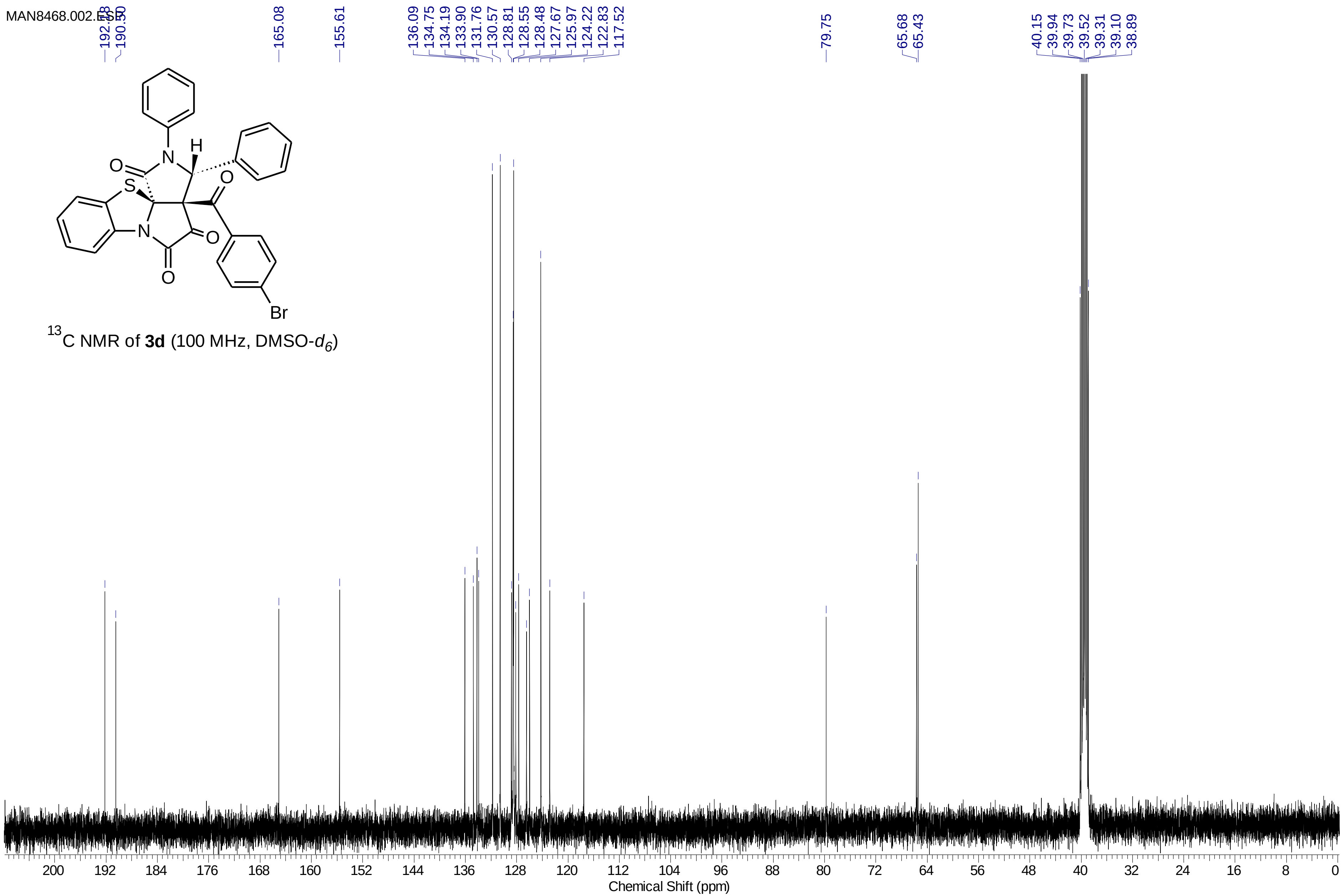


$^{13}C$ NMR of **3d** (100 MHz, DMSO-$d_6$)

MAN8468.003.ESP

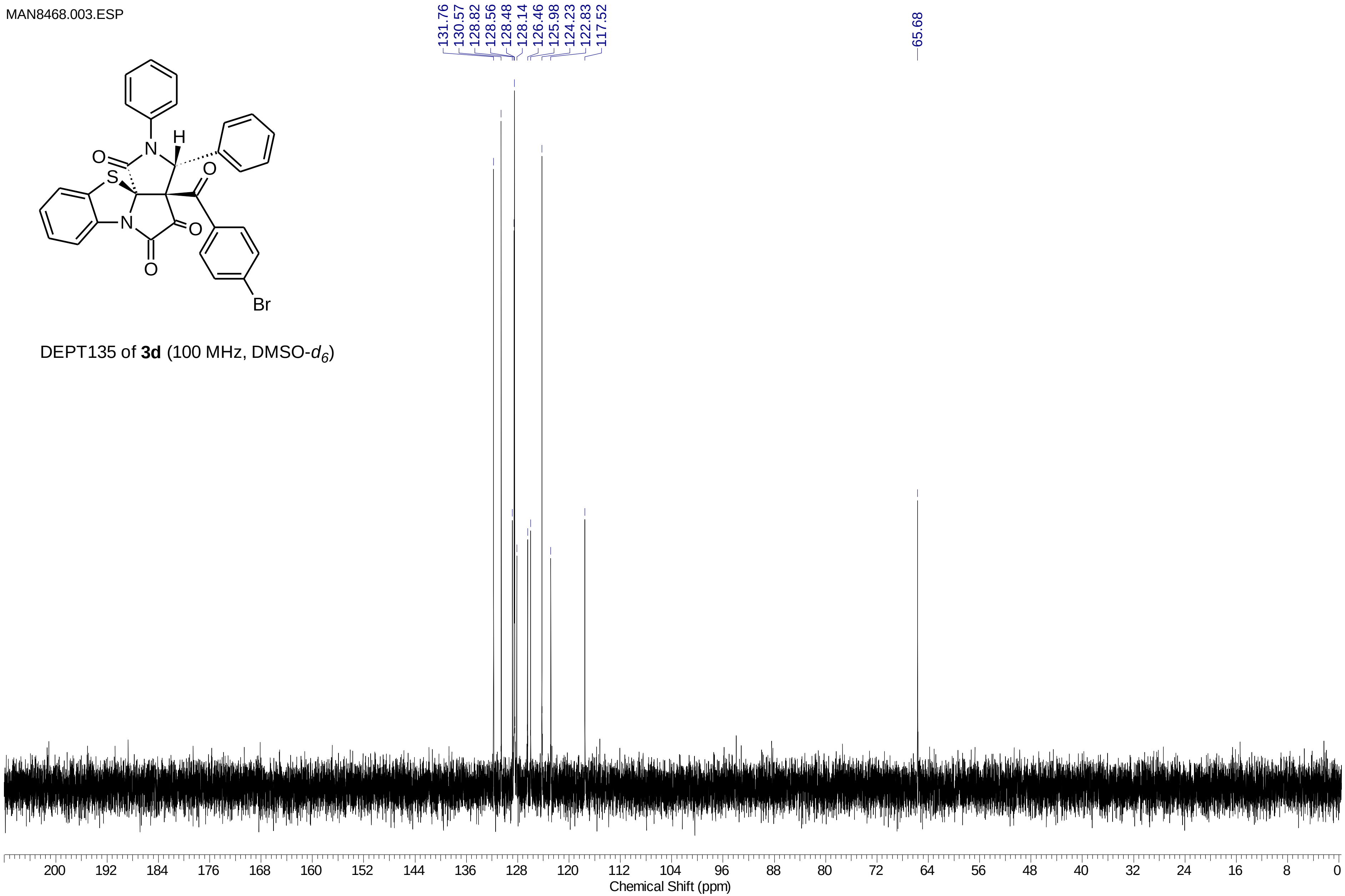


DEPT135 of **3d** (100 MHz, DMSO-$d_6$)

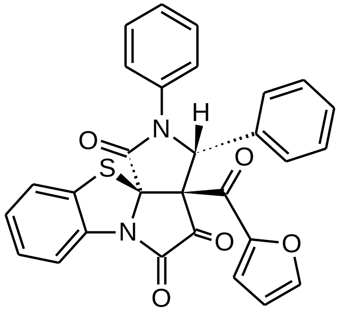

$^1$H NMR of **3e** (400 MHz, DMSO-$d_6$)
(solvate with toluene)

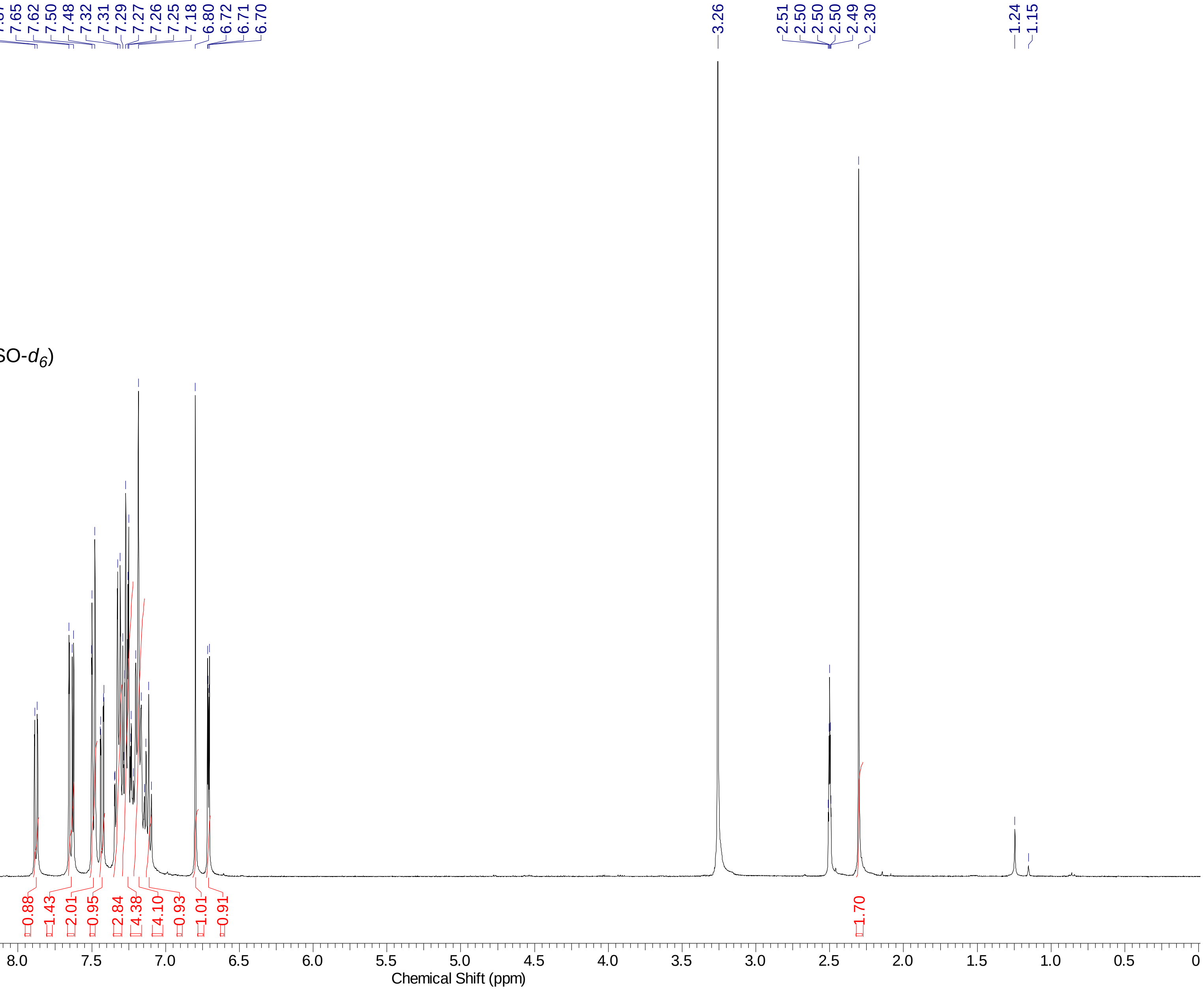

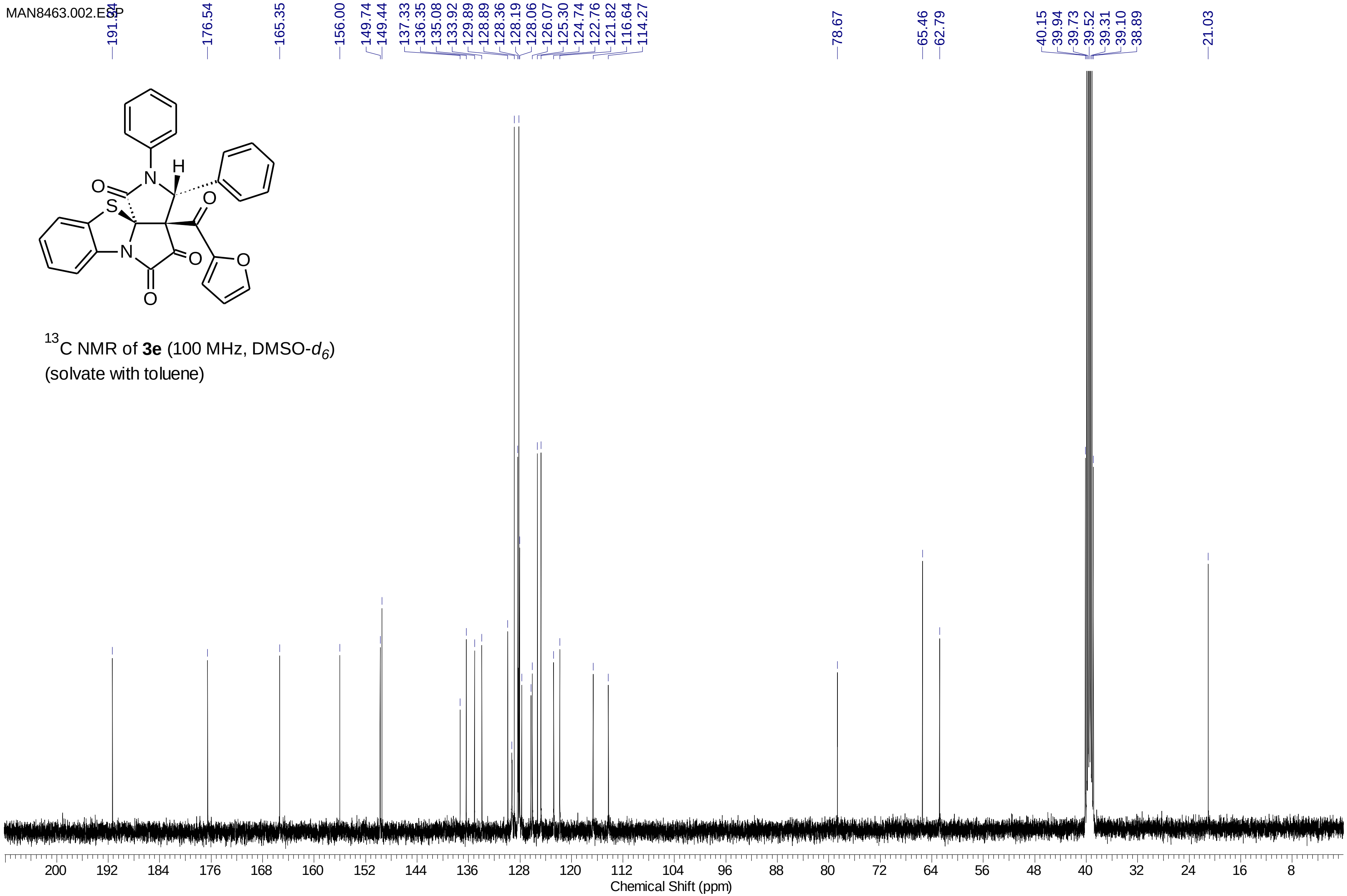


$^{13}$C NMR of **3e** (100 MHz, DMSO-$d_6$)
(solvate with toluene)

MAN8463.003.ESP

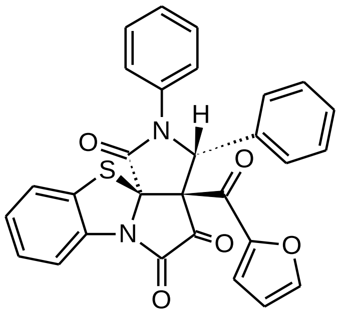

DEPT135 of **3e** (100 MHz, DMSO-$d_6$)
(solvate with toluene)

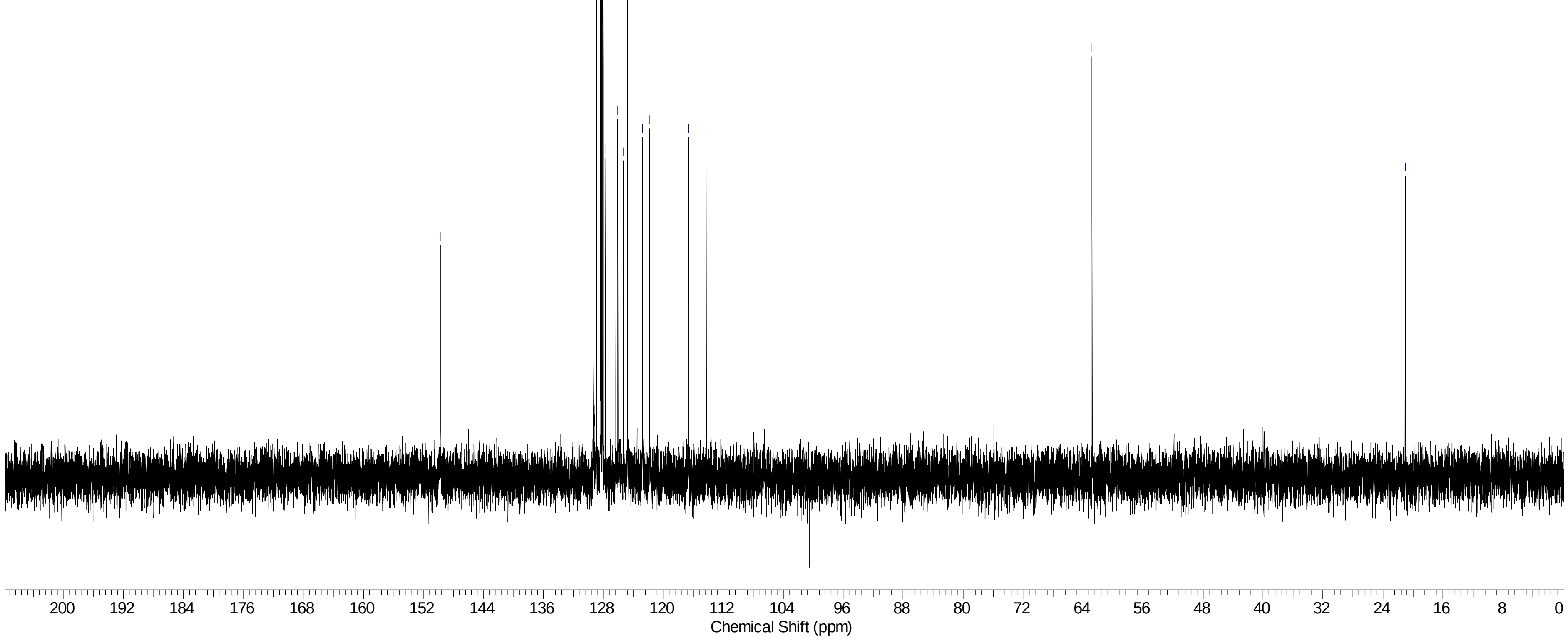

MAN8464.001.ESP

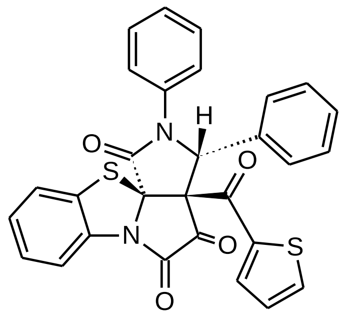

$^{1}$H NMR of **3f** (400 MHz, DMSO-$d_6$)

(solvate with toluene)

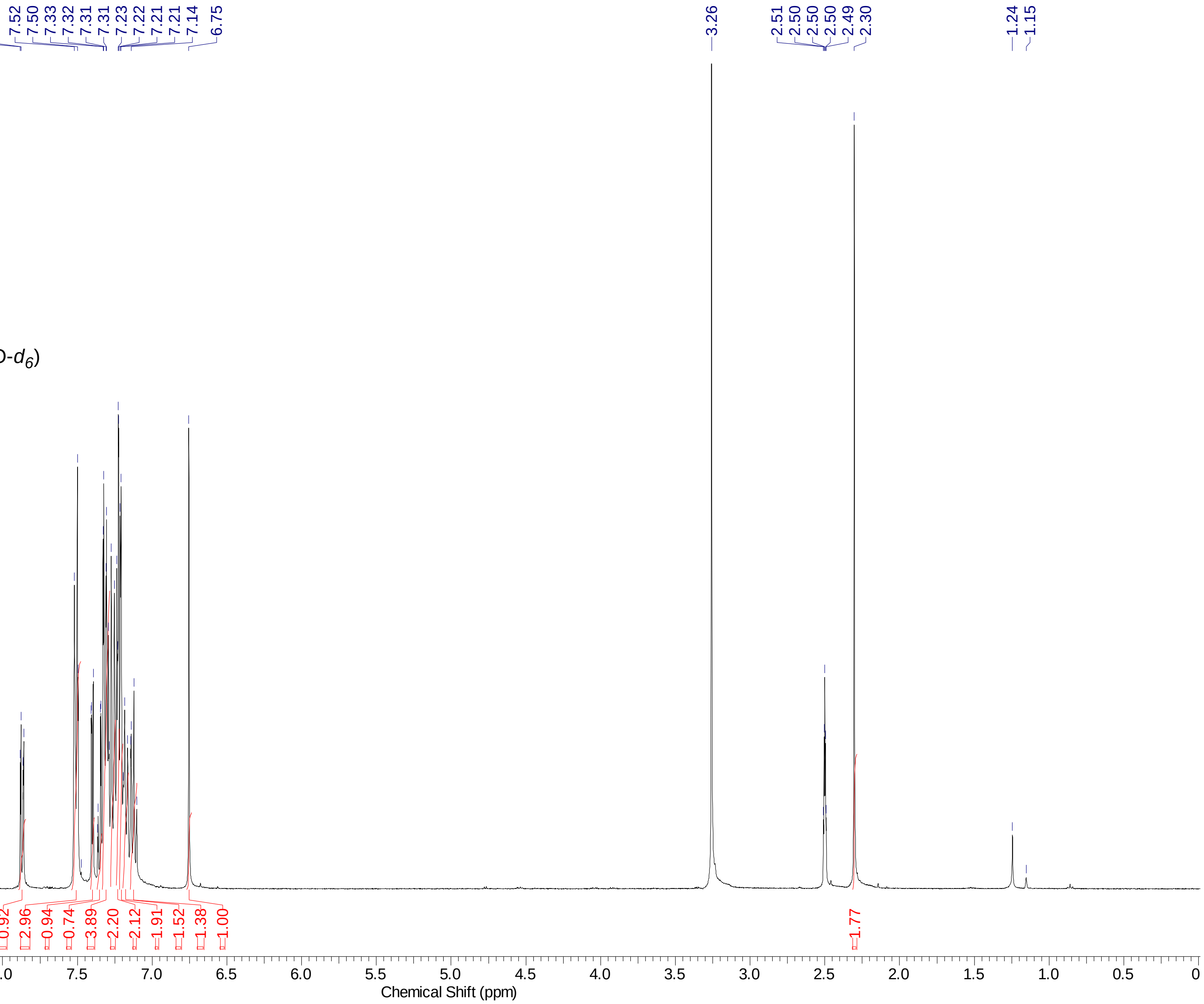

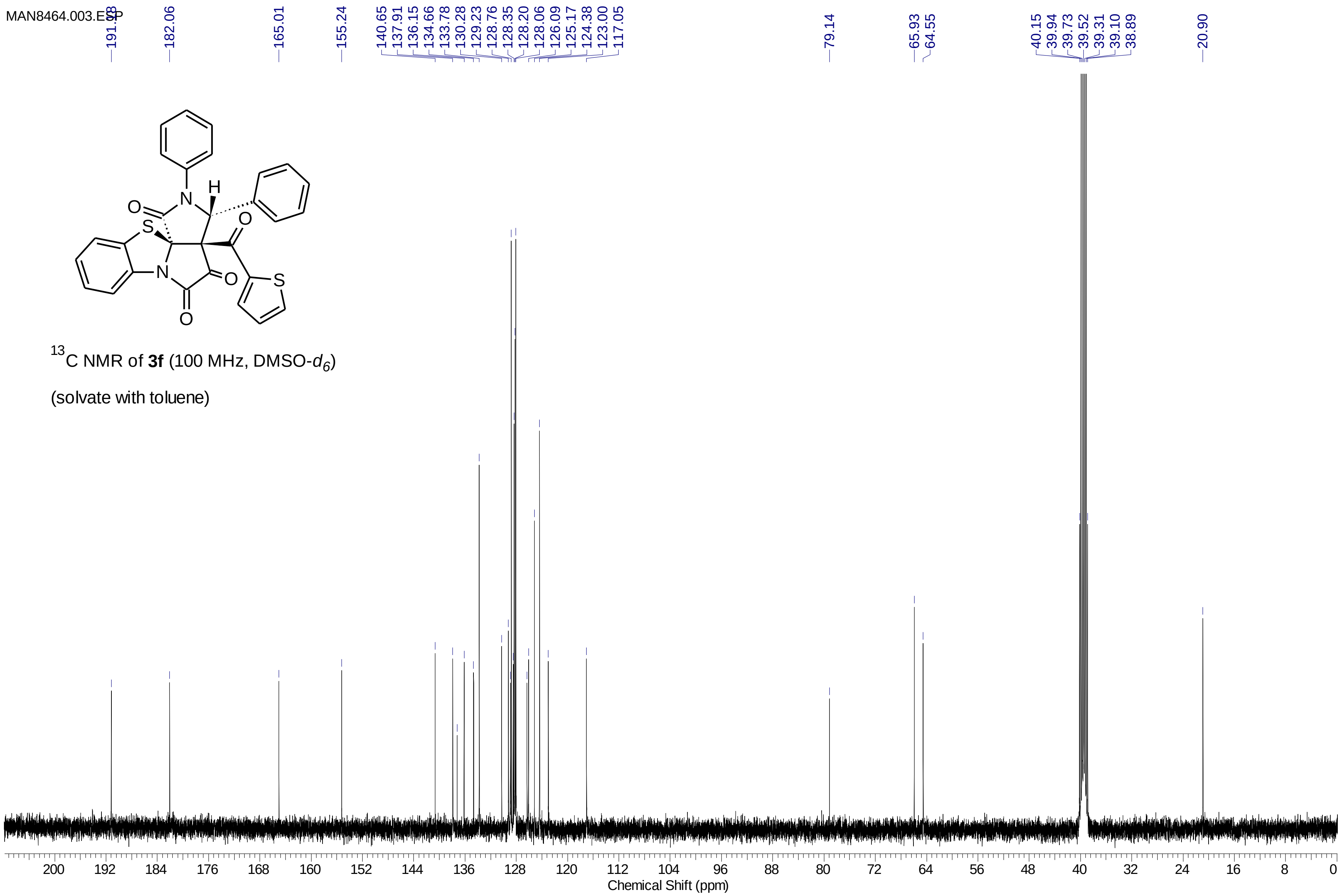


$^{13}$C NMR of **3f** (100 MHz, DMSO-$d_6$)

(solvate with toluene)

MAN8464.004.ESP

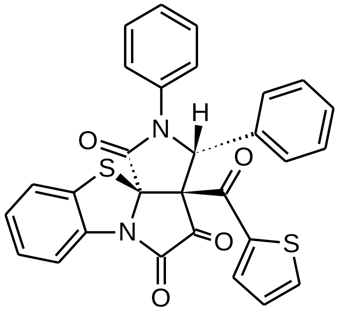

DEPT135 of **3f** (100 MHz, DMSO-$d_6$)

(solvate with toluene)

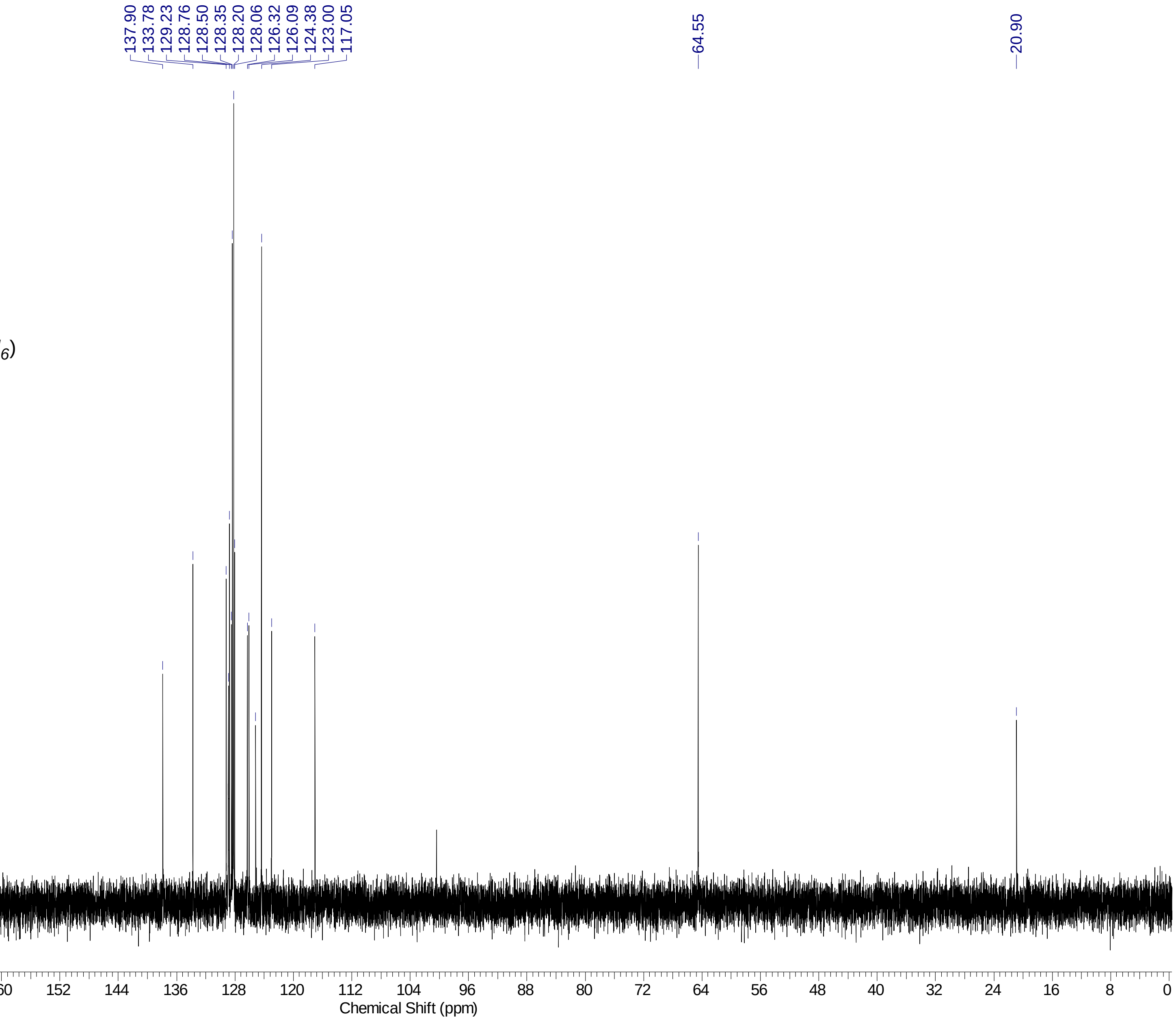

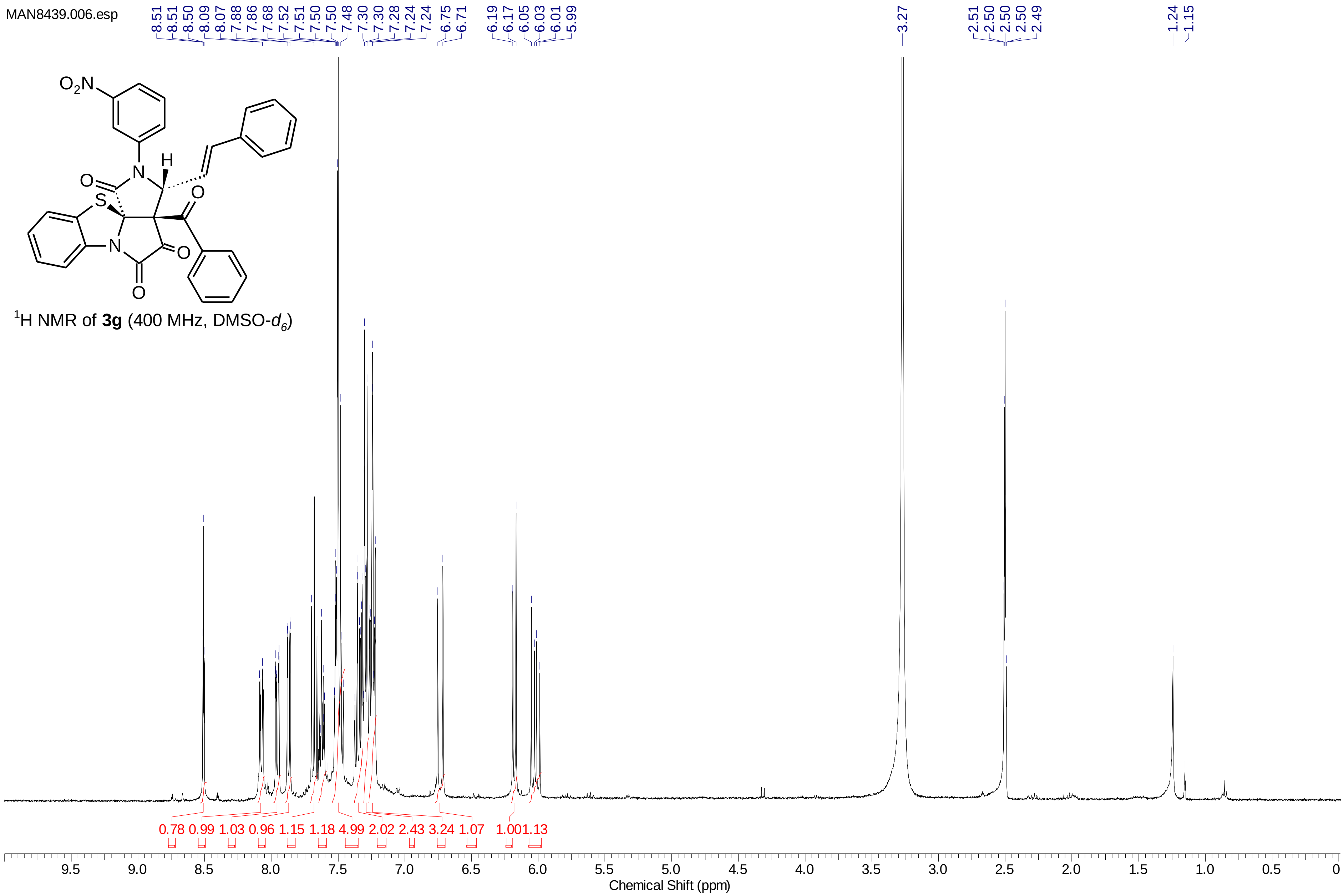


$^{1}$H NMR of **3g** (400 MHz, DMSO-$d_6$)

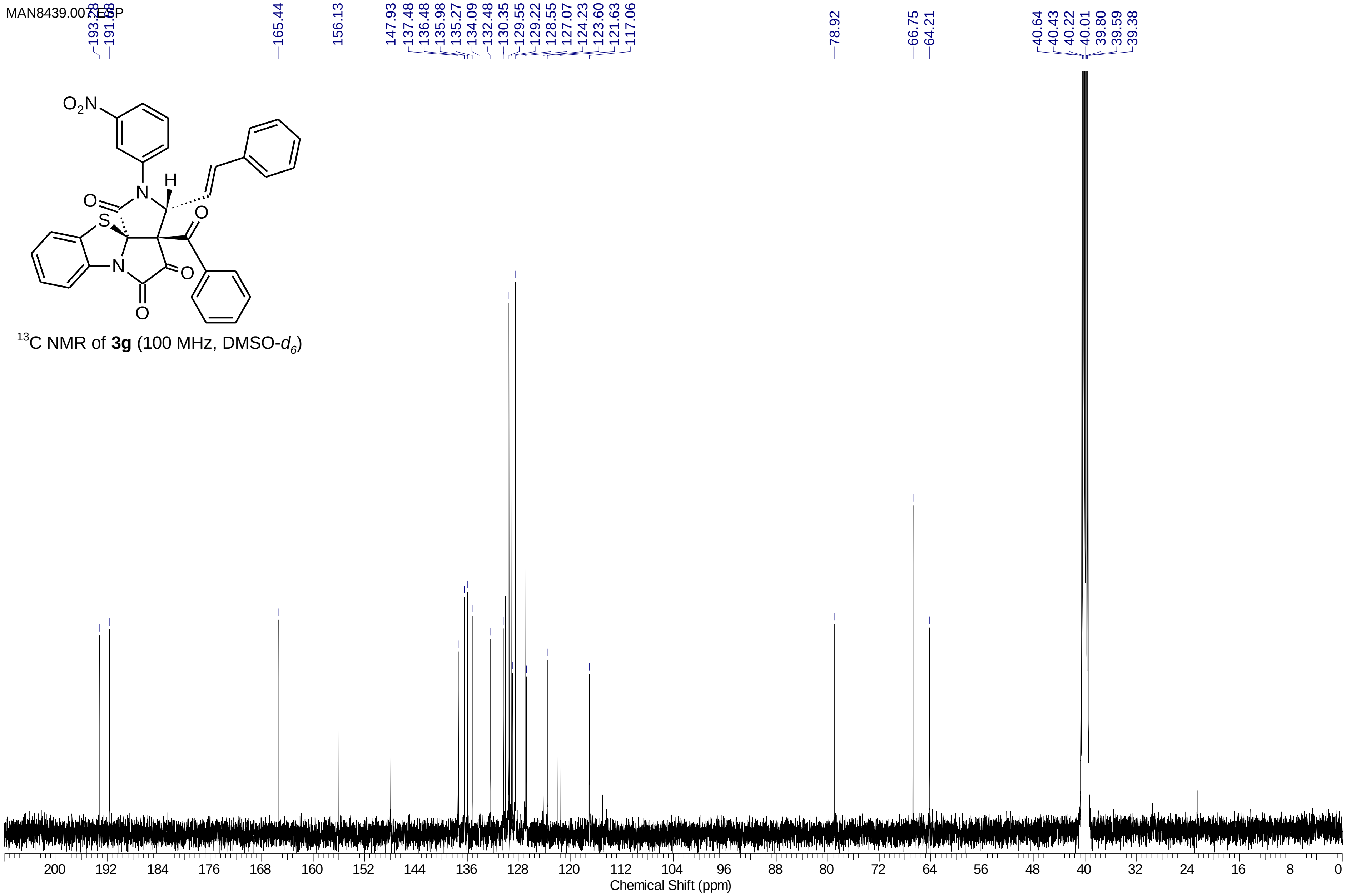


$^{13}$C NMR of **3g** (100 MHz, DMSO-$d_6$)

MAN8439.008.esp

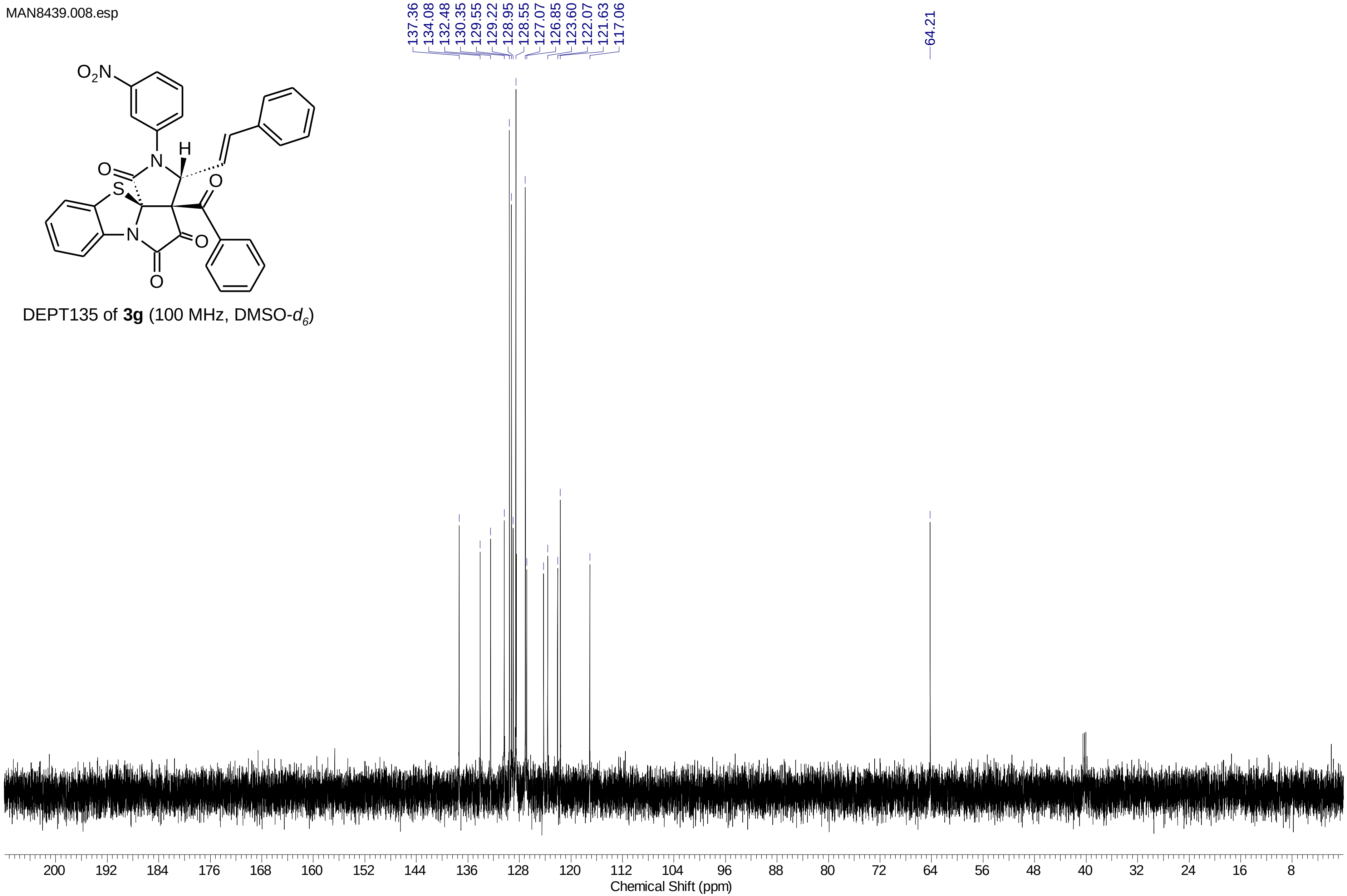

MAN8472.001.ESP

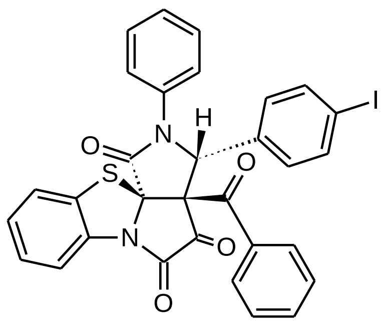

$^{1}$H NMR of **3h** (400 MHz, DMSO-$d_6$)

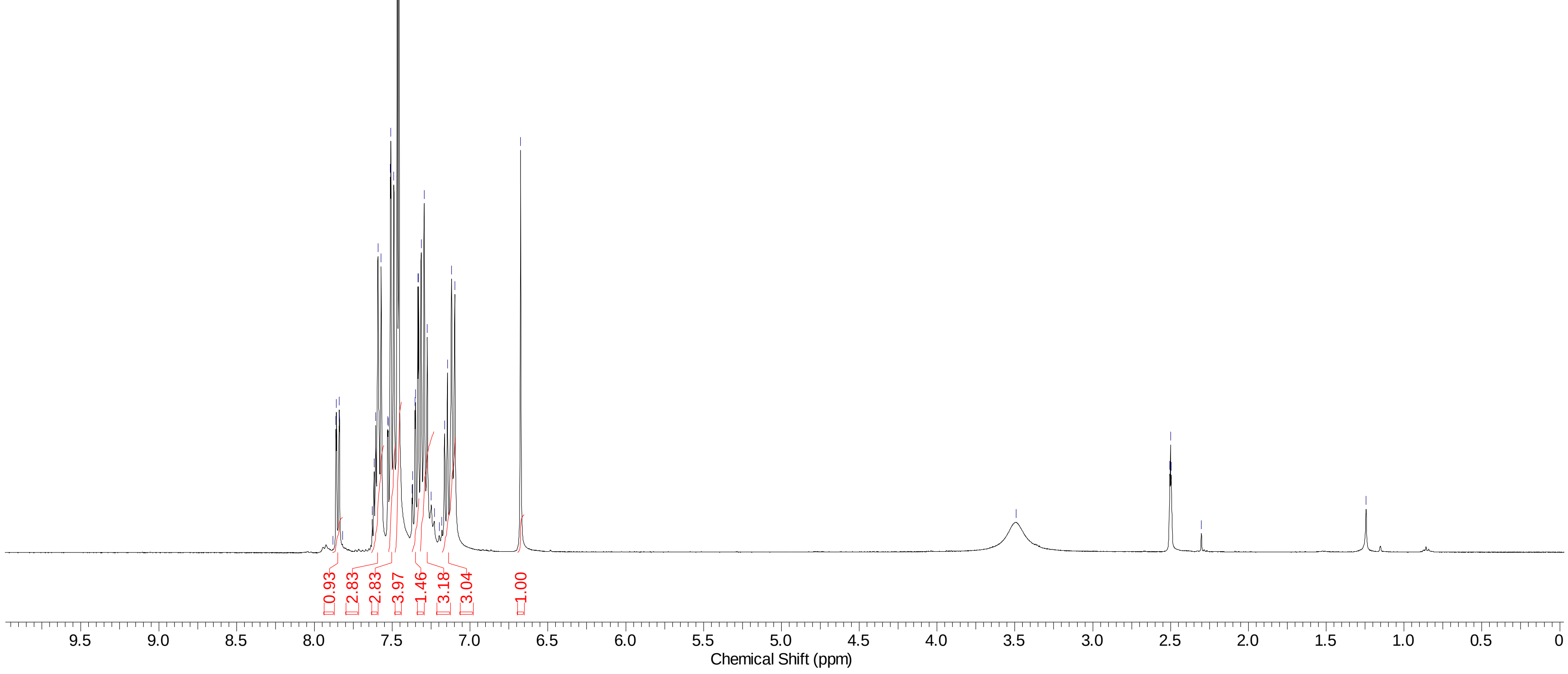

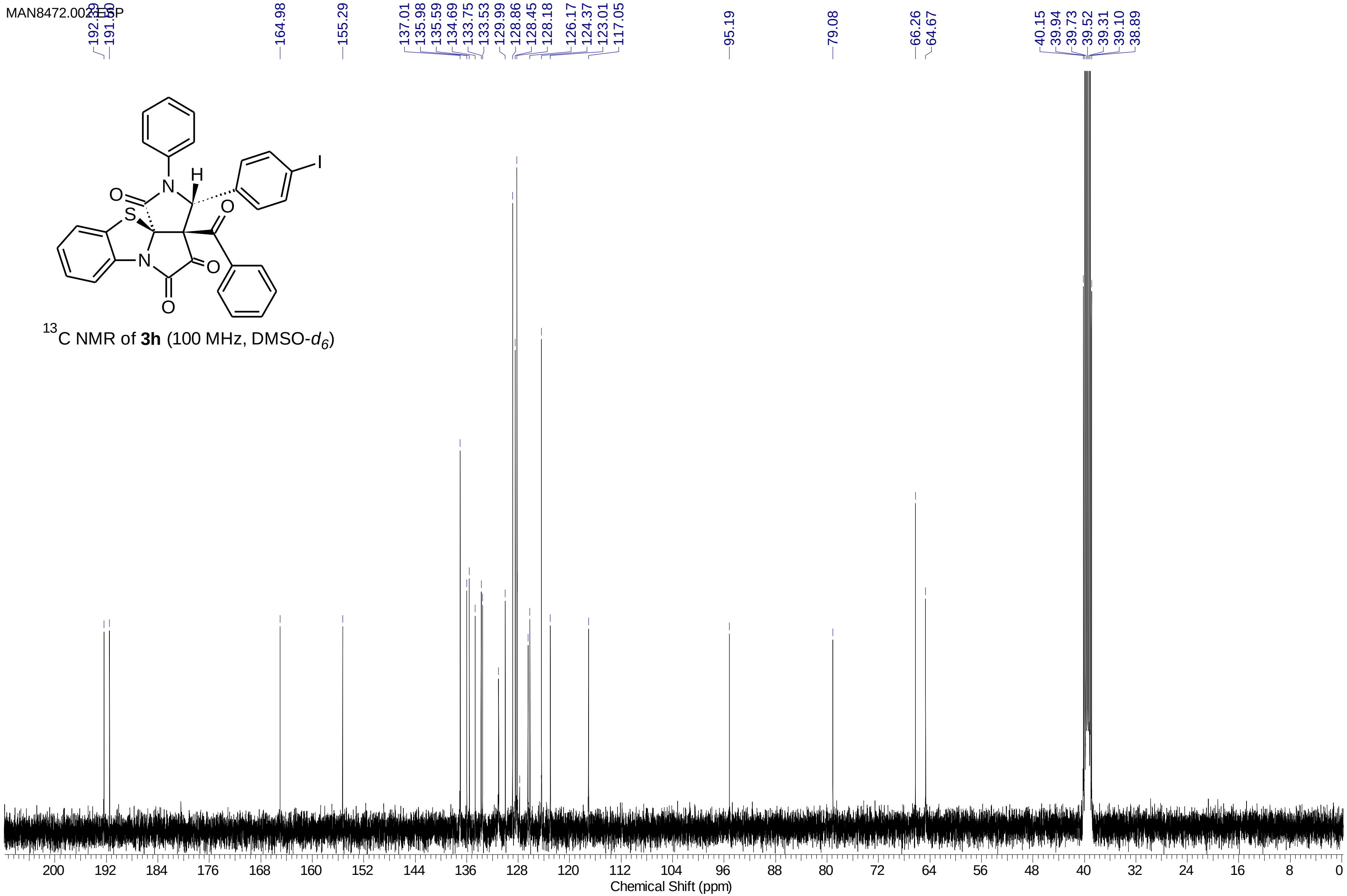


$^{13}$C NMR of **3h** (100 MHz, DMSO-$d_6$)

MAN8472.003.ESP

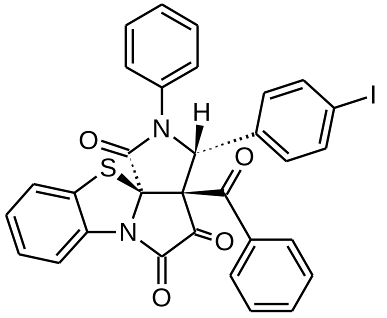

DEPT135 of **3h** (100 MHz, DMSO-$d_6$)

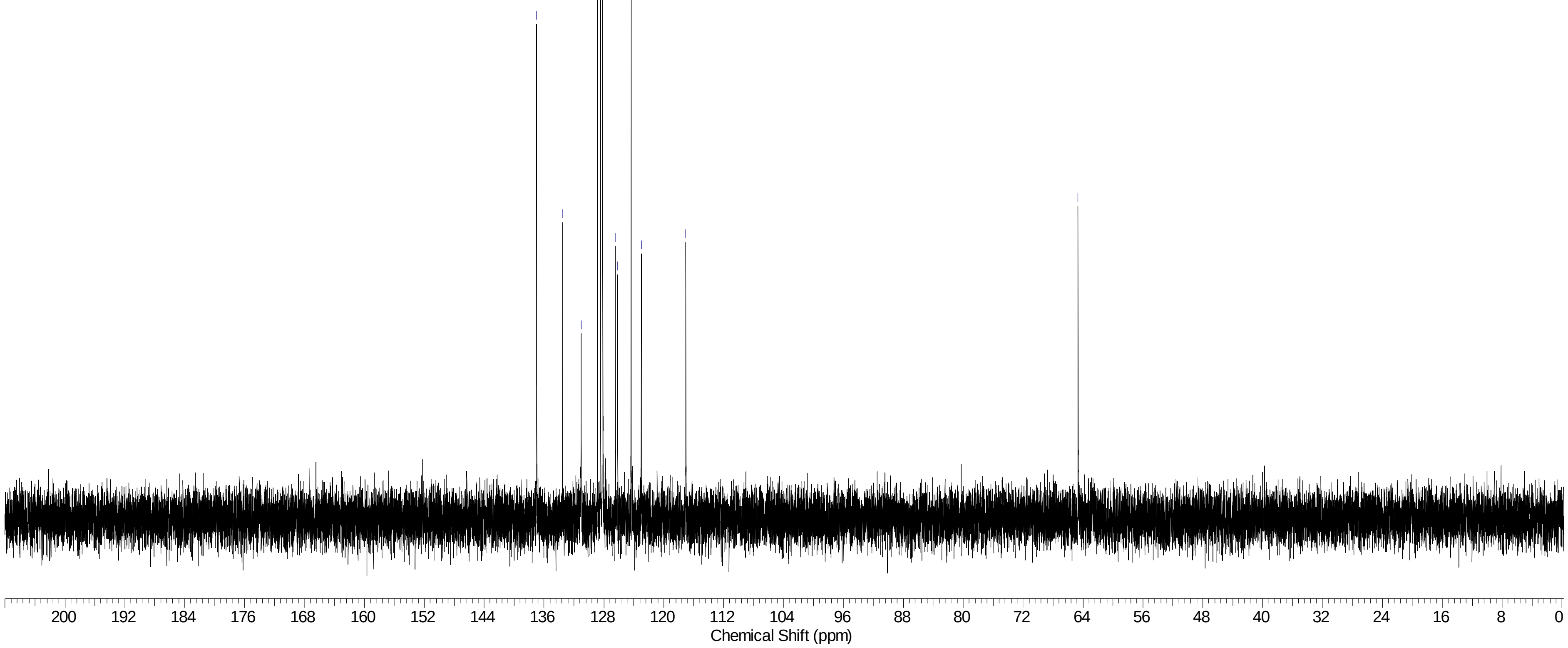

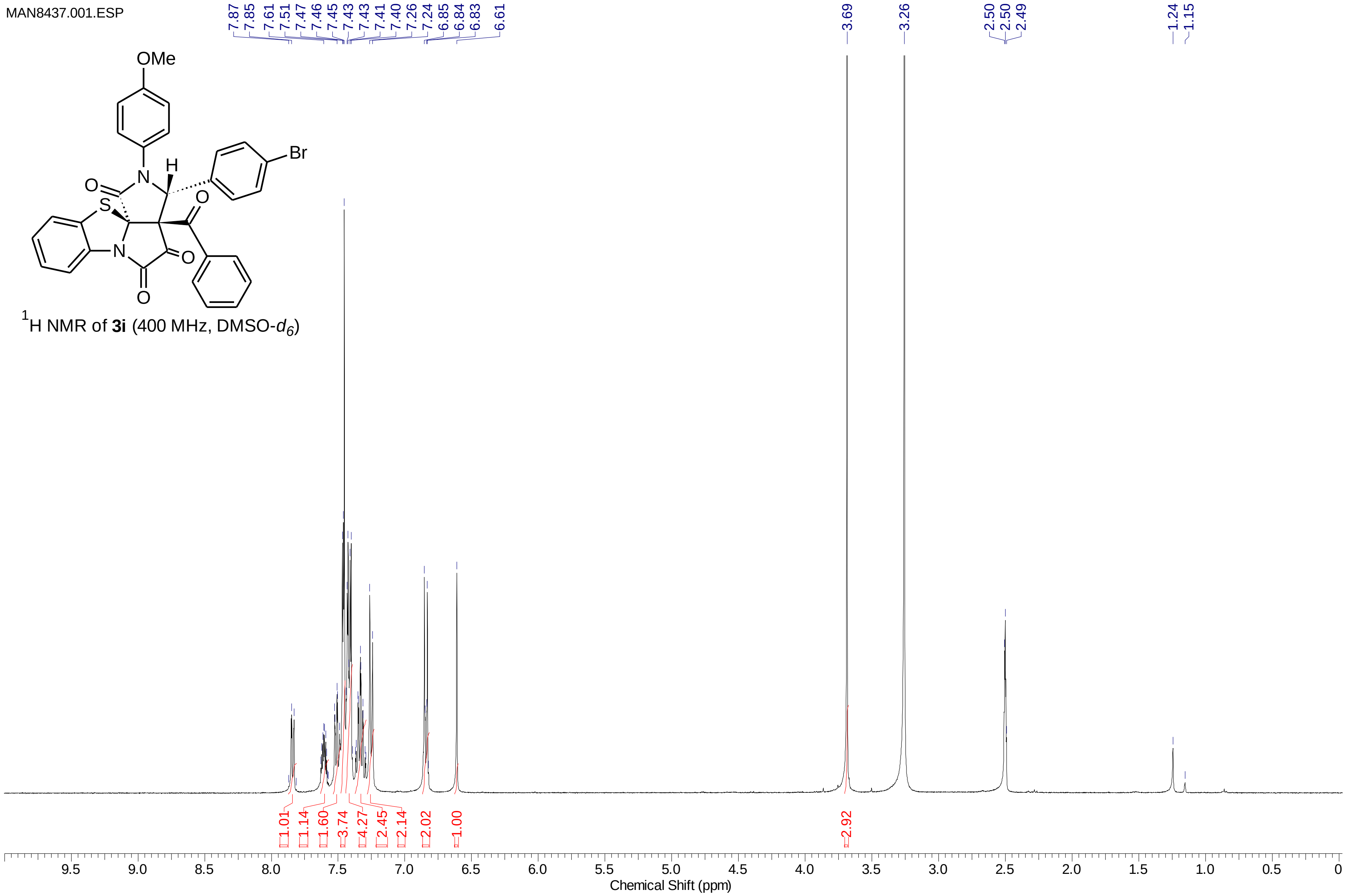


$^{1}$H NMR of **3i** (400 MHz, DMSO-$d_6$)

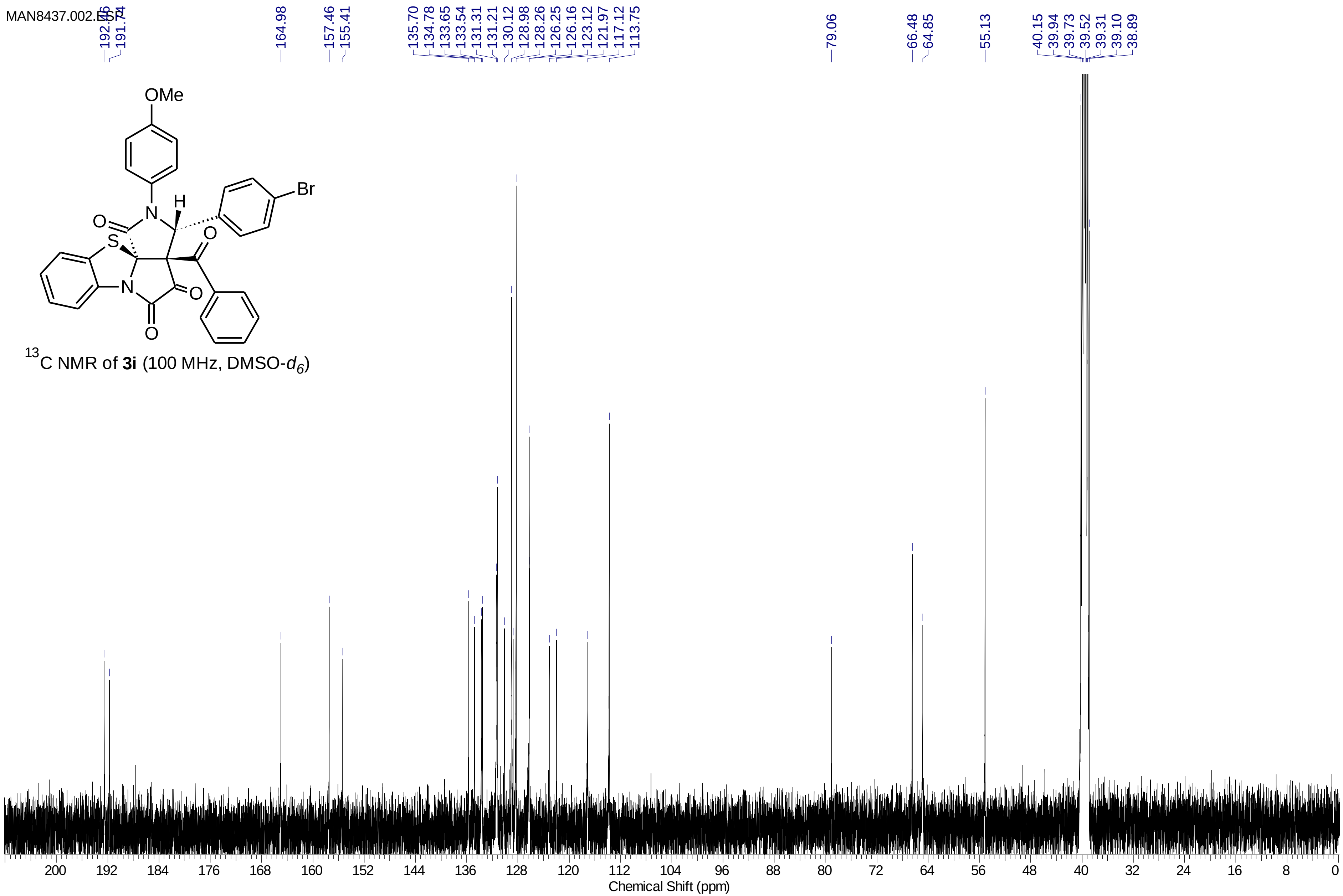


$^{13}$C NMR of **3i** (100 MHz, DMSO-$d_6$)

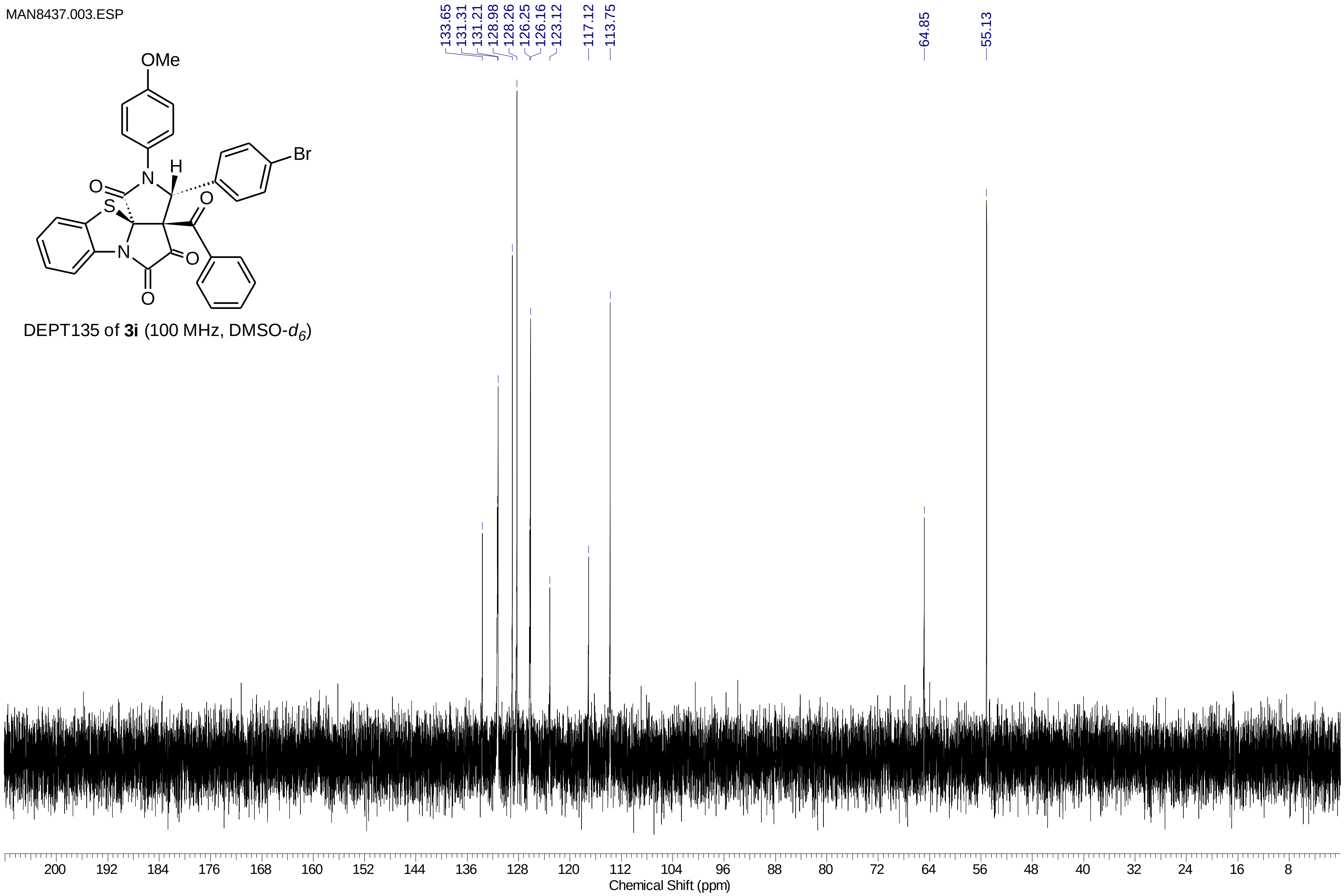

DEPT135 of **3i** (100 MHz, DMSO-$d_6$)

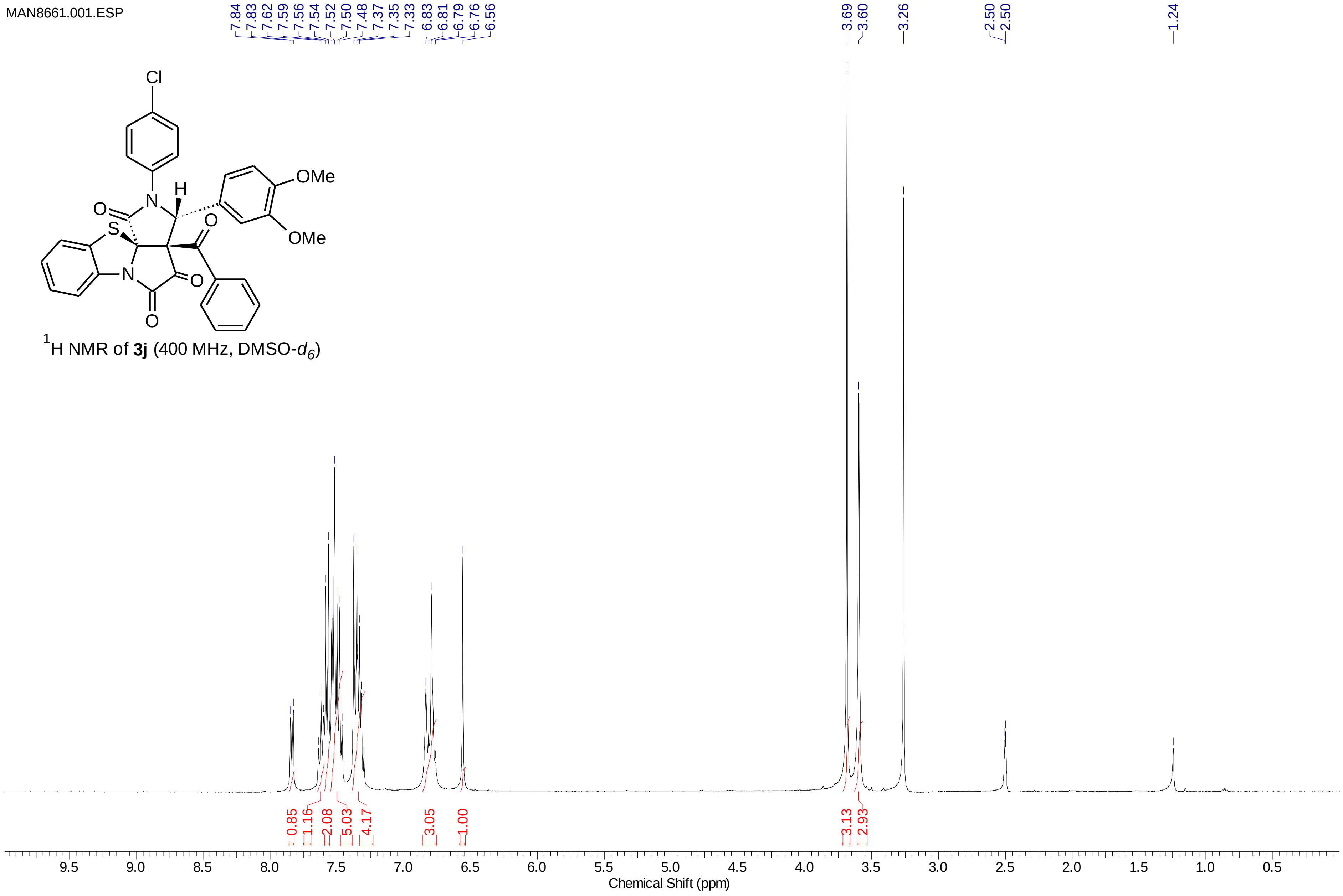


$^{1}$H NMR of **3j** (400 MHz, DMSO-$d_6$)

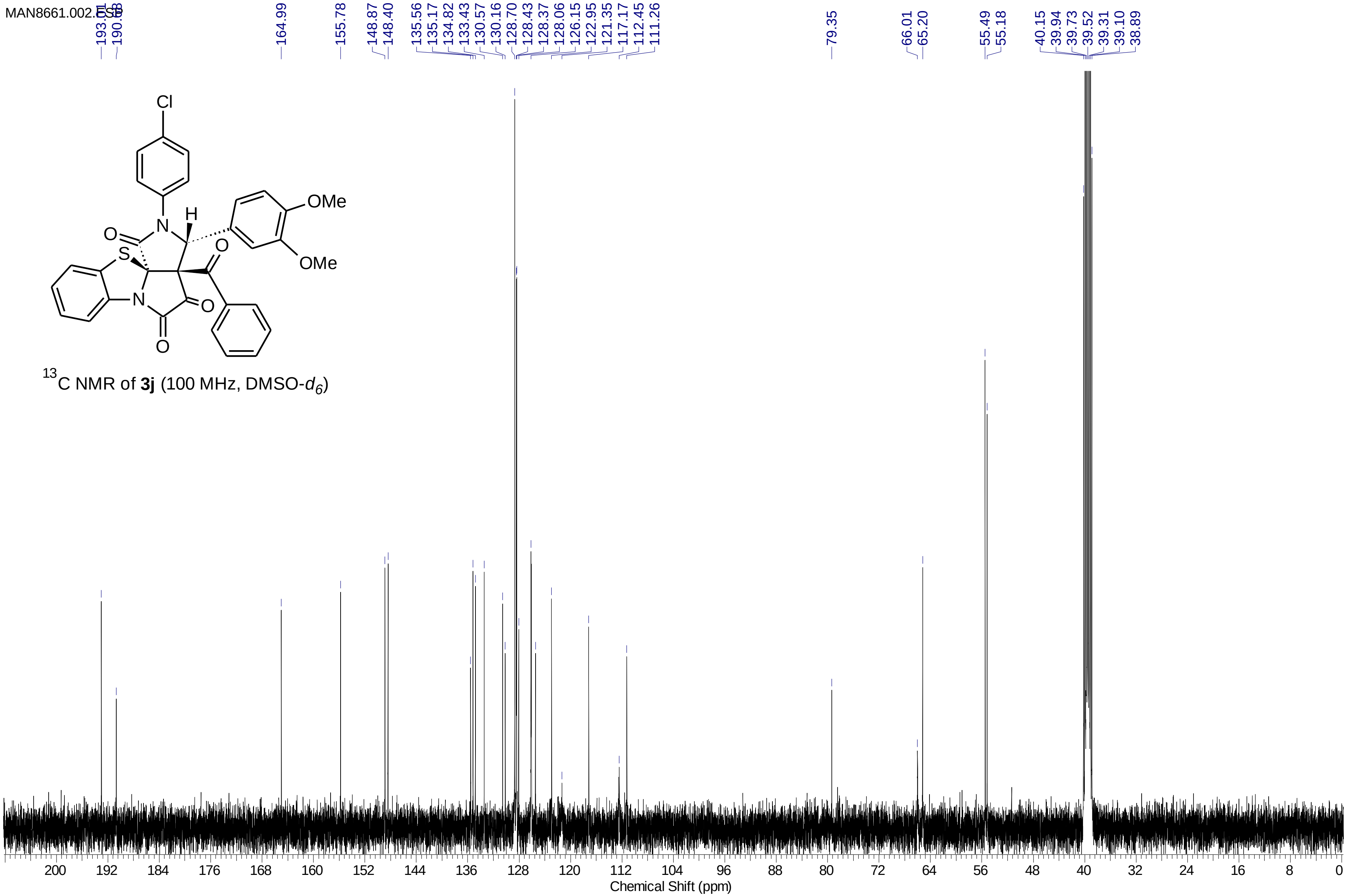


$^{13}$C NMR of **3j** (100 MHz, DMSO-$d_6$)

MAN8661.003.ESP

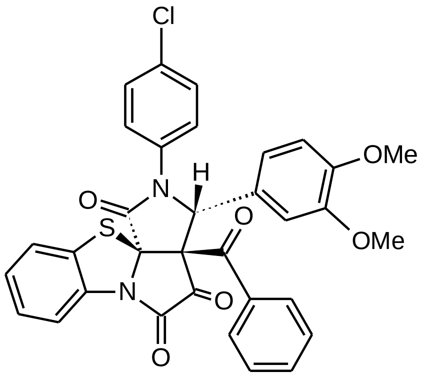

DEPT135 of **3j** (100 MHz, DMSO-$d_6$)

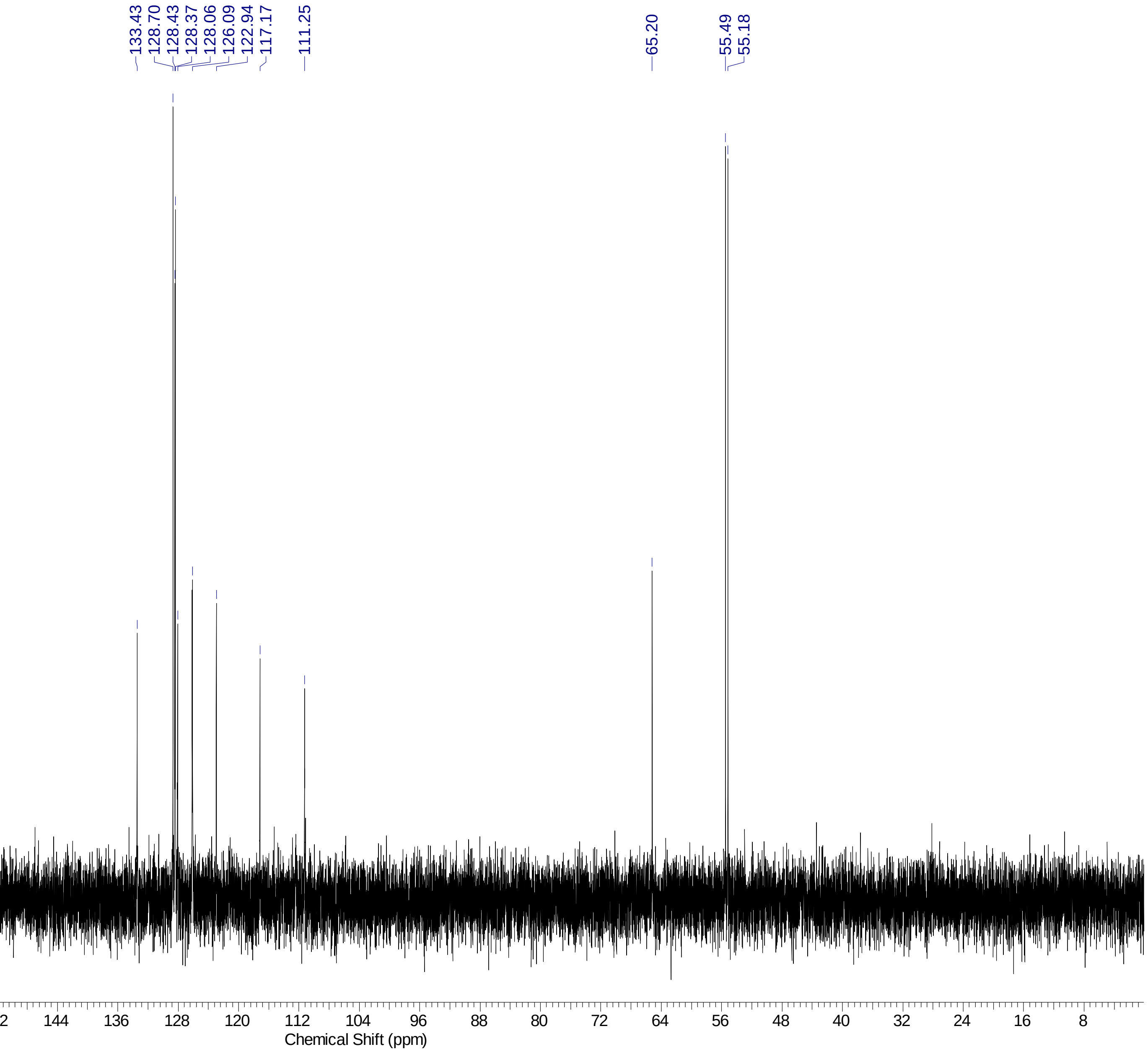

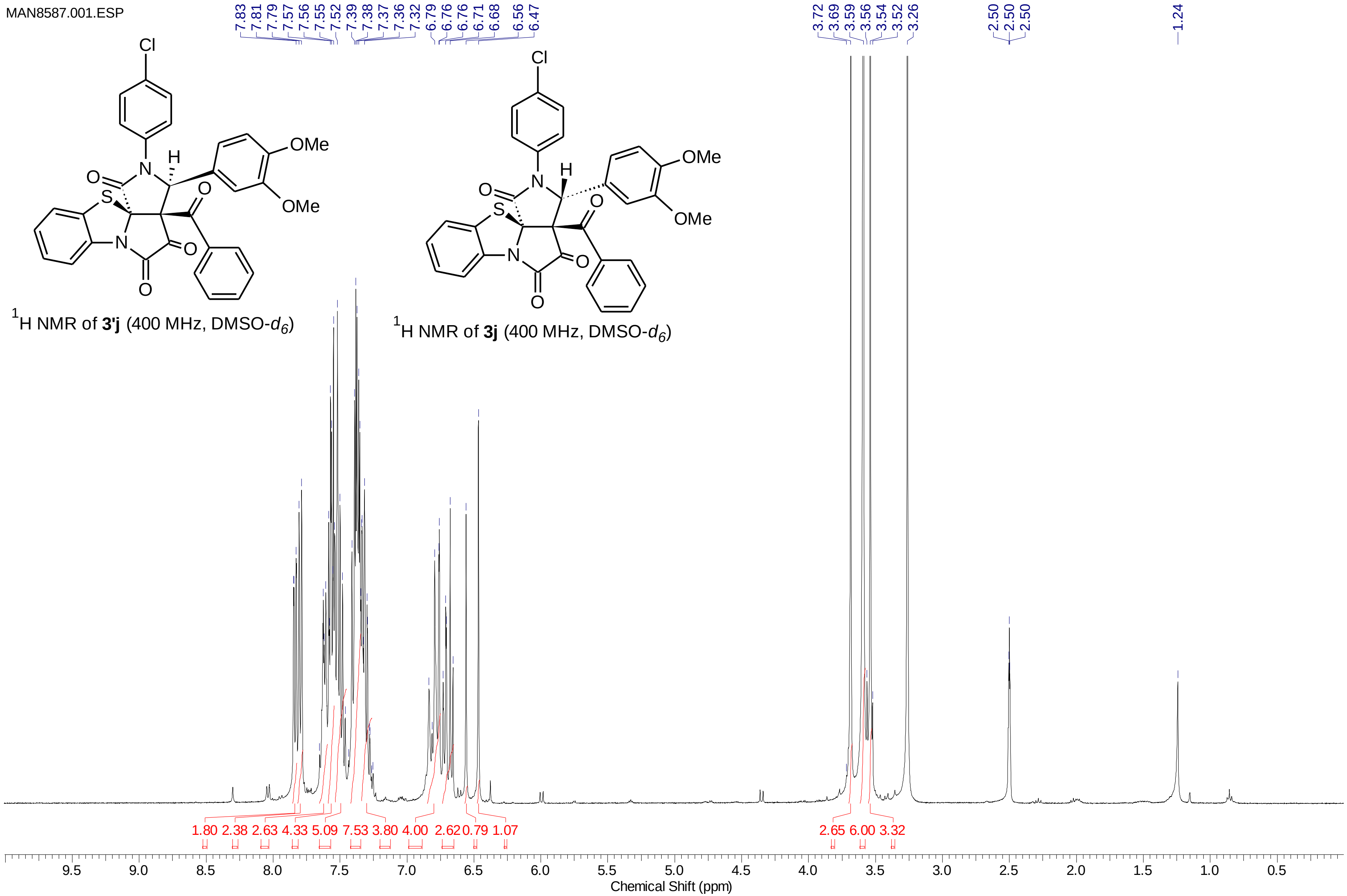
MAN8587.001.ESP
7.83 7.81 7.79 7.57 7.56 7.55 7.52 7.39 7.38 7.37 7.36 7.32 6.79 6.76 6.76 6.71 6.68 6.56 6.47
3.72 3.69 3.59 3.56 3.54 3.52 3.26
2.50 2.50 2.50
1.24
Cl
OMe
OMe
H
O
N
S
O
O
O
1H NMR of 3'j (400 MHz, DMSO-d6)
1H NMR of 3j (400 MHz, DMSO-d6)
1.80 2.38 2.63 4.33 5.09 7.53 3.80 4.00 2.62 0.79 1.07
2.65 6.00 3.32
9.5 9.0 8.5 8.0 7.5 7.0 6.5 6.0 5.5 5.0 4.5 4.0 3.5 3.0 2.5 2.0 1.5 1.0 0.5
Chemical Shift (ppm)

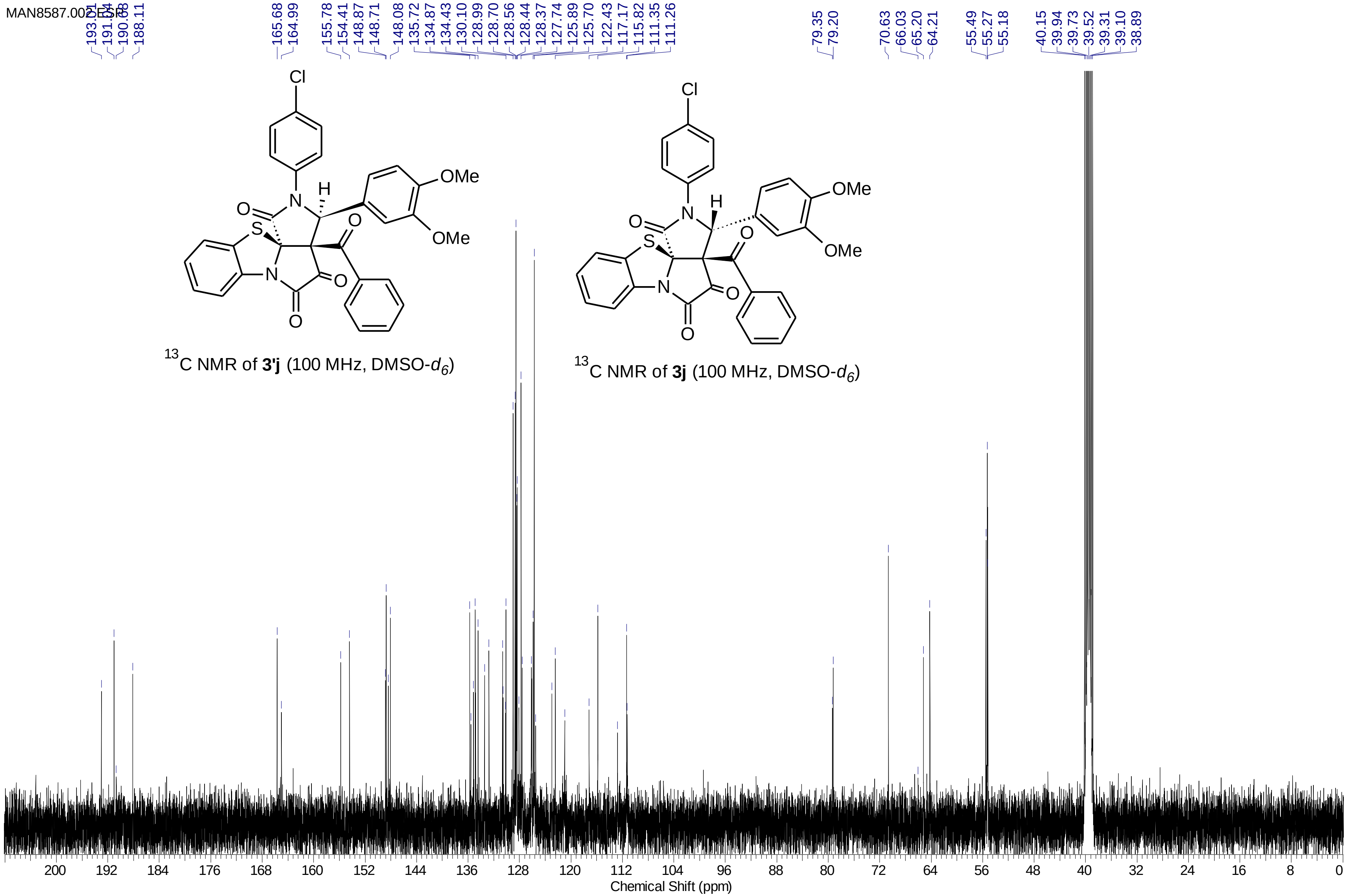


$^{13}$C NMR of **3'j** (100 MHz, DMSO-$d_6$)

$^{13}$C NMR of **3j** (100 MHz, DMSO-$d_6$)

MAN8587.003.ESP

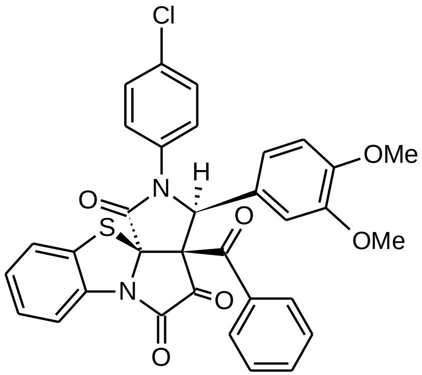

DEPT135 of **3'j** (100 MHz, DMSO-$d_6$)

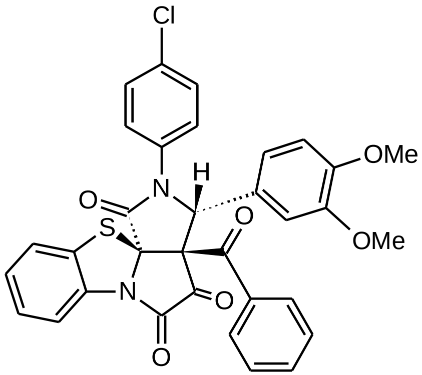

DEPT135 of **3j** (100 MHz, DMSO-$d_6$)

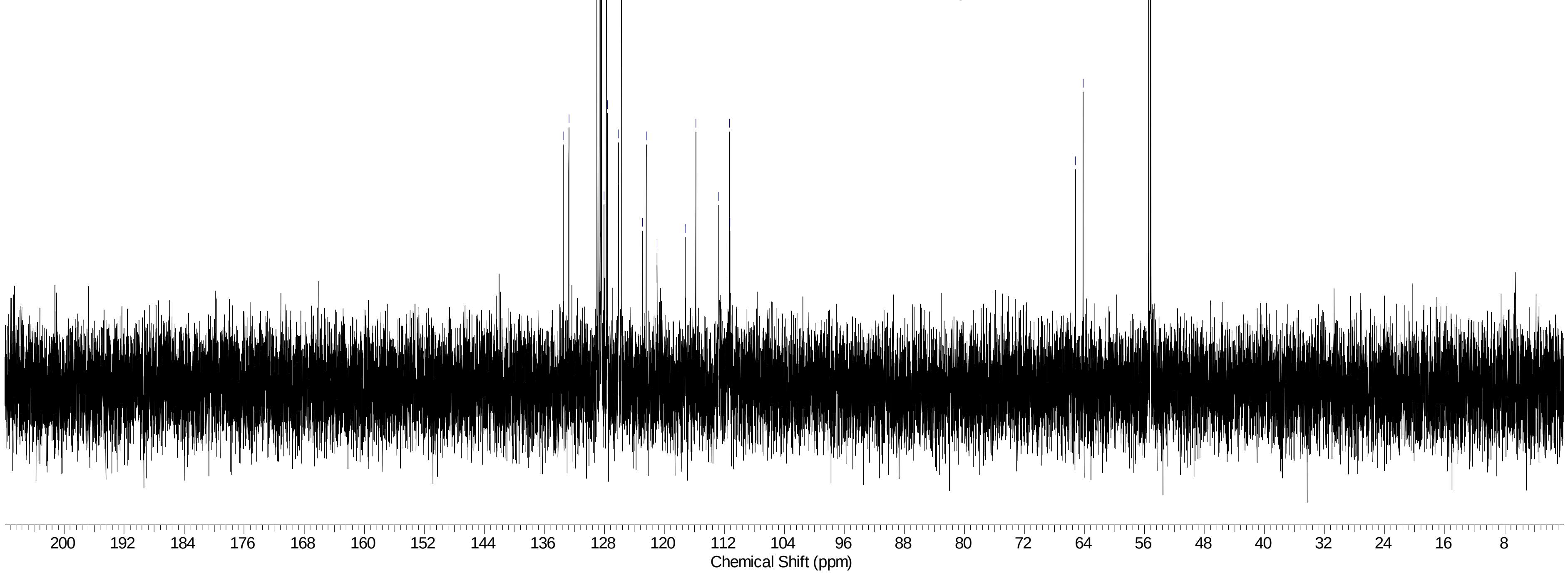

MAN8405.001.ESP

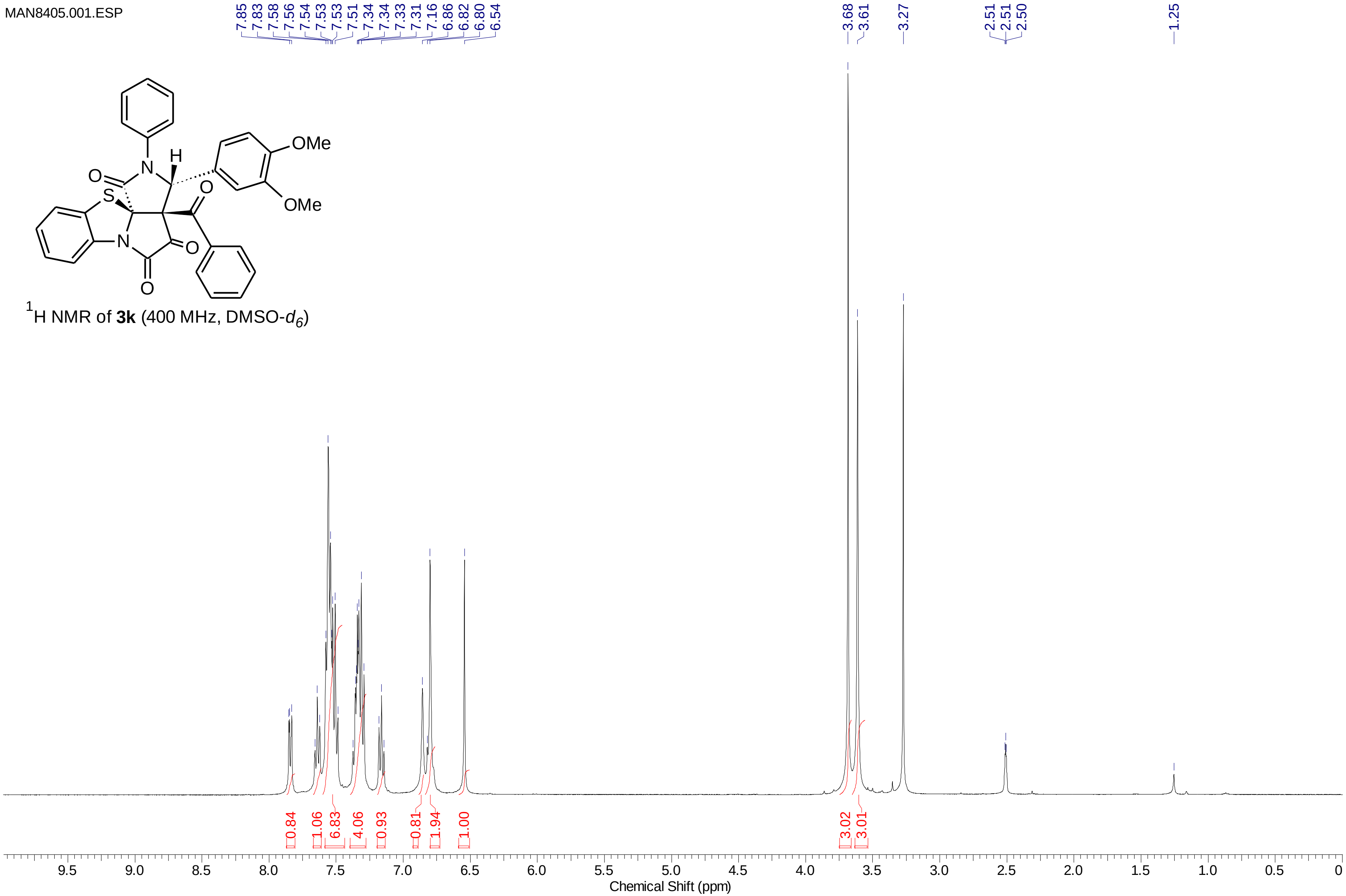


$^{1}$H NMR of **3k** (400 MHz, DMSO-$d_6$)

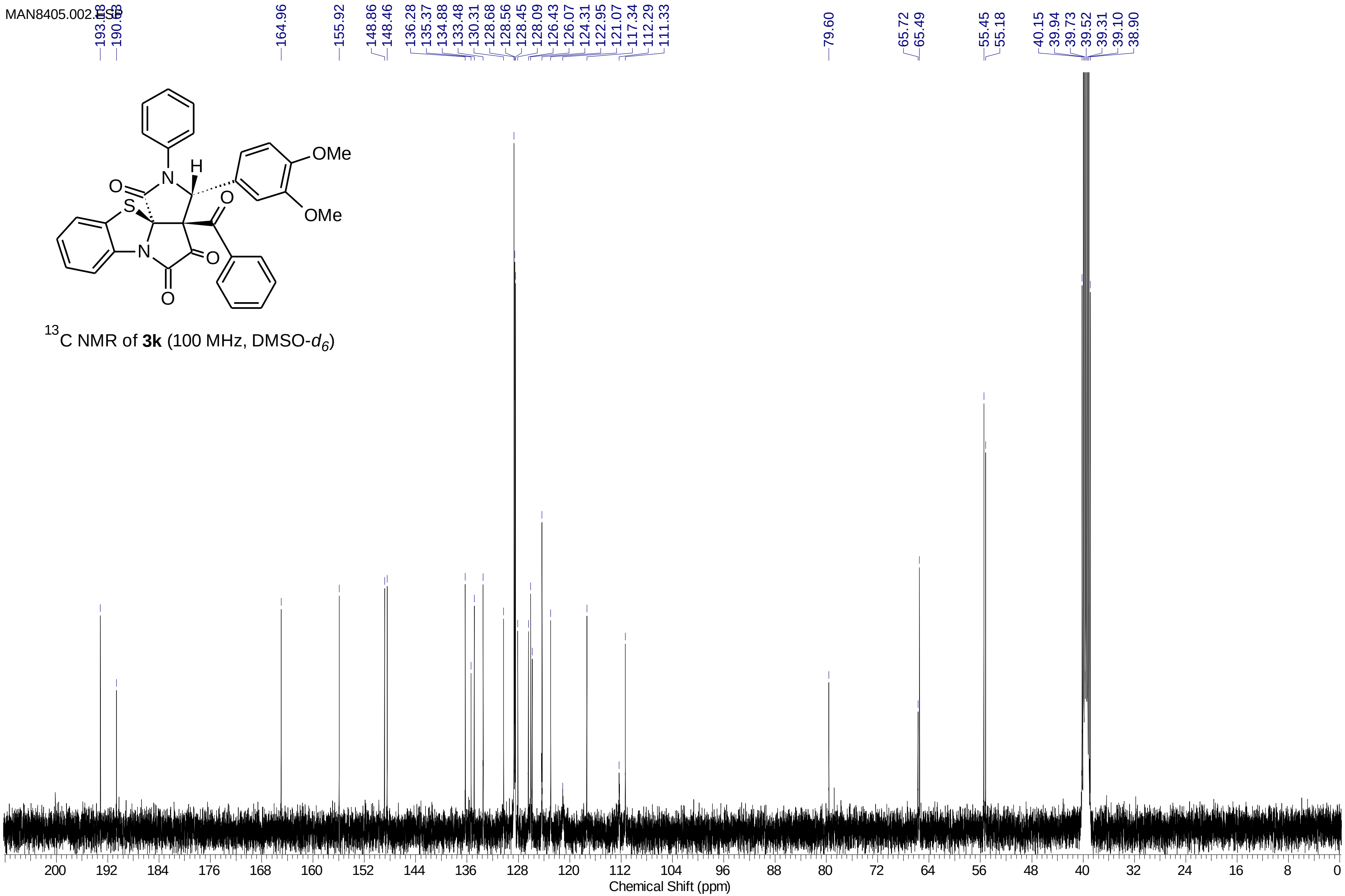


$^{13}$C NMR of **3k** (100 MHz, DMSO-$d_6$)

MAN8405.003.ESP

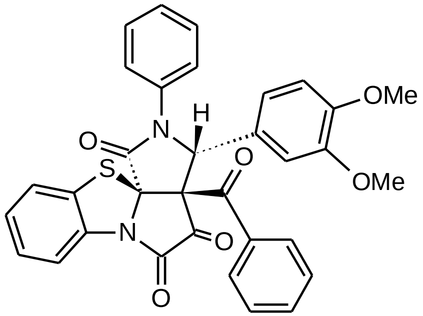

DEPT135 of **3k** (100 MHz, DMSO-$d_6$)

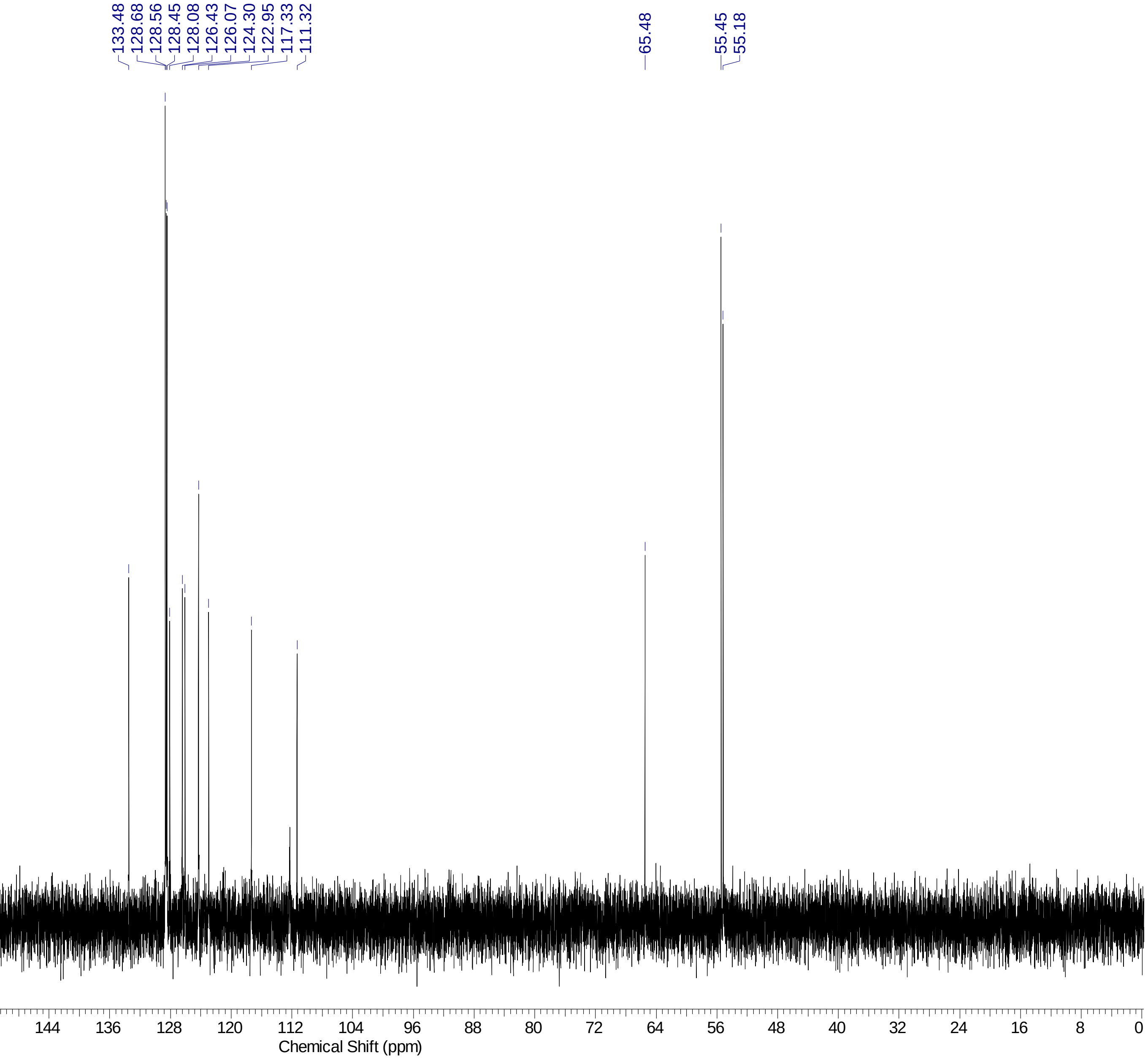

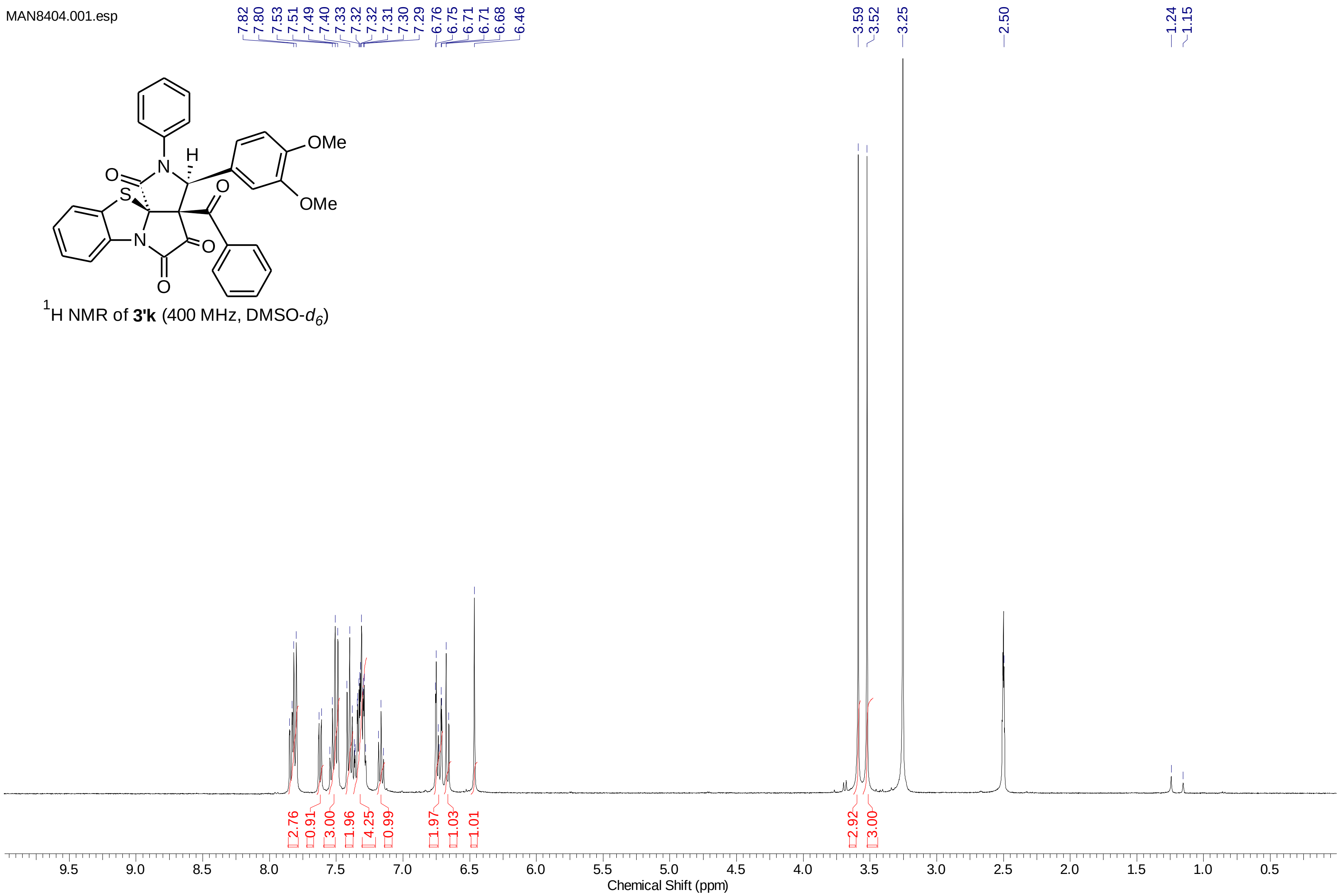


$^{1}$H NMR of **3'k** (400 MHz, DMSO-$d_6$)

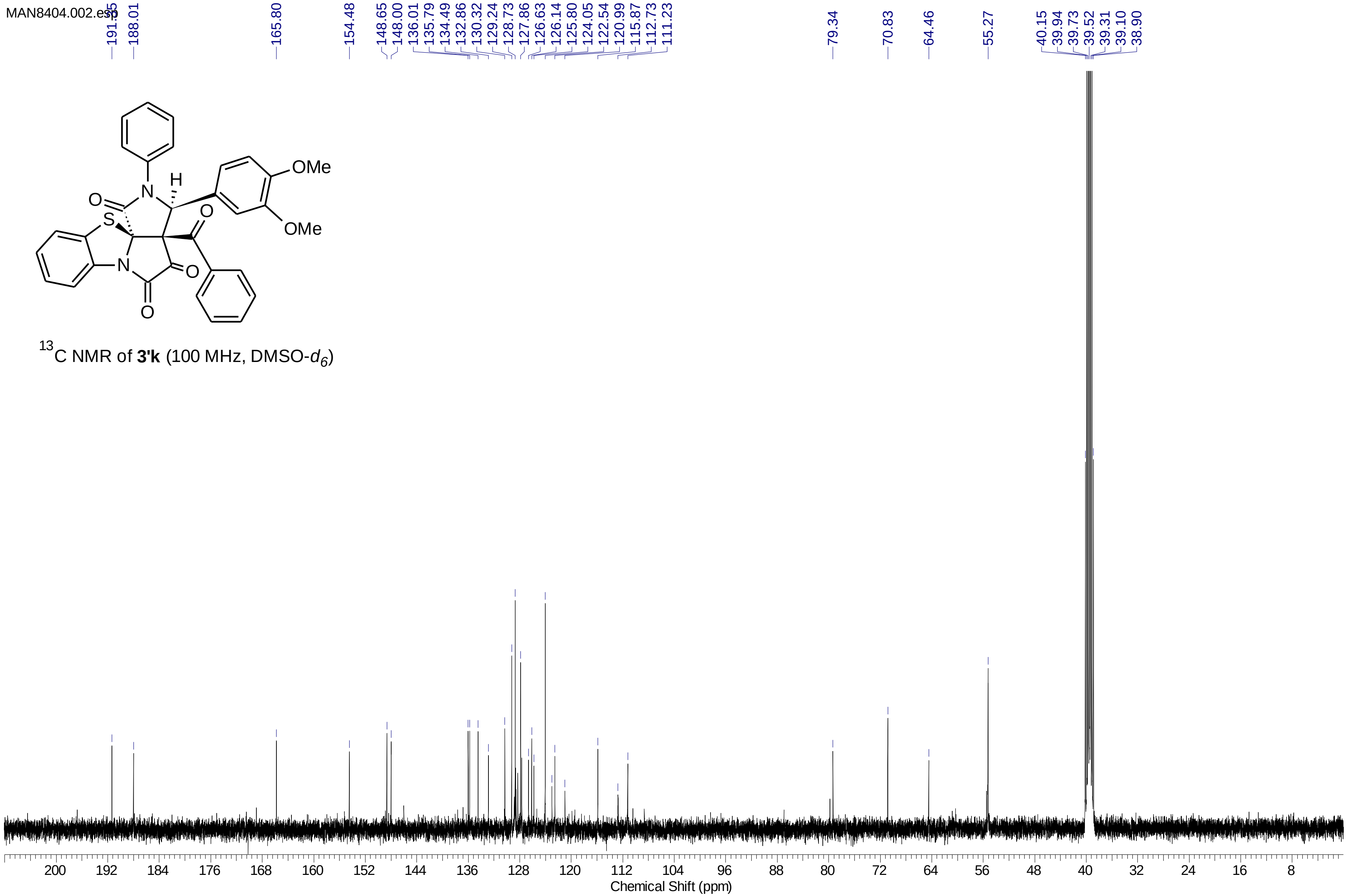


$^{13}$C NMR of **3'k** (100 MHz, DMSO-$d_6$)

MAN8404.003.esp

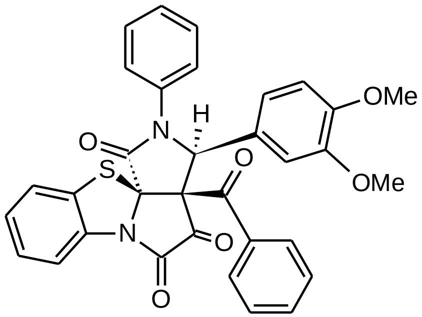

DEPT135 of **3k** (100 MHz, DMSO-$d_6$)

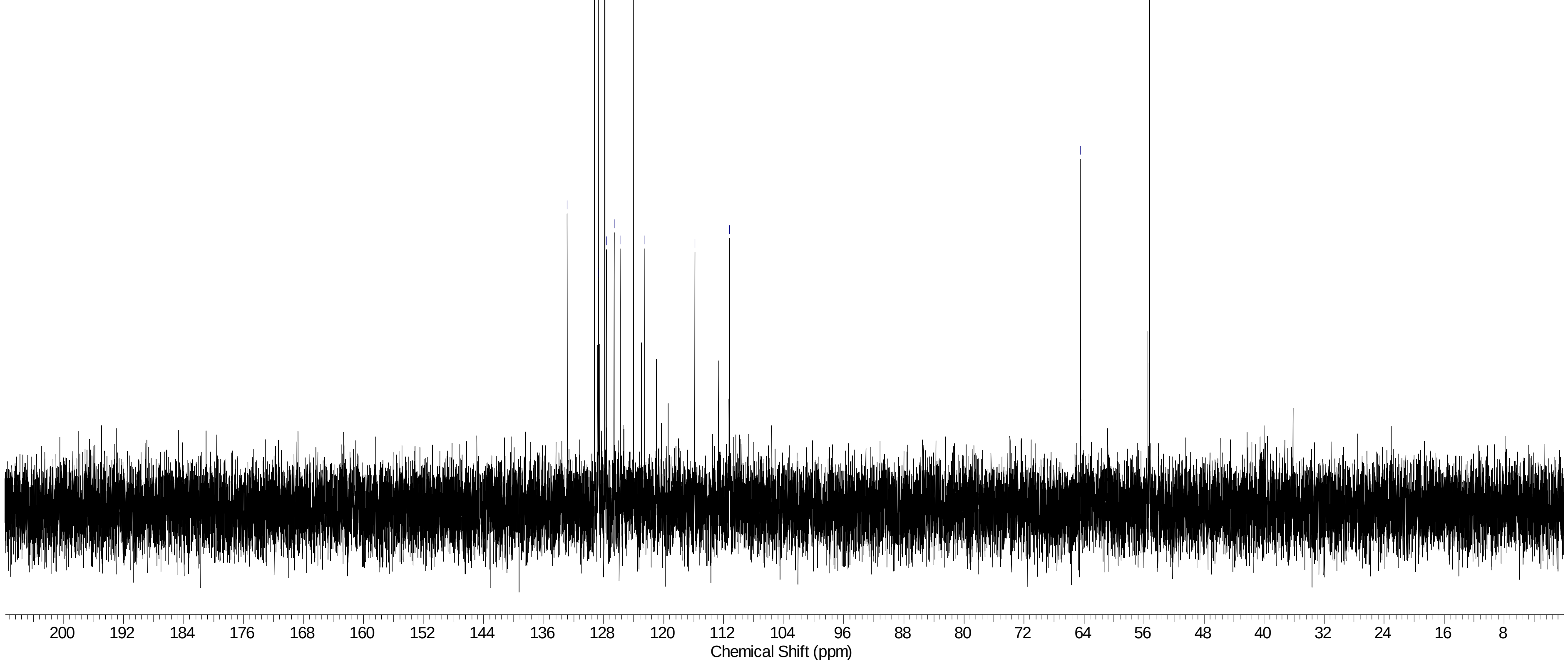

MAN8596.005.ESP

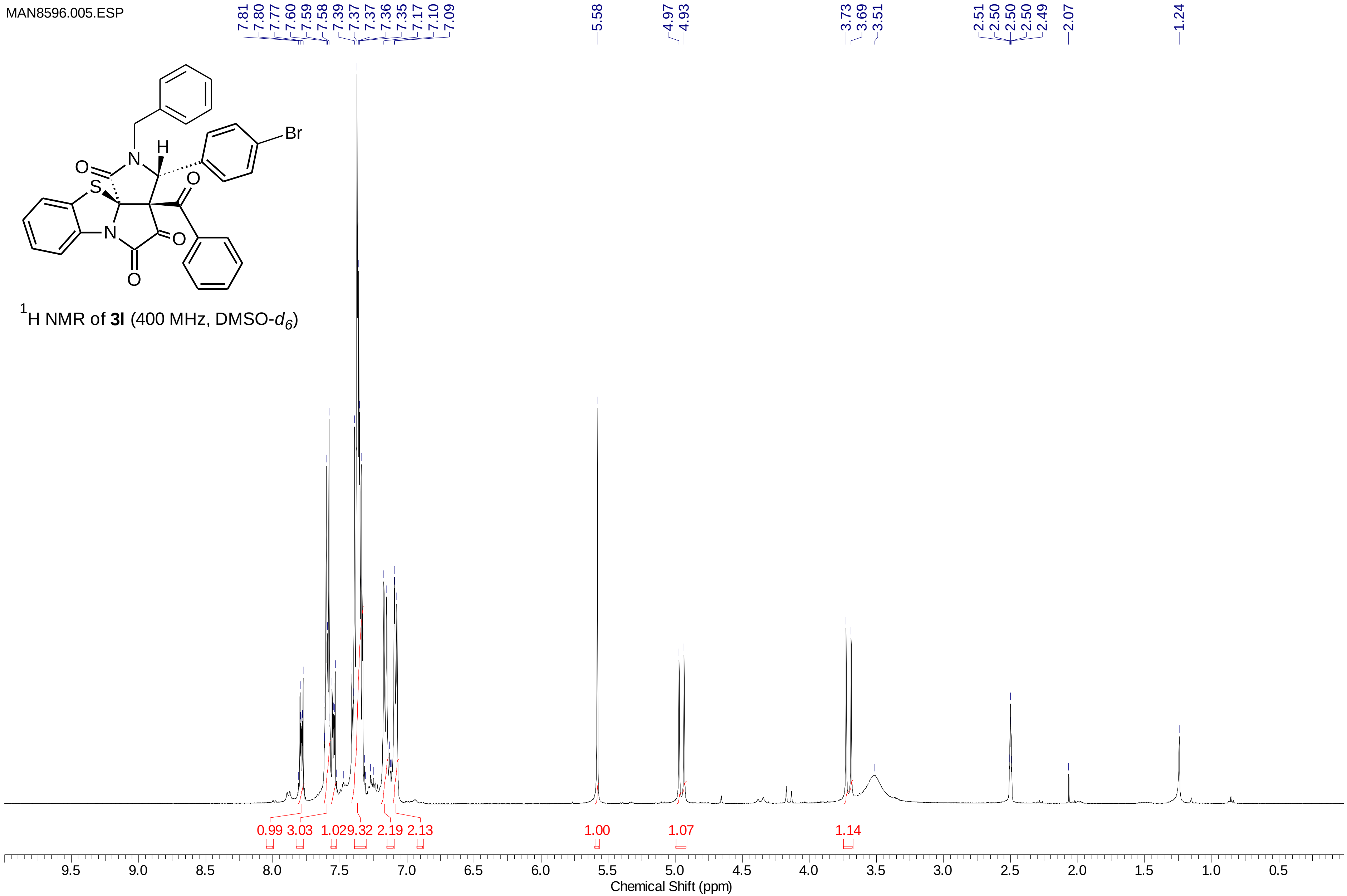


$^{1}$H NMR of **3l** (400 MHz, DMSO-$d_6$)

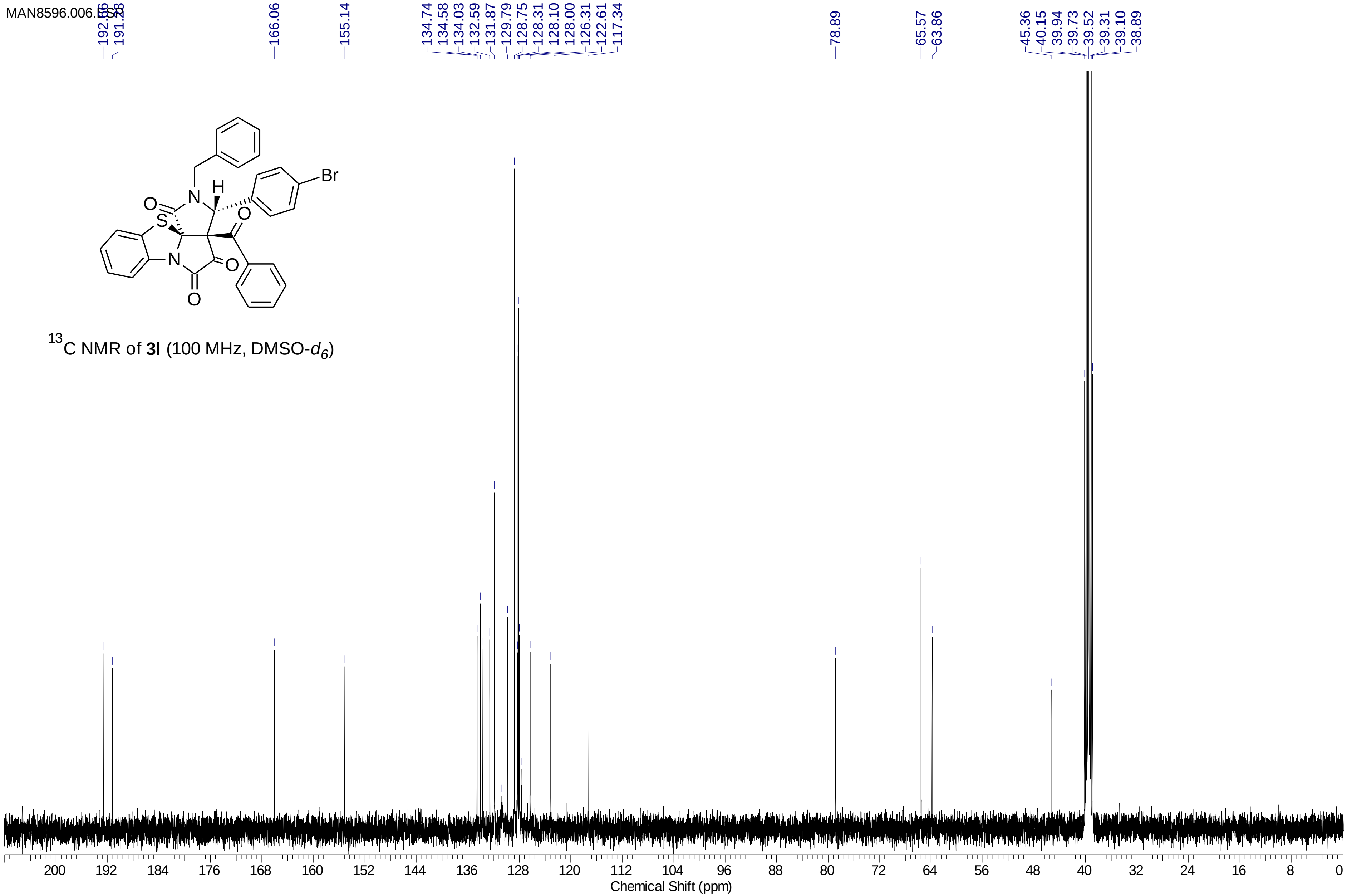


$^{13}$C NMR of **3l** (100 MHz, DMSO-$d_6$)

MAN8596.007.ESP

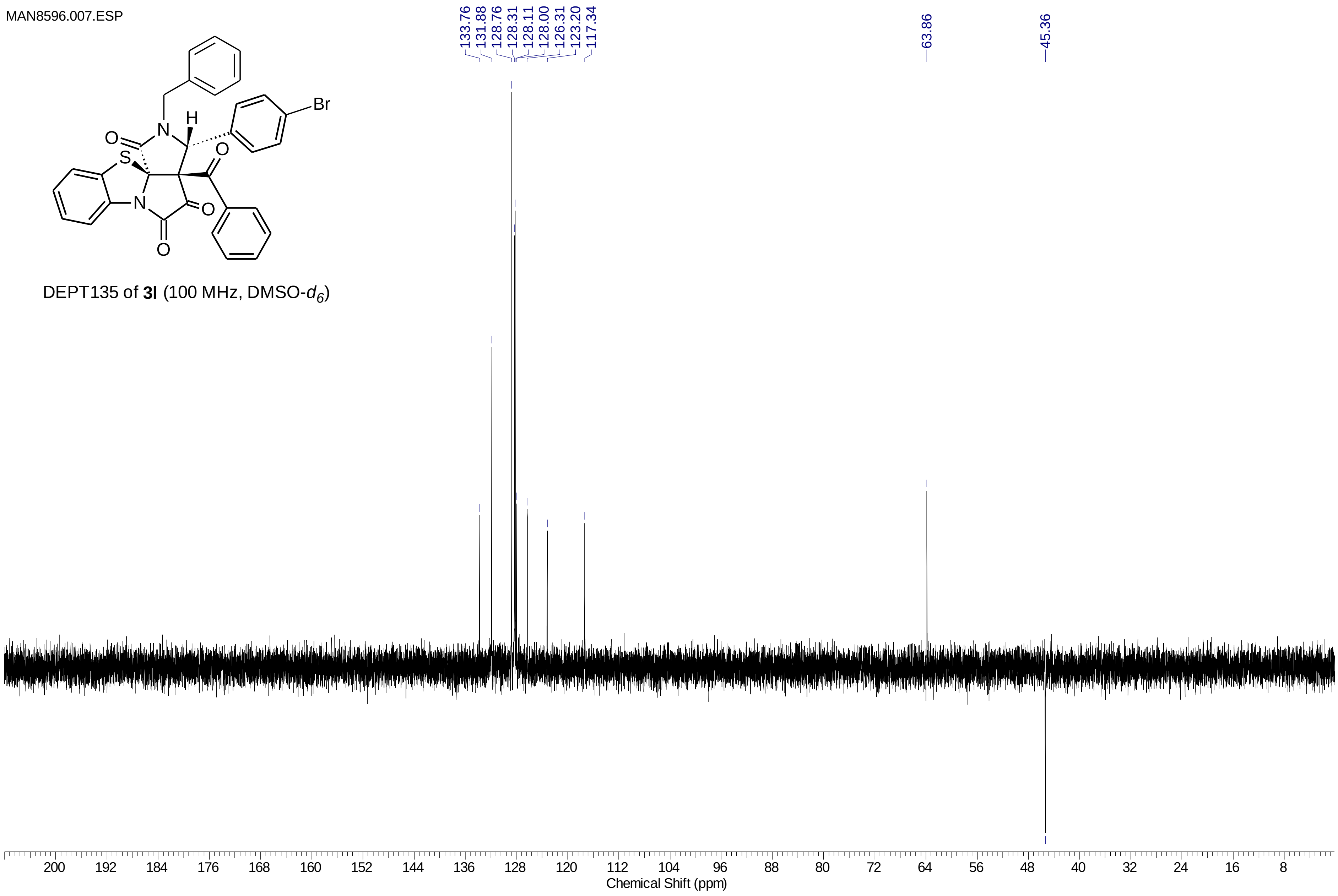


DEPT135 of **3l** (100 MHz, DMSO-$d_6$)

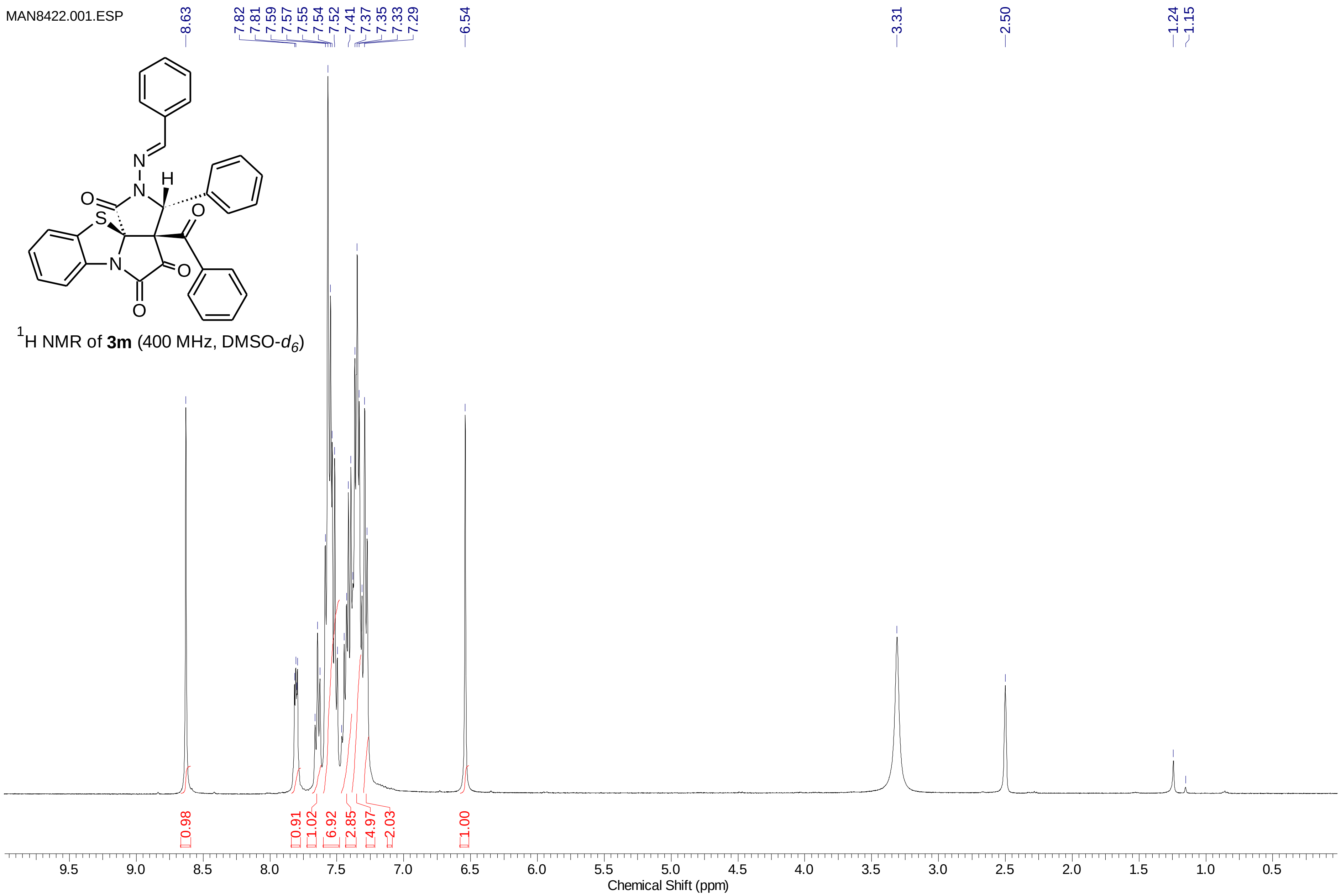


$^1$H NMR of **3m** (400 MHz, DMSO-$d_6$)

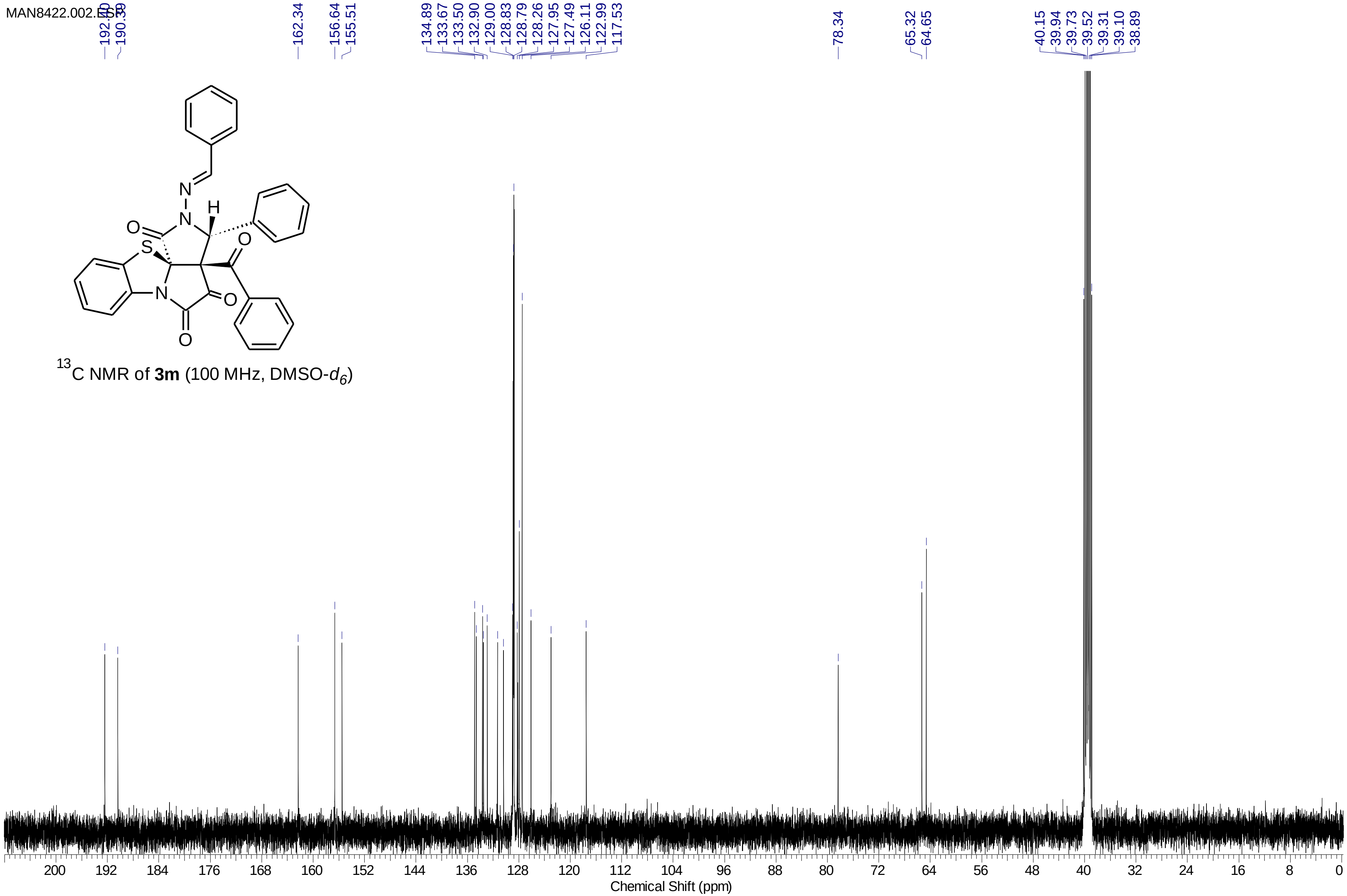


$^{13}$C NMR of **3m** (100 MHz, DMSO-$d_6$)

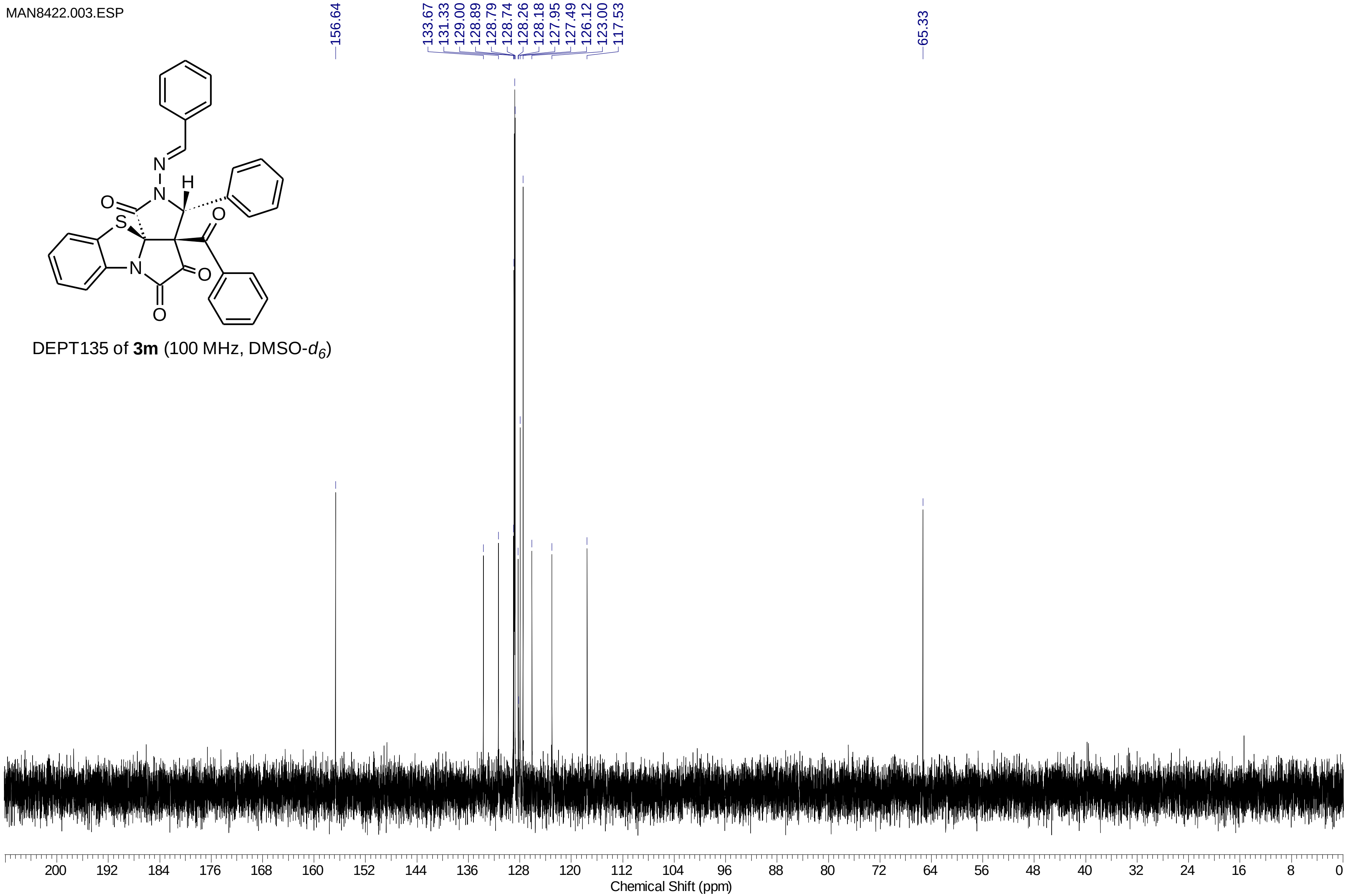


DEPT135 of **3m** (100 MHz, DMSO-$d_6$)

MAN7116.004.ESP

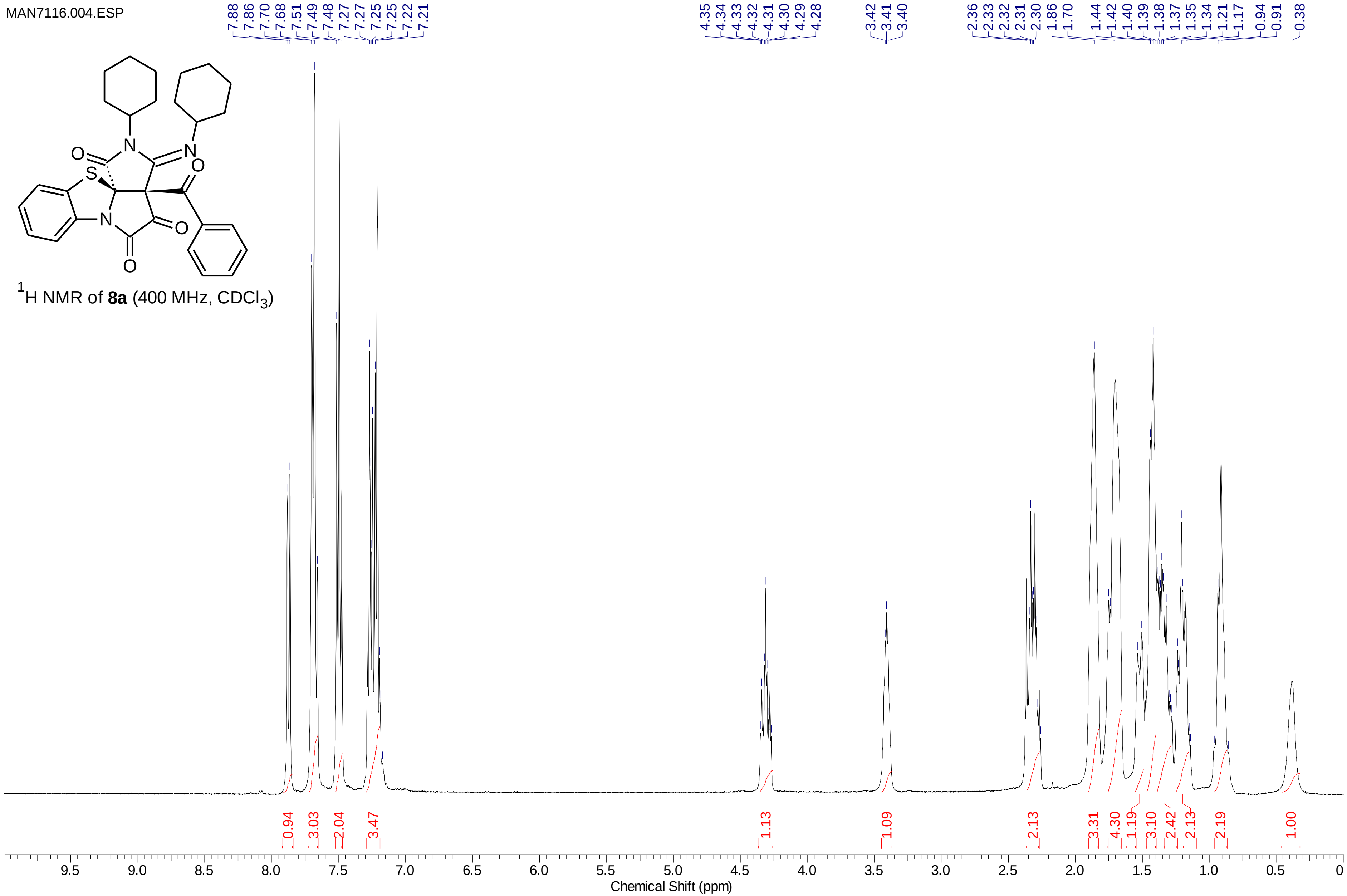


$^{1}$H NMR of **8a** (400 MHz, $CDCl_3$)

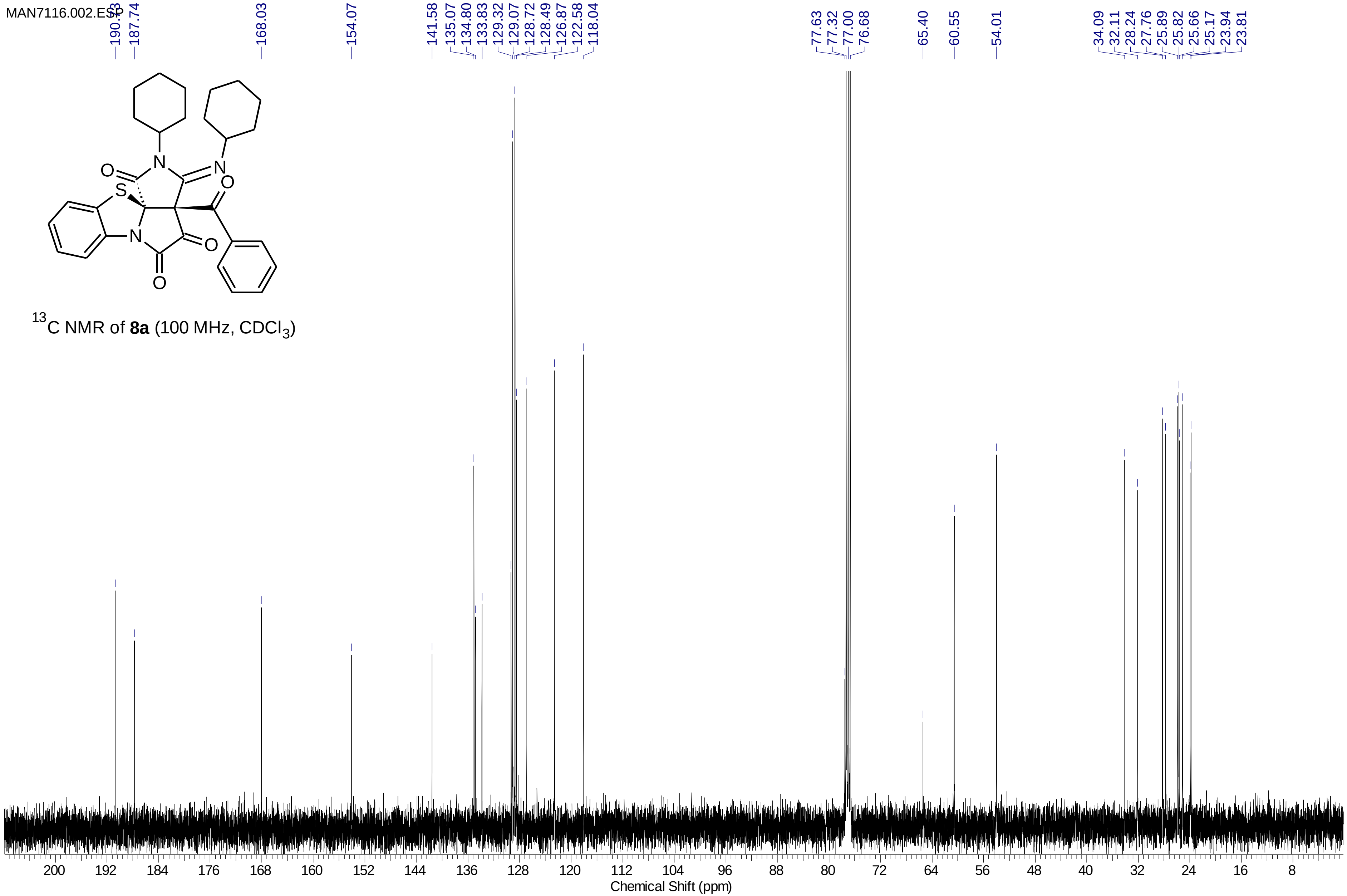


$^{13}C$ NMR of **8a** (100 MHz, $CDCl_3$)

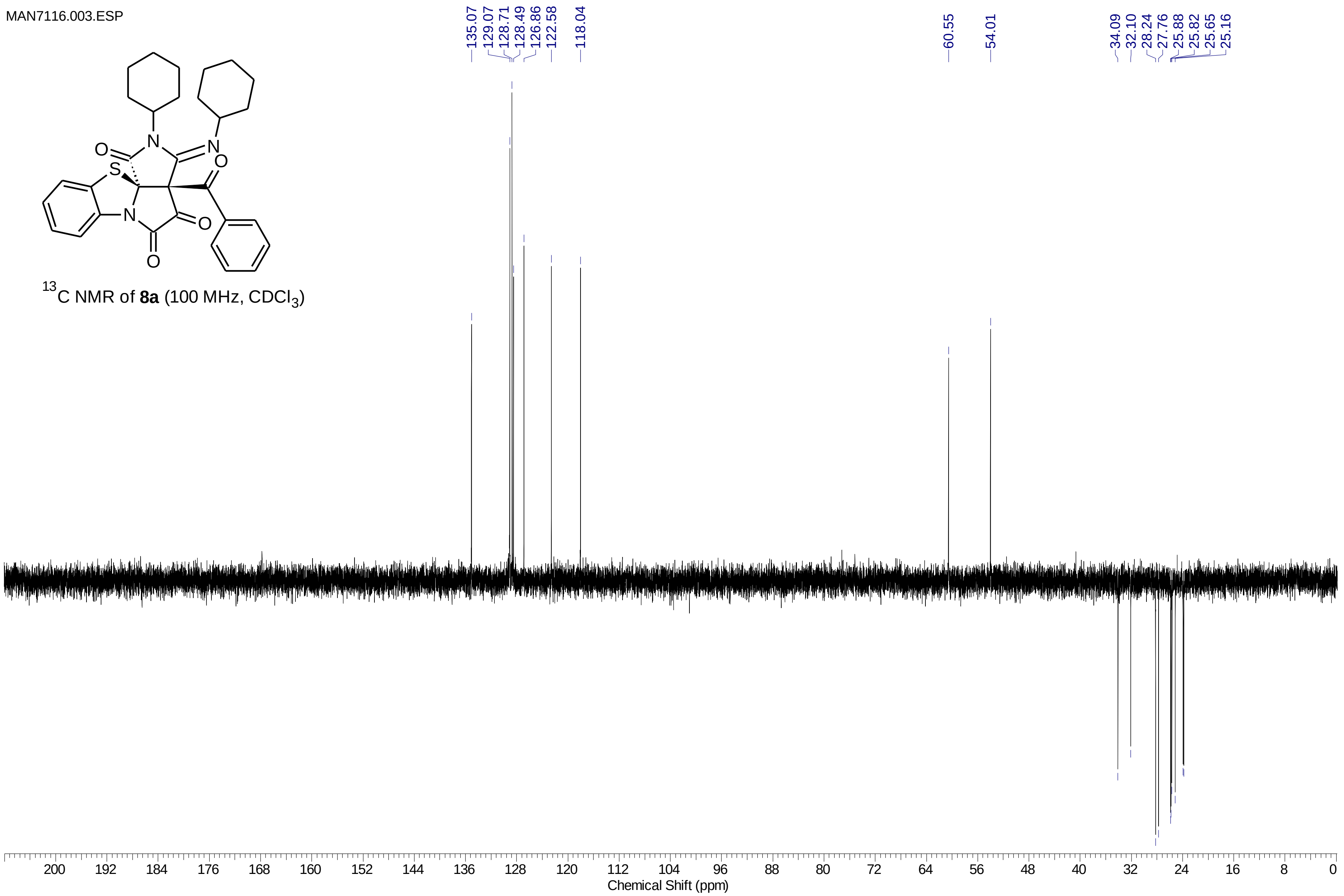
MAN7116.003.ESP
135.07
129.07
128.71
128.49
126.86
122.58
118.04
60.55
54.01
34.09
32.10
28.24
27.76
25.88
25.82
25.65
25.16
O
N
N
O
S
N
O
O
13C NMR of 8a (100 MHz, CDCl3)
200 192 184 176 168 160 152 144 136 128 120 112 104 96 88 80 72 64 56 48 40 32 24 16 8 0
Chemical Shift (ppm)

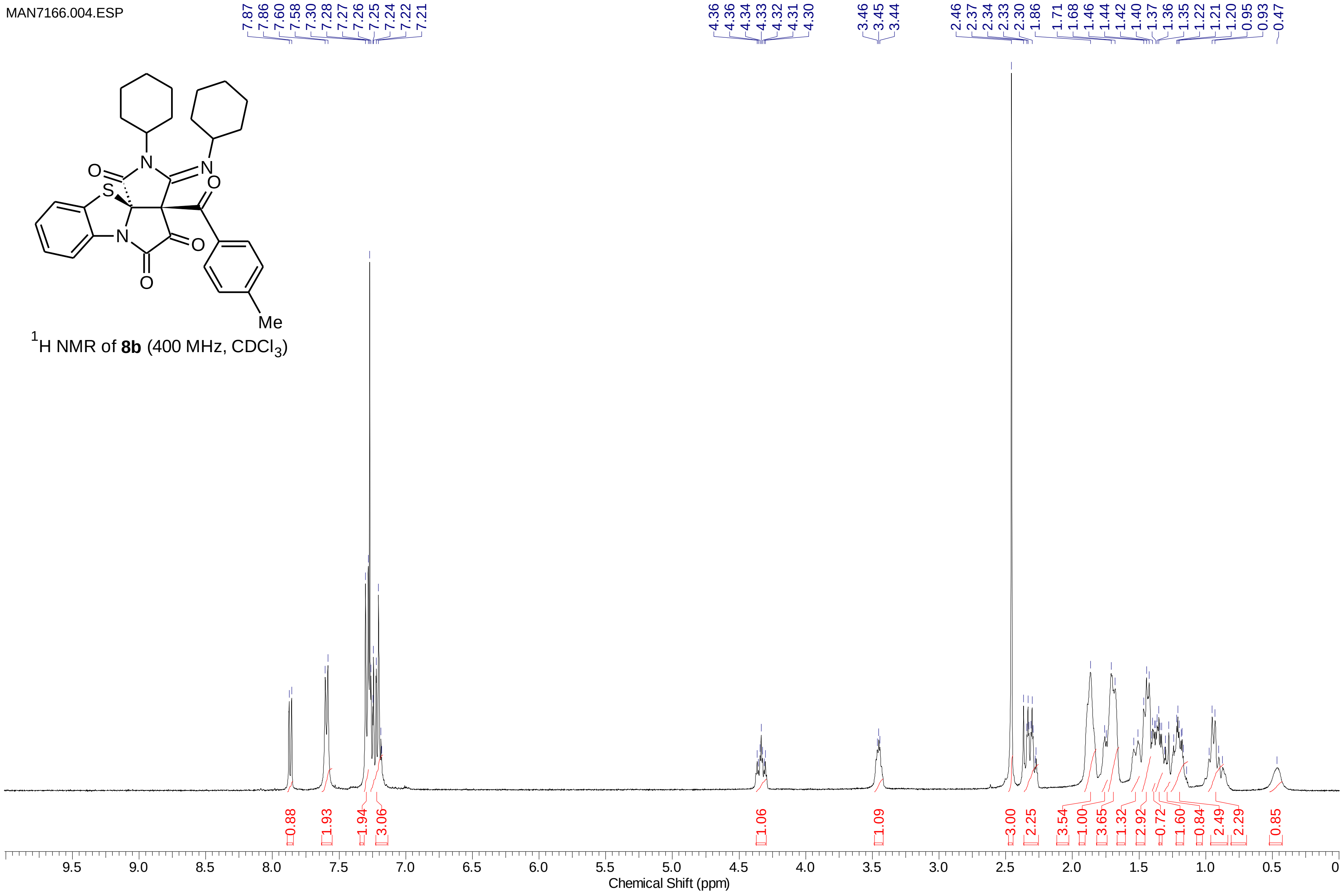

$^1$H NMR of **8b** (400 MHz, $CDCl_3$)

MAN7166.002.ESP

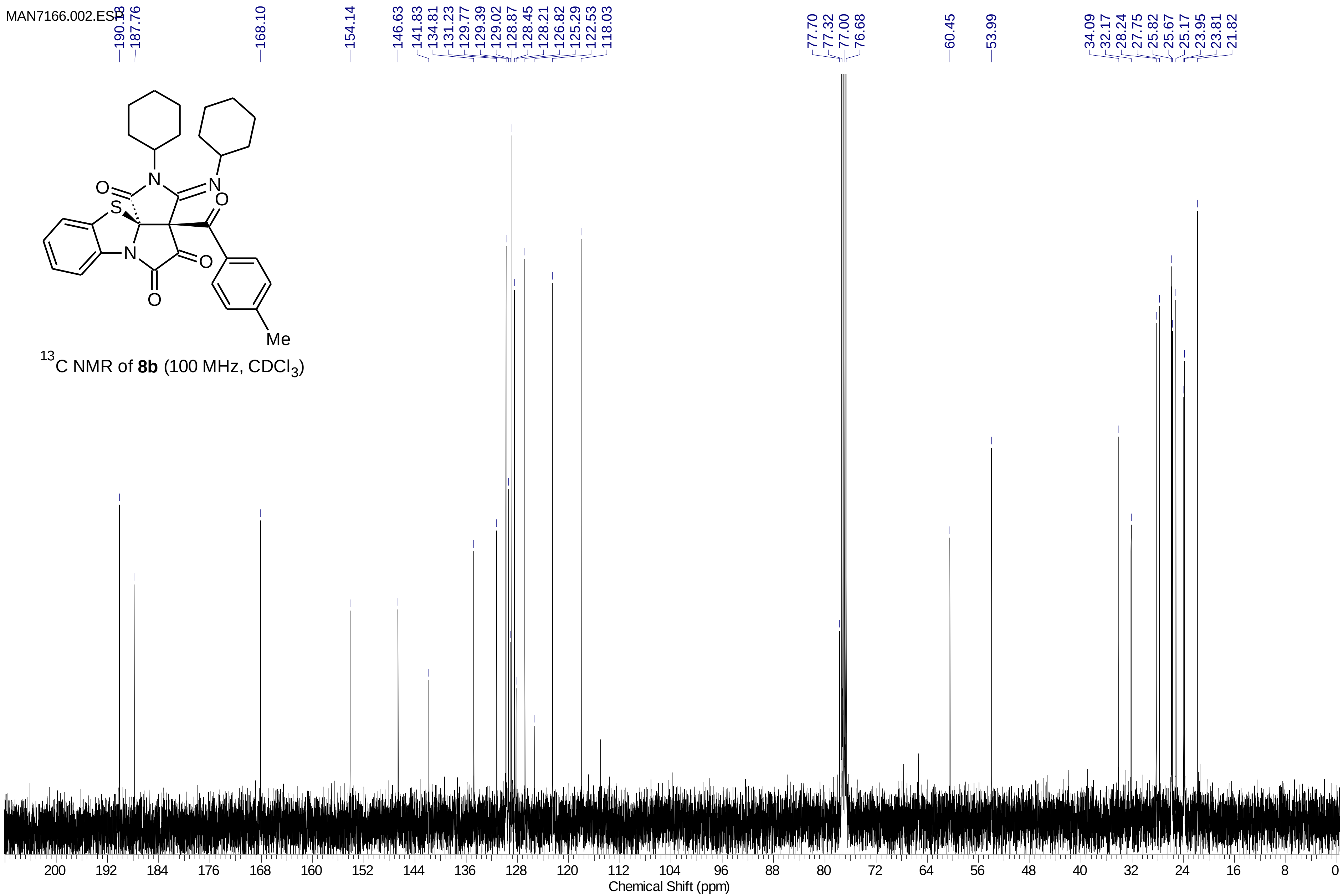


$^{13}$C NMR of **8b** (100 MHz, $CDCl_3$)

MAN7166.003.ESP

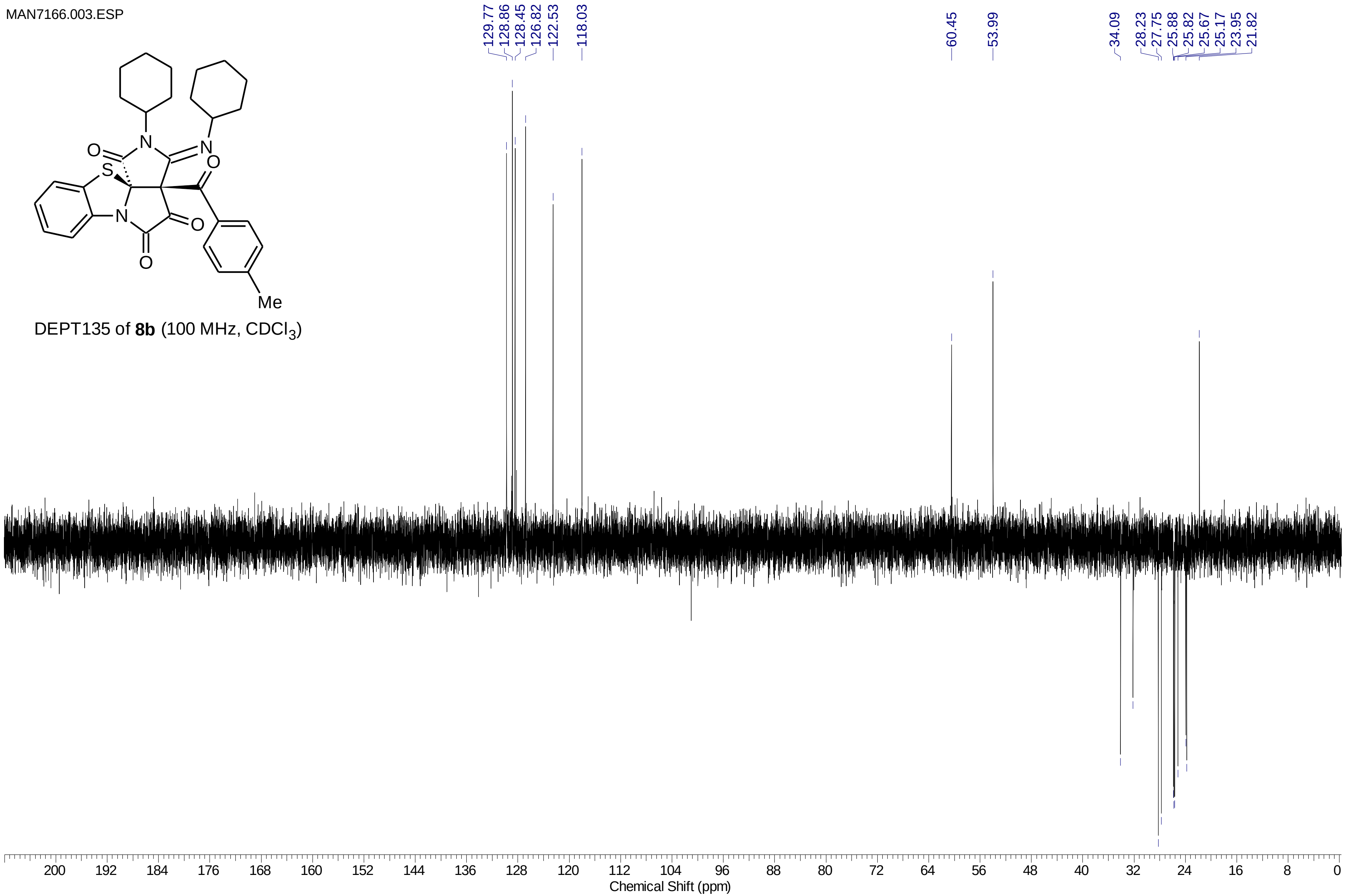


DEPT135 of **8b** (100 MHz, $CDCl_3$)

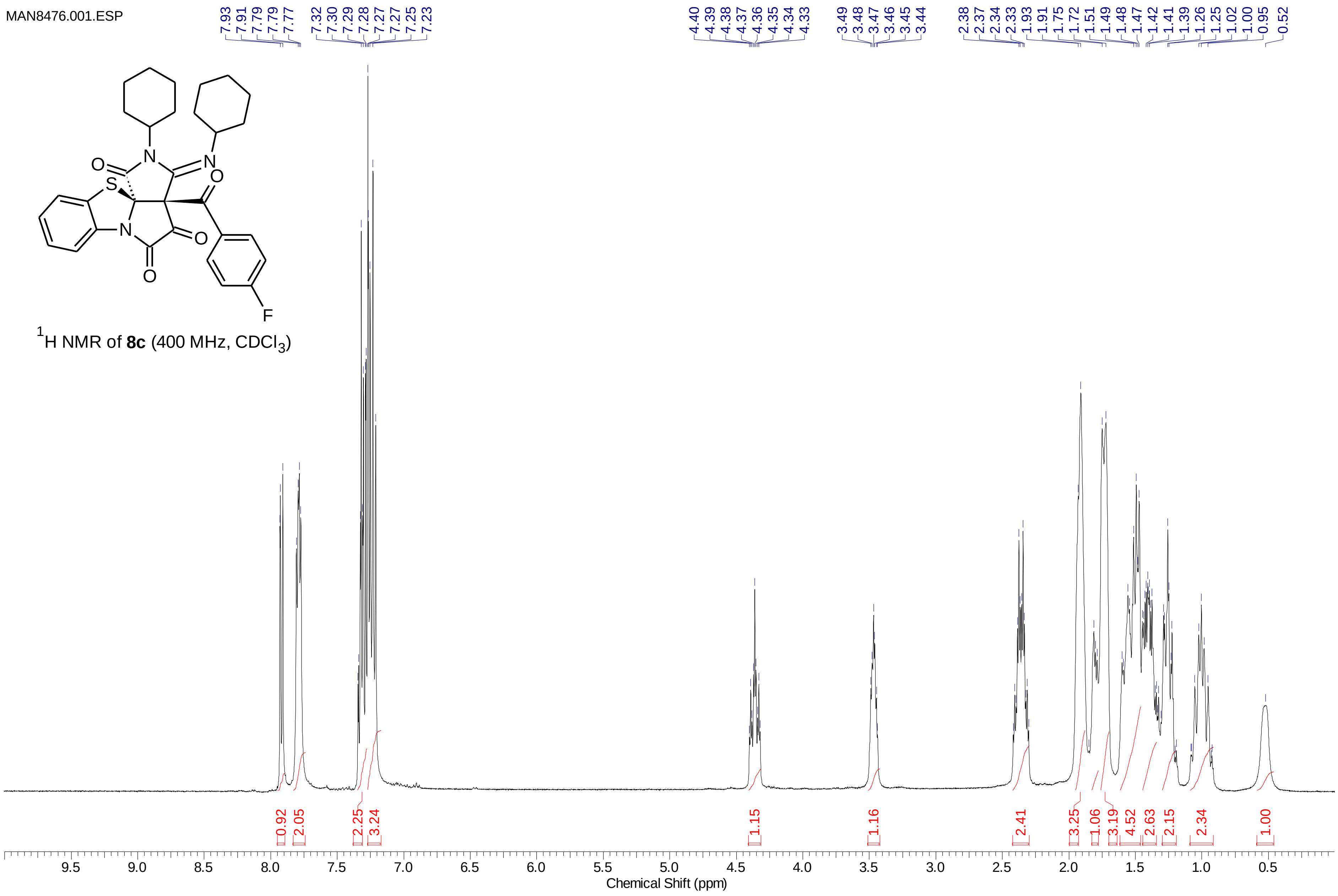


$^{1}$H NMR of **8c** (400 MHz, $CDCl_3$)

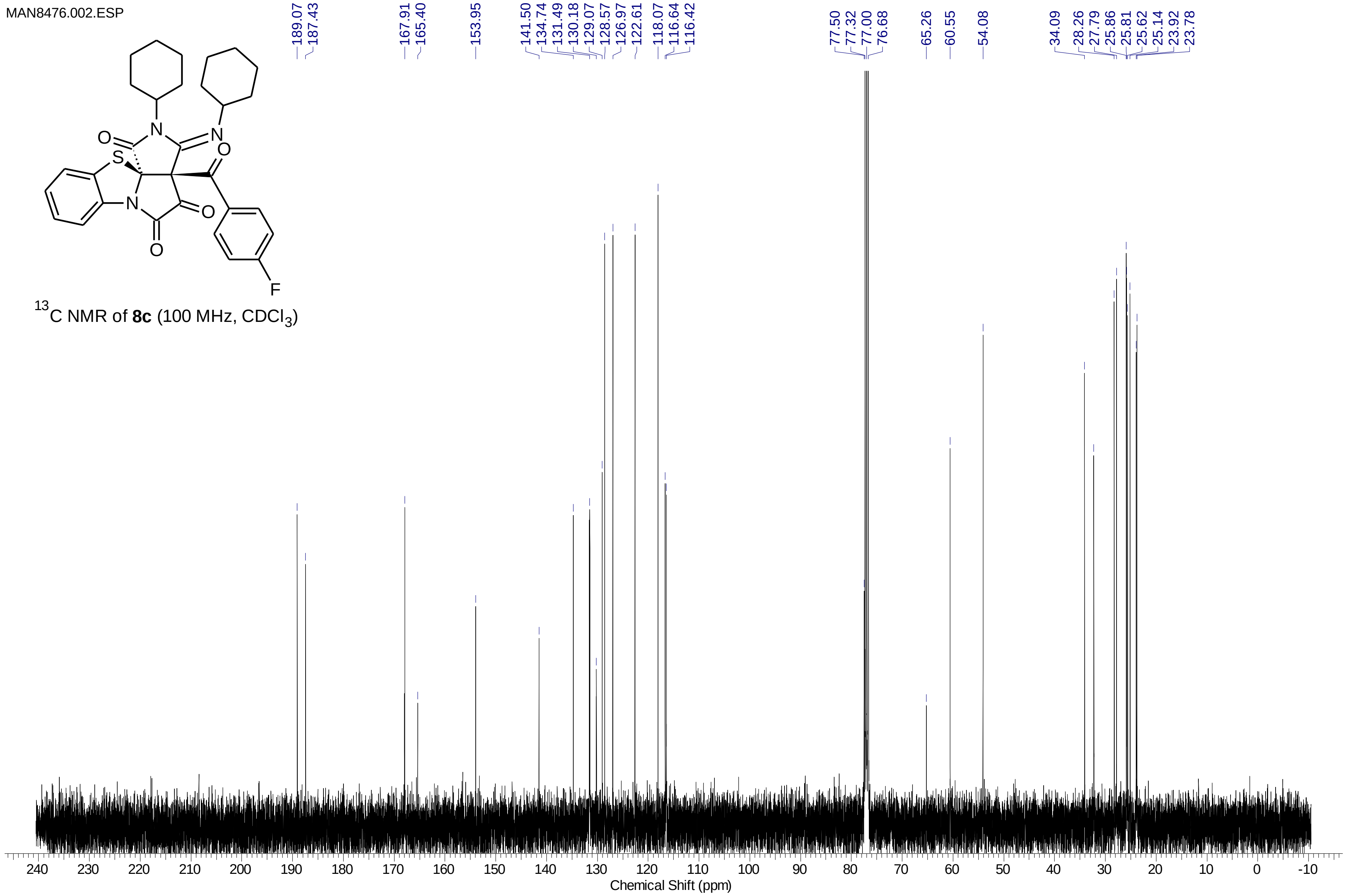


$^{13}C$ NMR of **8c** (100 MHz, $CDCl_3$)

MAN8476.003.ESP

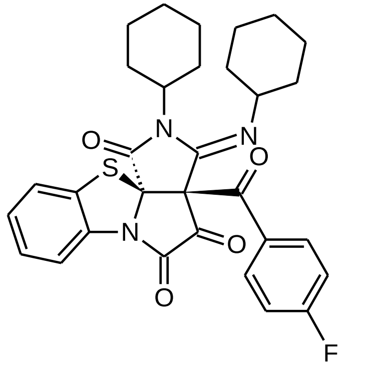


DEPT135 of **8c** (100 MHz, $CDCl_3$)

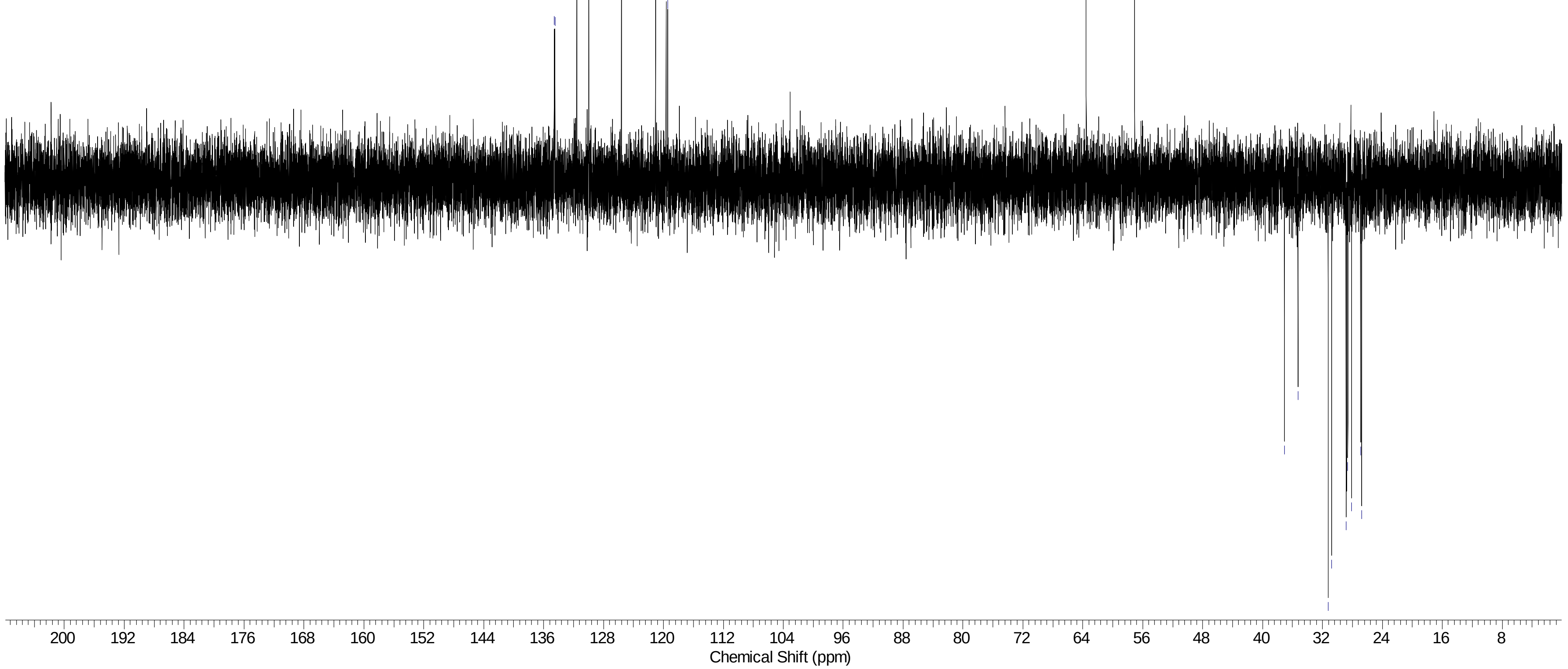

MAN8476.004.ESP

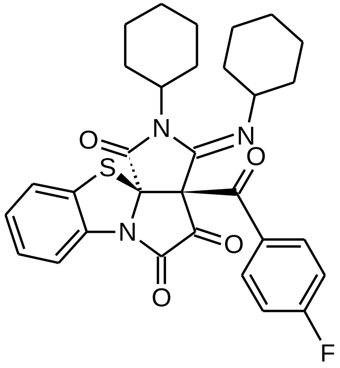


$^{19}$F NMR of **8c** (376 MHz, $CDCl_3$)

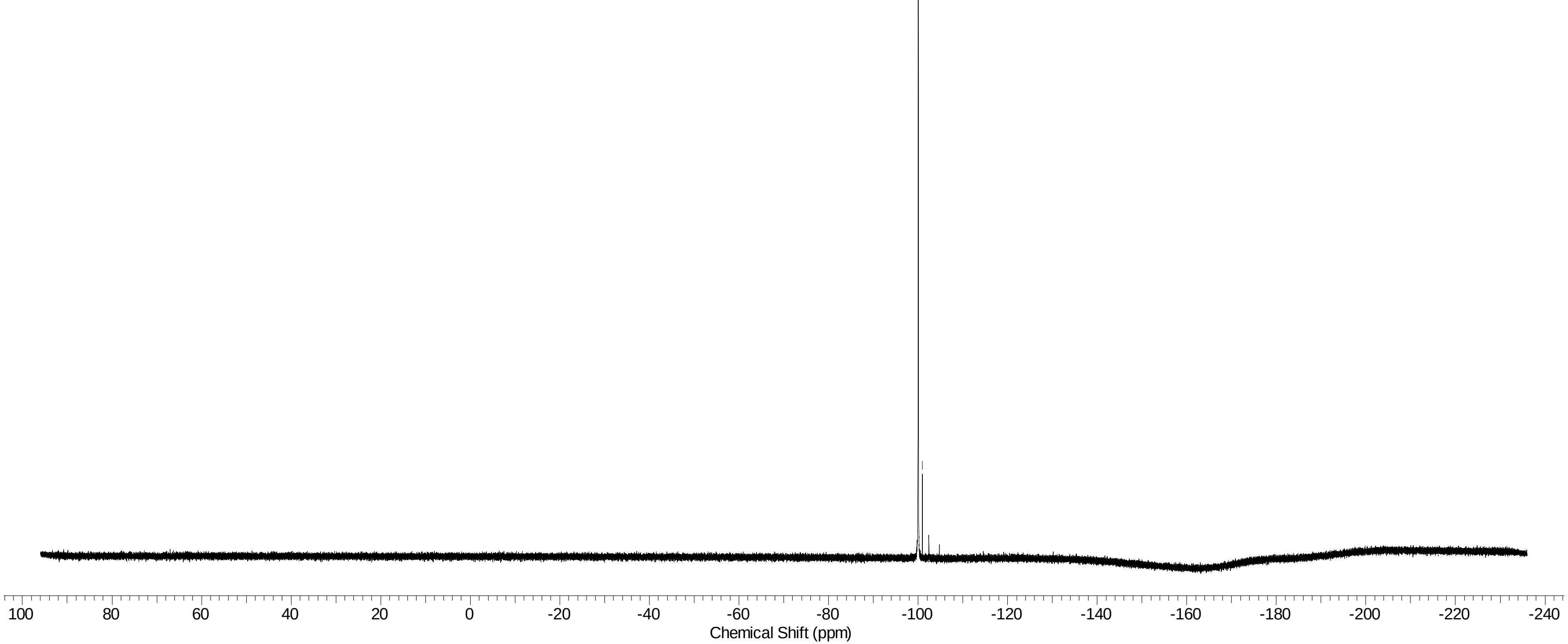

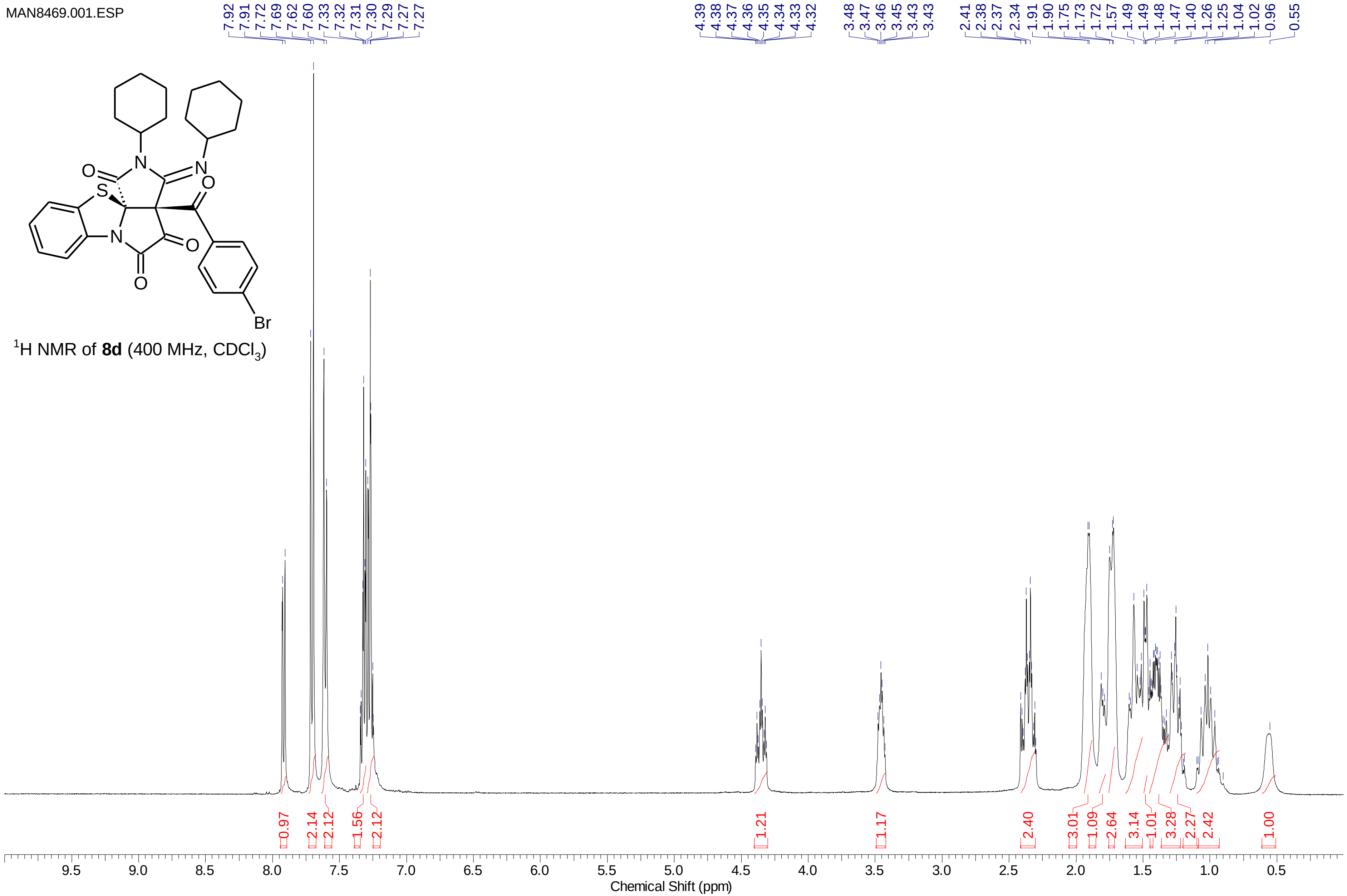


$^{1}$H NMR of **8d** (400 MHz, $CDCl_3$)

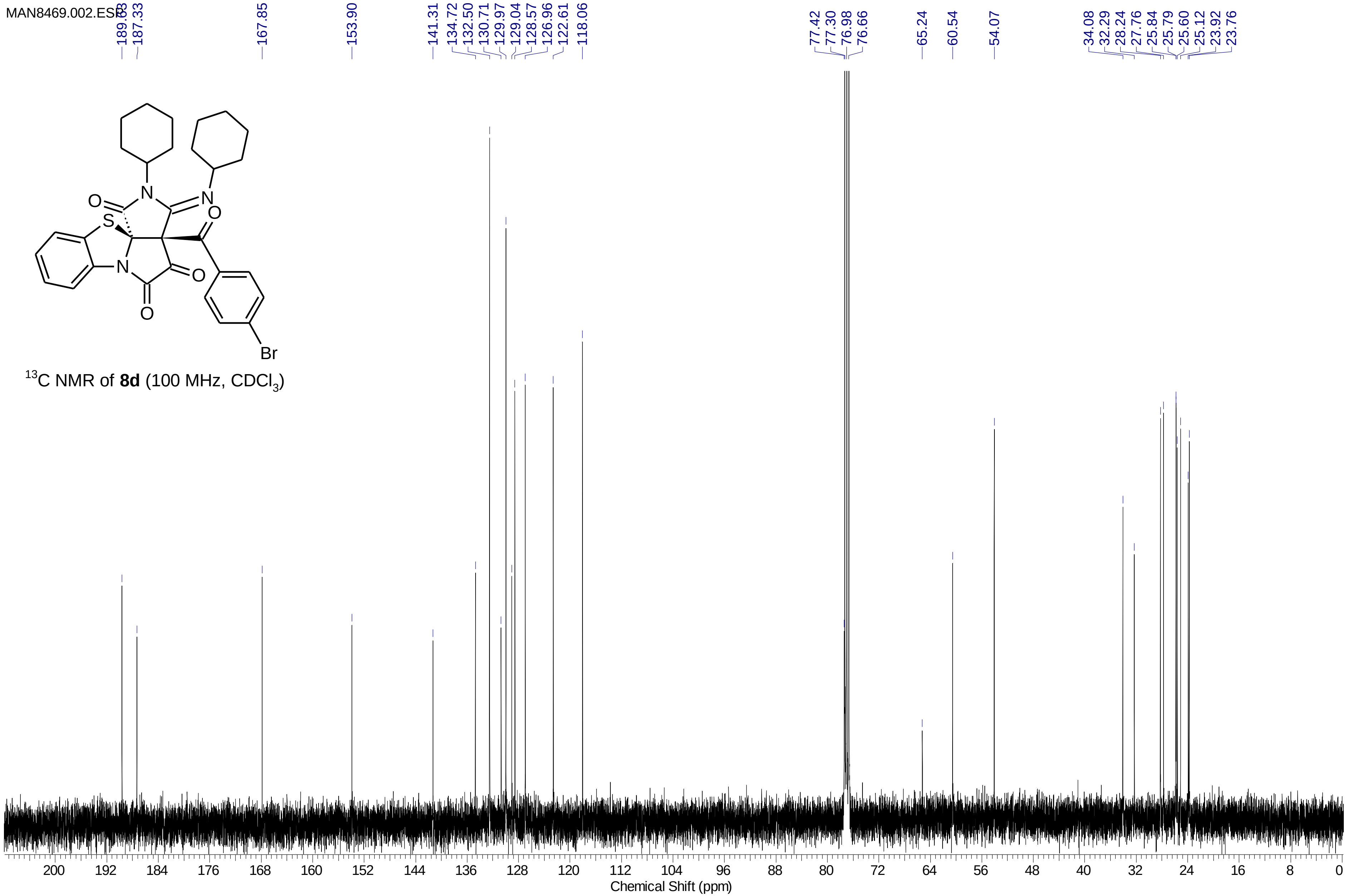


$^{13}C$ NMR of **8d** (100 MHz, $CDCl_3$)

MAN8469.003.ESP

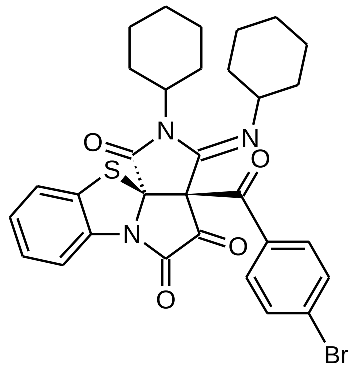

DEPT135 of **8d** (100 MHz, $CDCl_3$)

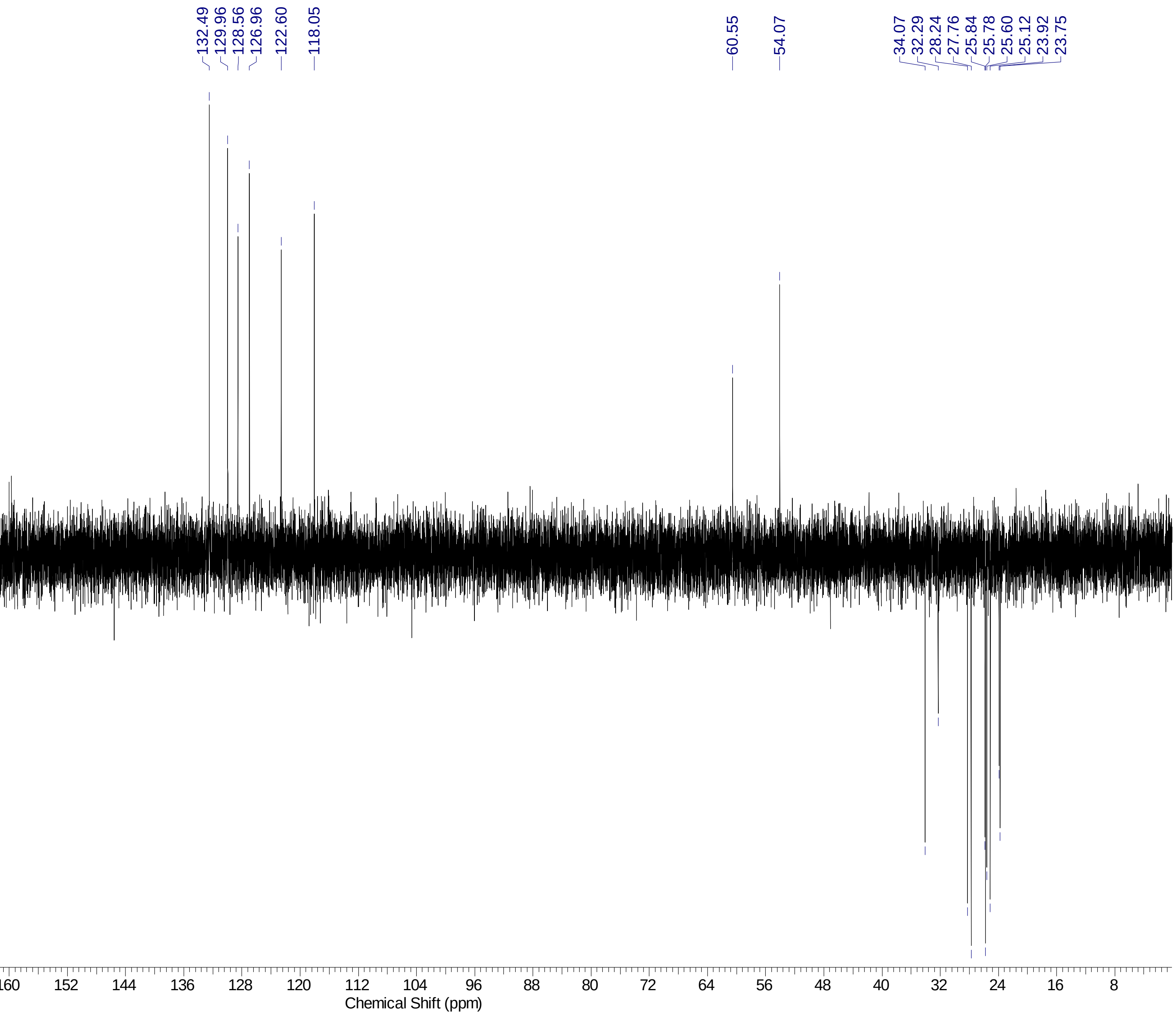

MAN8470.001.ESP

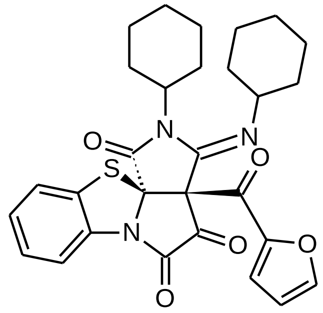


$^{1}$H NMR of **8e** (400 MHz, $CDCl_3$)

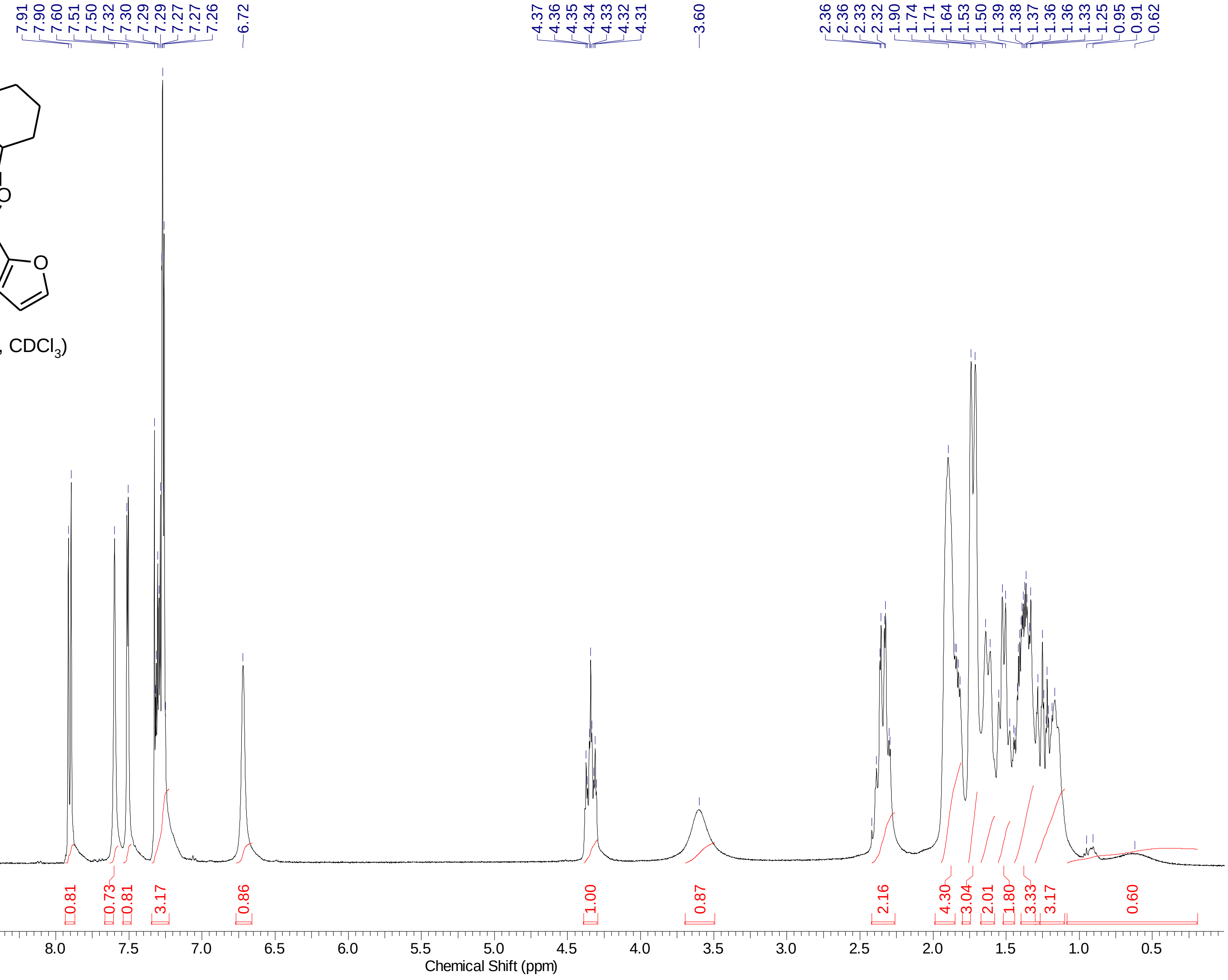

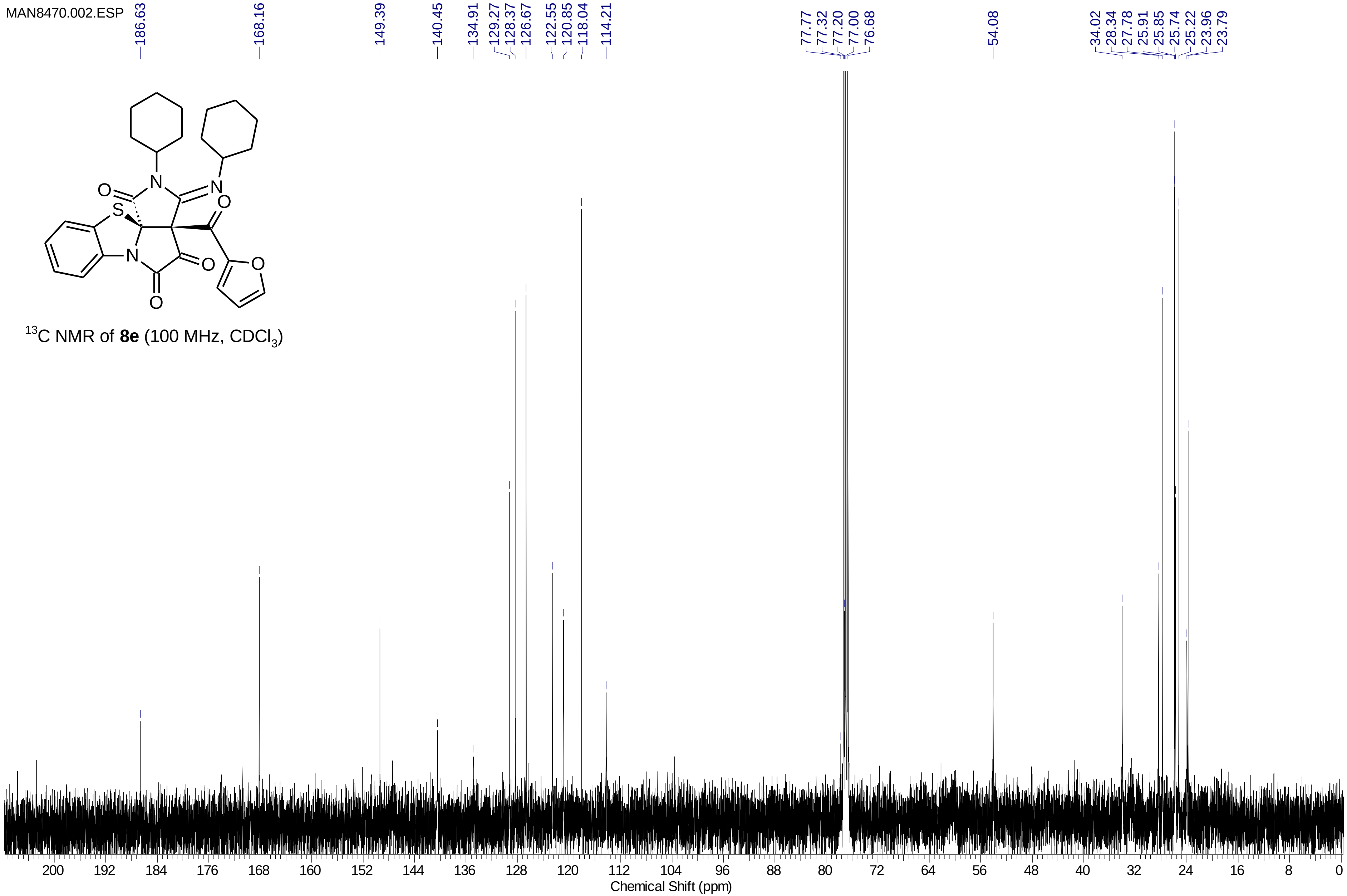


$^{13}$C NMR of **8e** (100 MHz, $CDCl_3$)

MAN8470.003.ESP

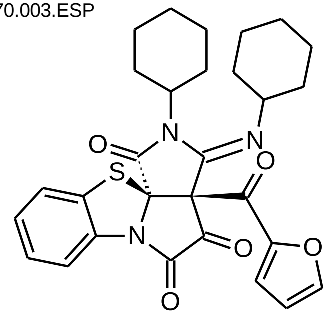

DEPT135 of **8e** (100 MHz, $CDCl_3$)

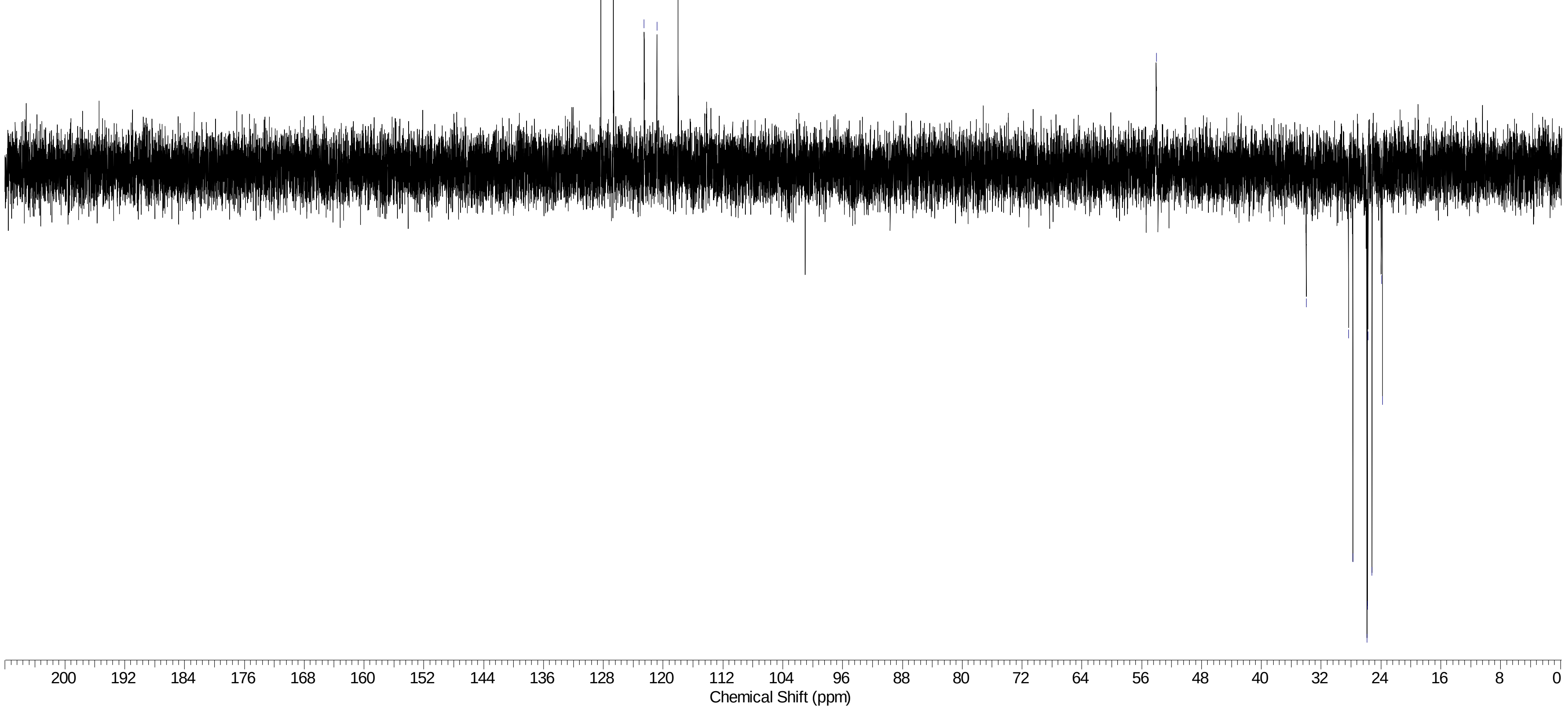

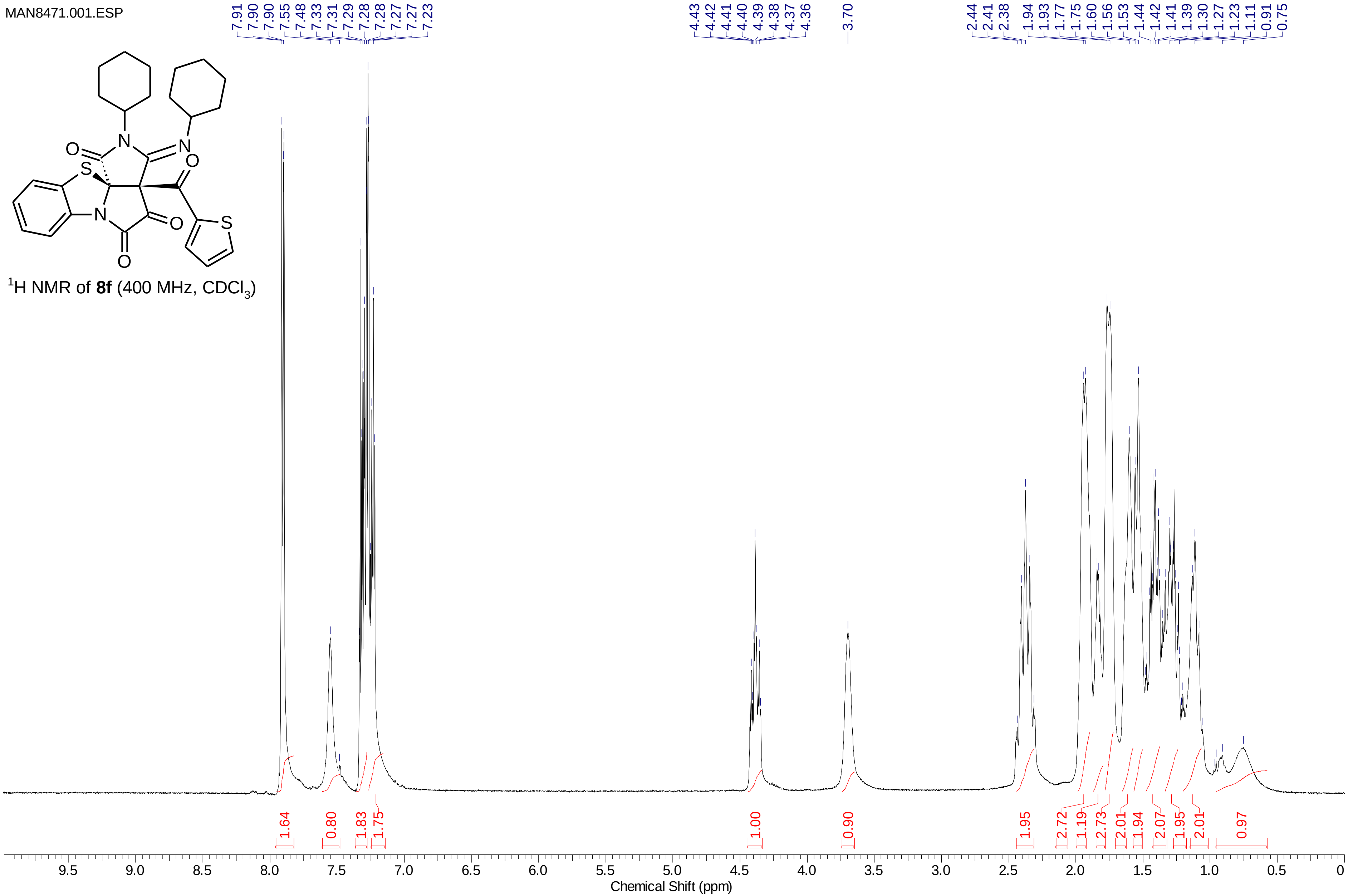


$^{1}H$ NMR of **8f** (400 MHz, $CDCl_3$)

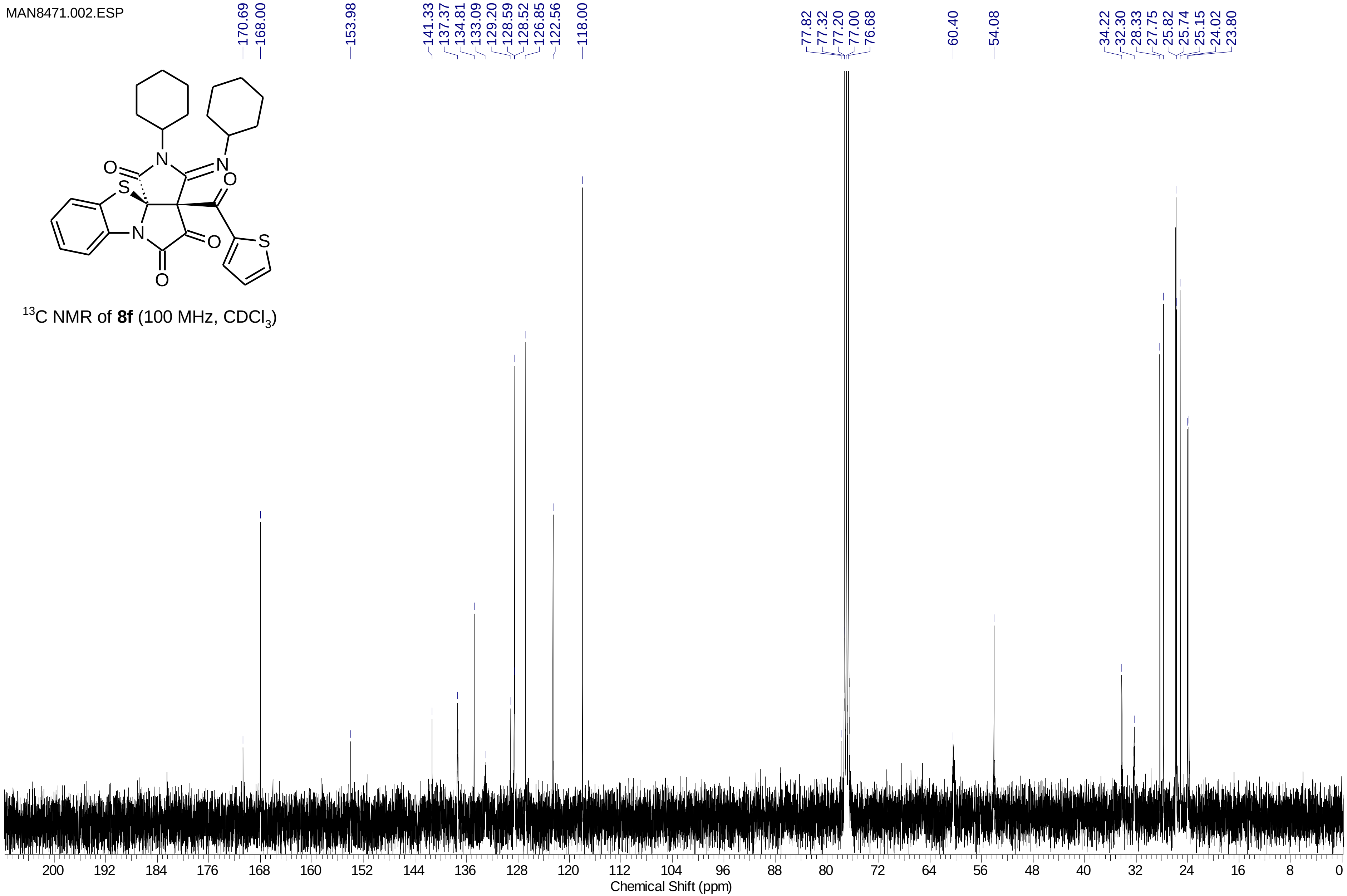


$^{13}$C NMR of **8f** (100 MHz, $CDCl_3$)

MAN8471.003.ESP

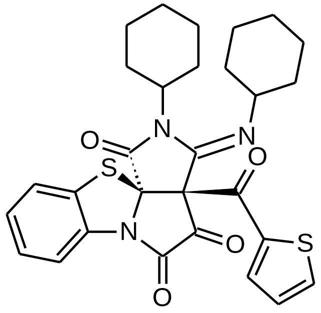

DEPT135 of **8f** (100 MHz, $CDCl_3$)

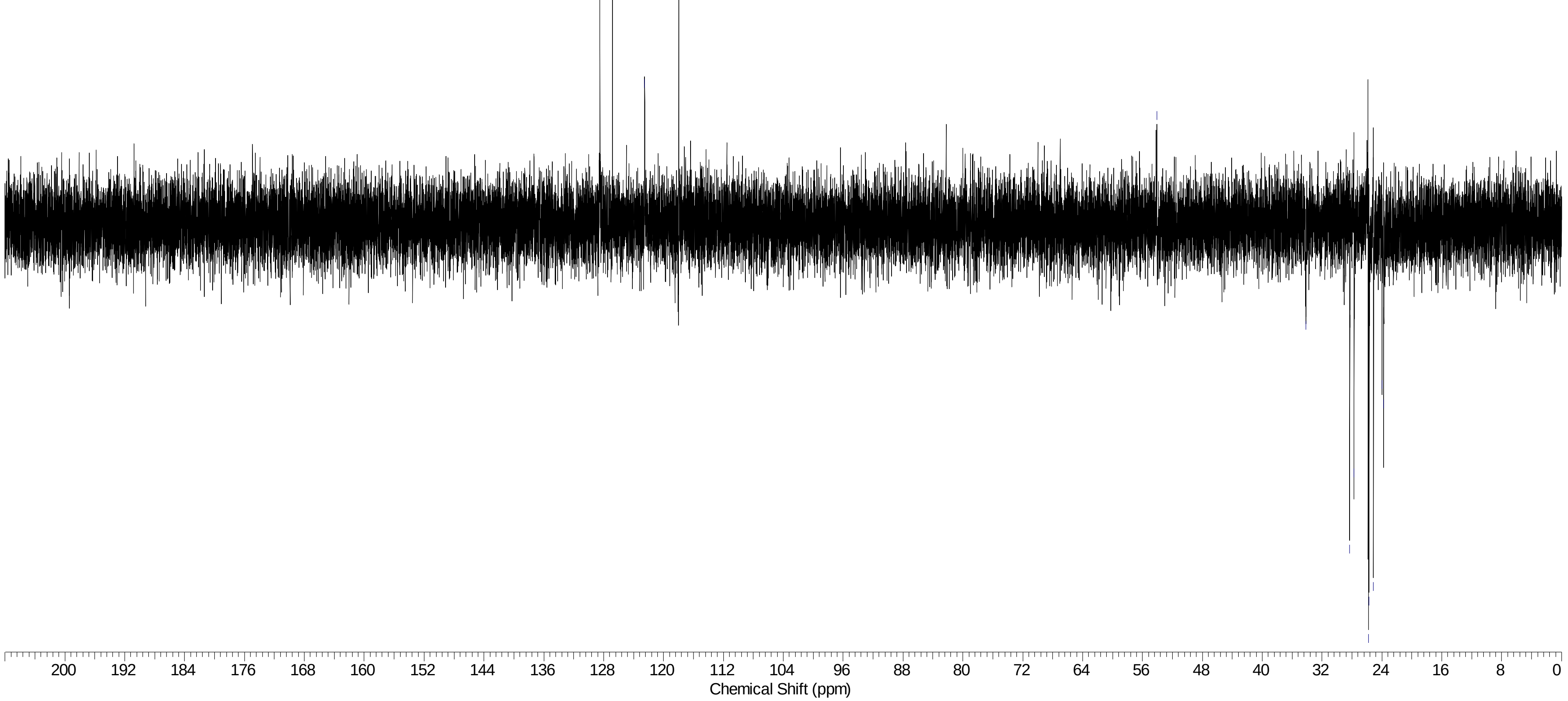

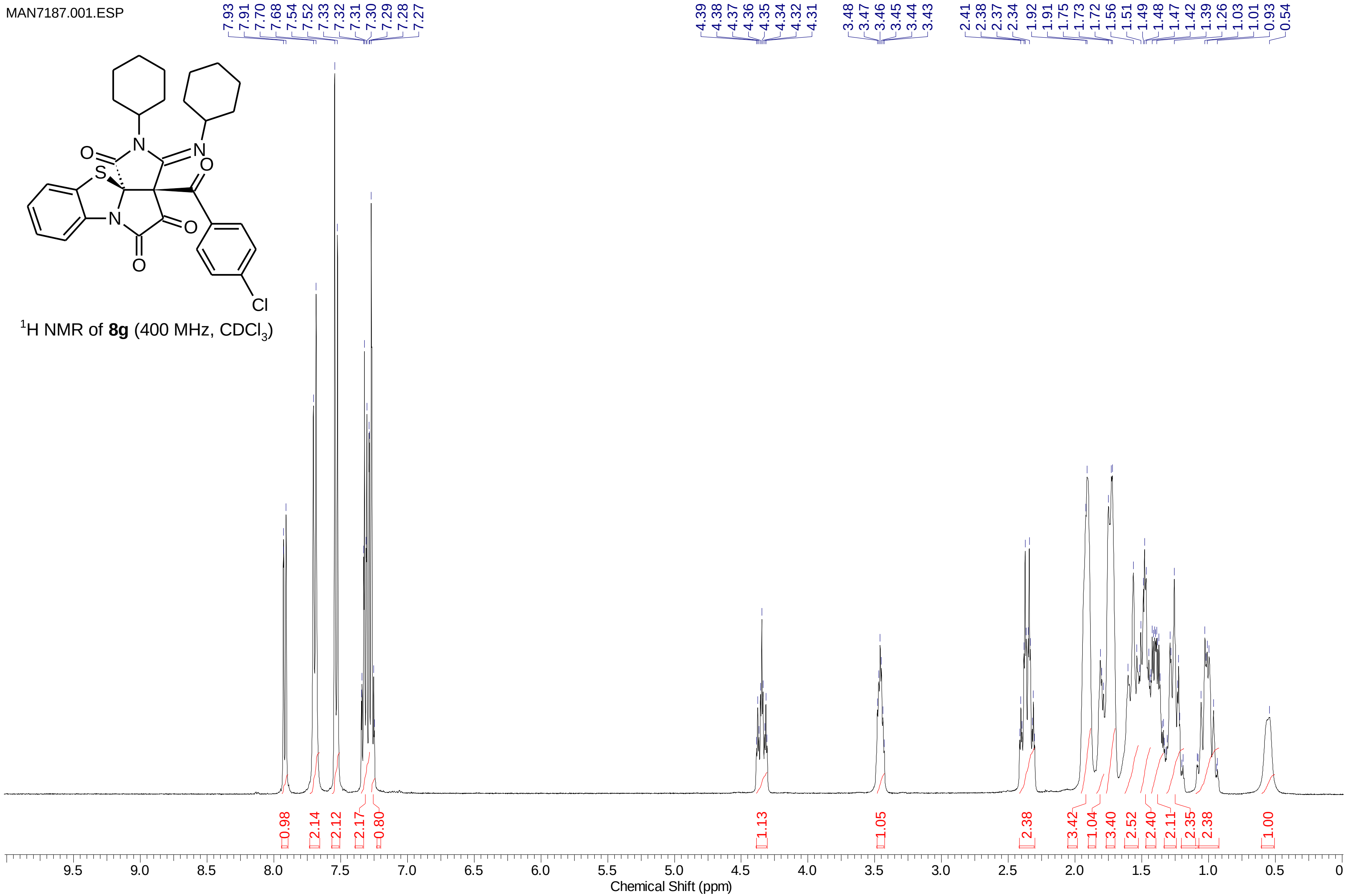


$^{1}$H NMR of **8g** (400 MHz, $CDCl_3$)

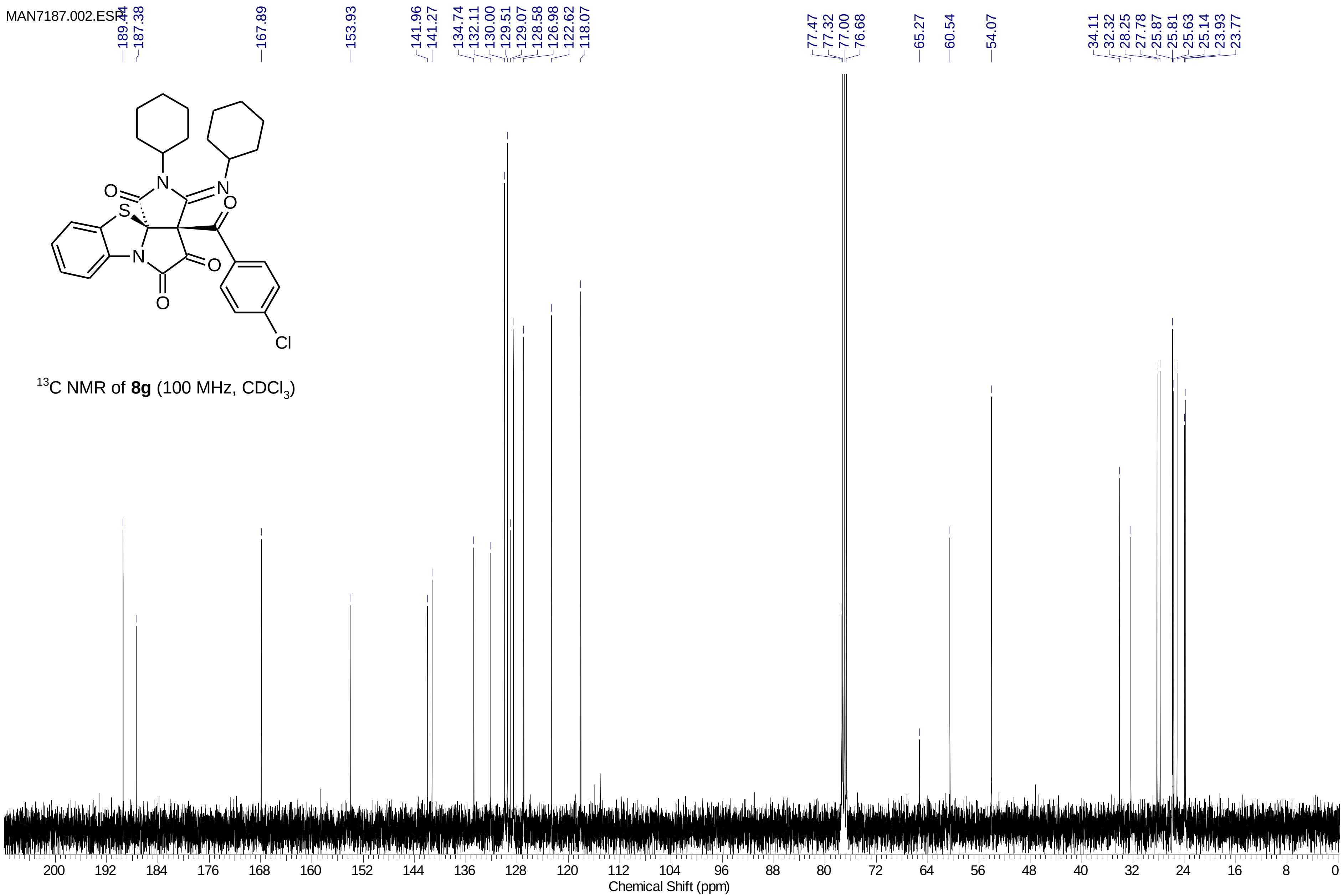


$^{13}$C NMR of **8g** (100 MHz, $CDCl_3$)

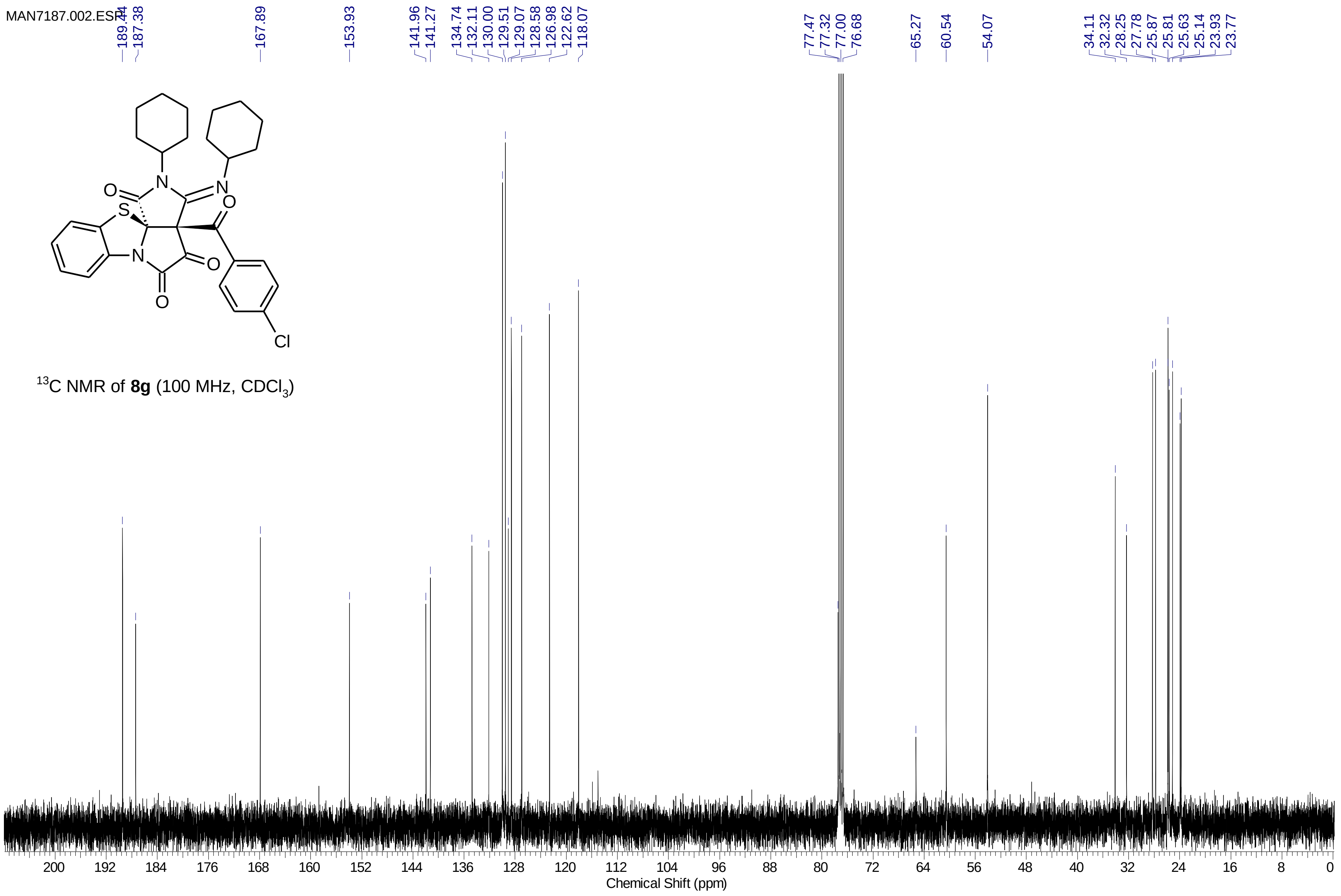


$^{13}$C NMR of **8g** (100 MHz, $CDCl_3$)

MAN7187.003.ESP

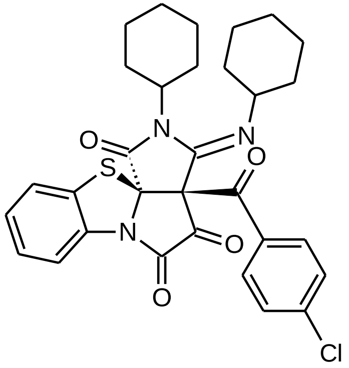

DEPT135 of **8g** (100 MHz, $CDCl_3$)

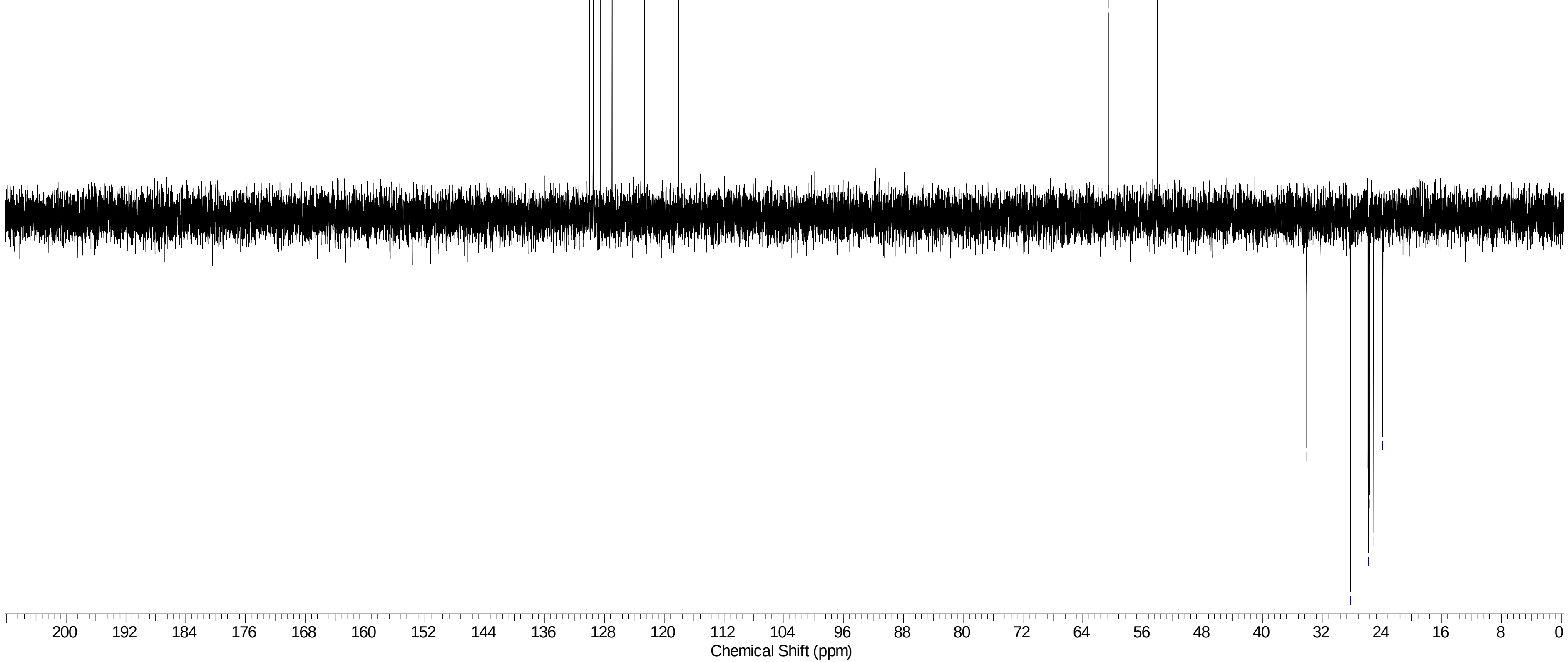

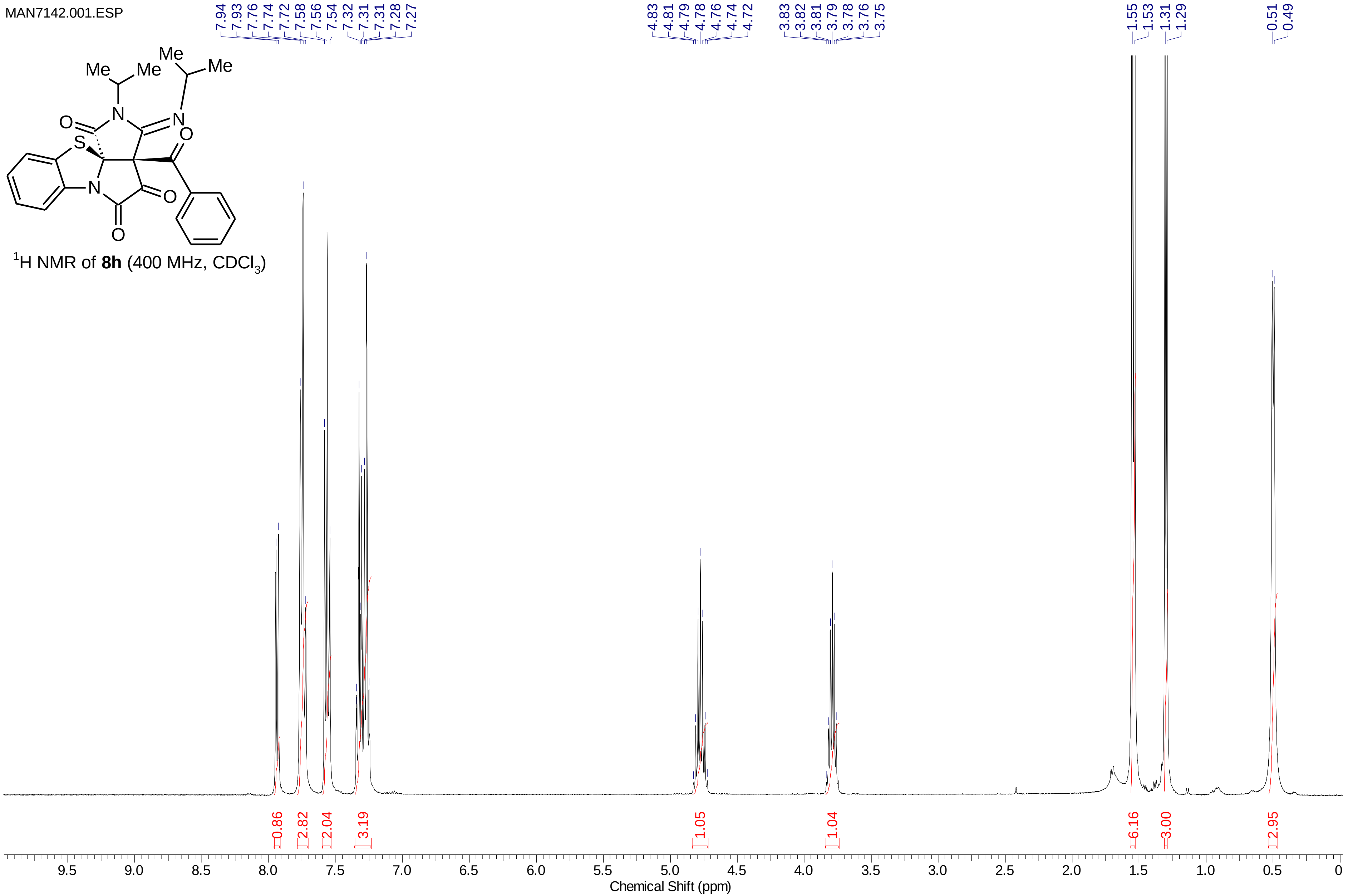


$^{1}$H NMR of **8h** (400 MHz, $CDCl_3$)

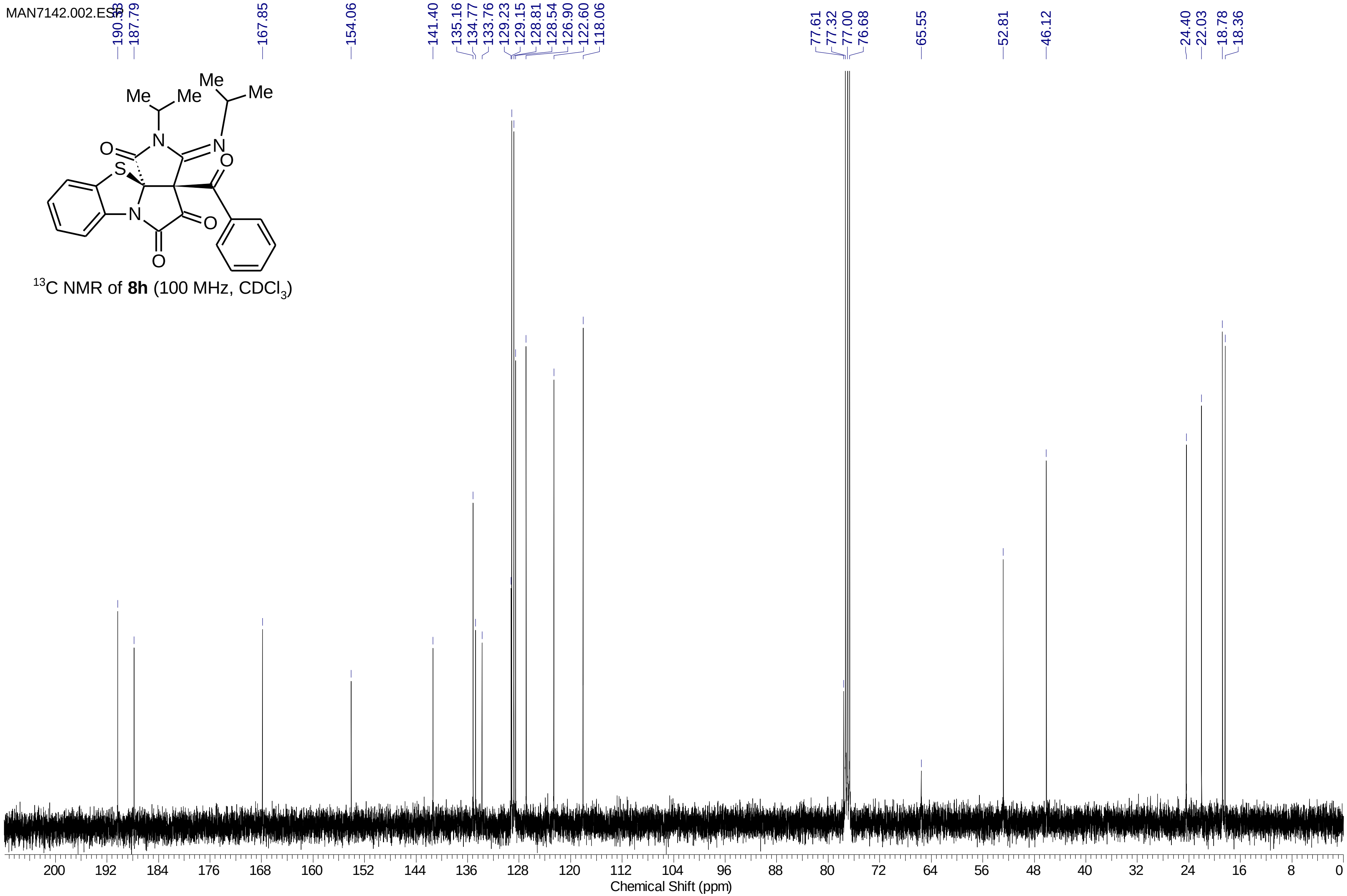


$^{13}$C NMR of **8h** (100 MHz, $CDCl_3$)

MAN7142.003.ESP

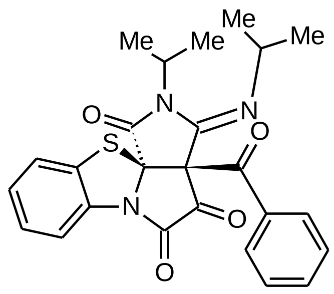


DEPT135 of **8h** (100 MHz, $CDCl_3$)

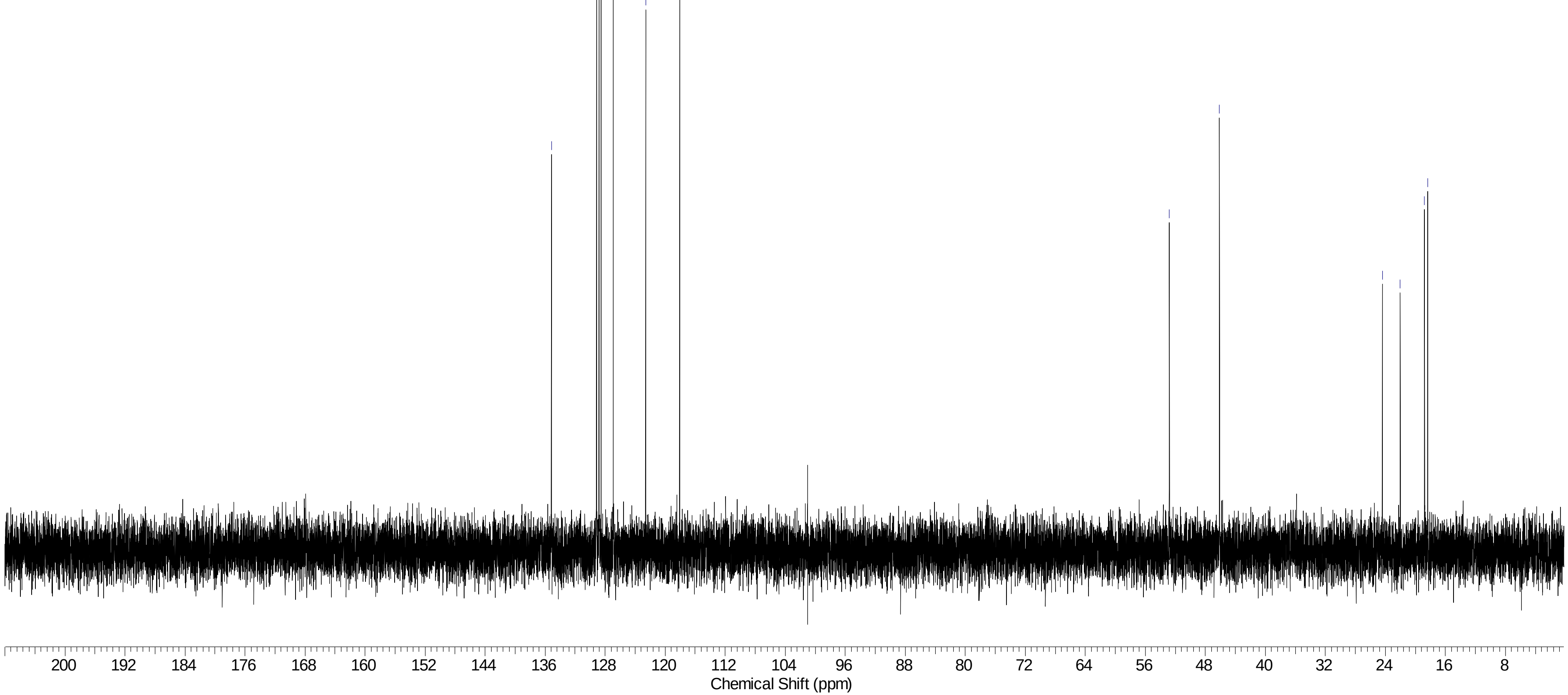

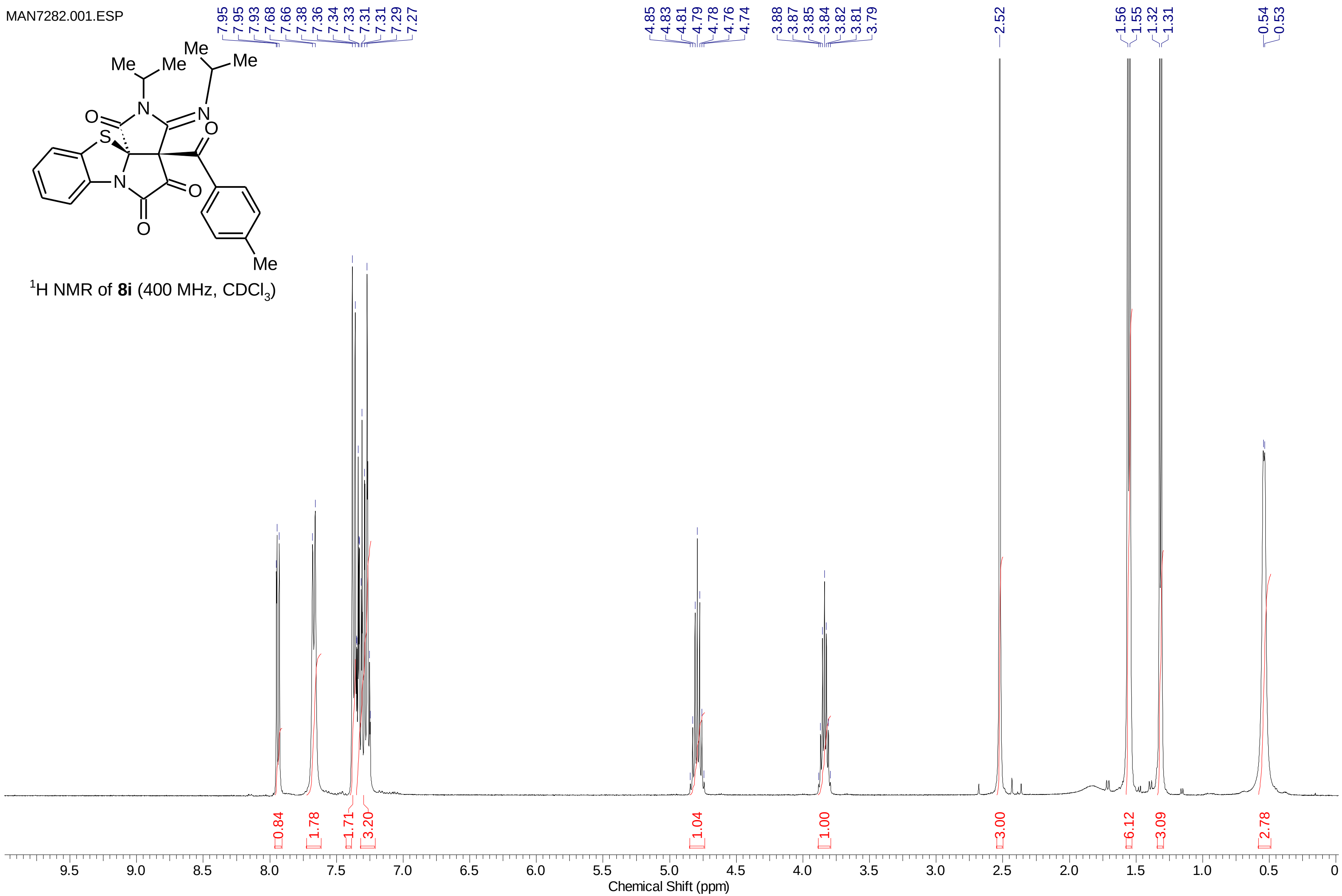


$^{1}$H NMR of **8i** (400 MHz, $CDCl_3$)

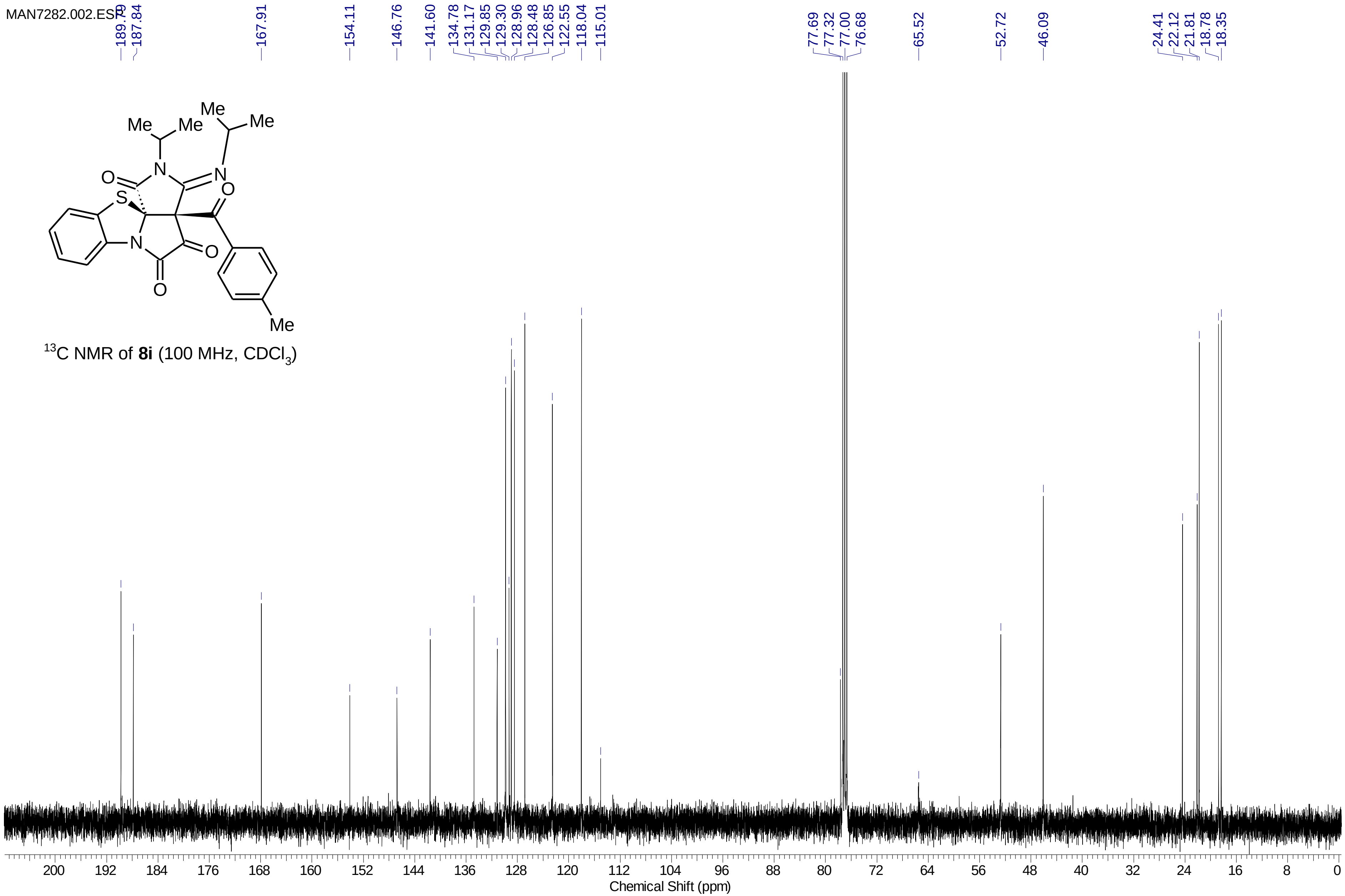


$^{13}C$ NMR of **8i** (100 MHz, $CDCl_3$)

MAN7282.003.ESP

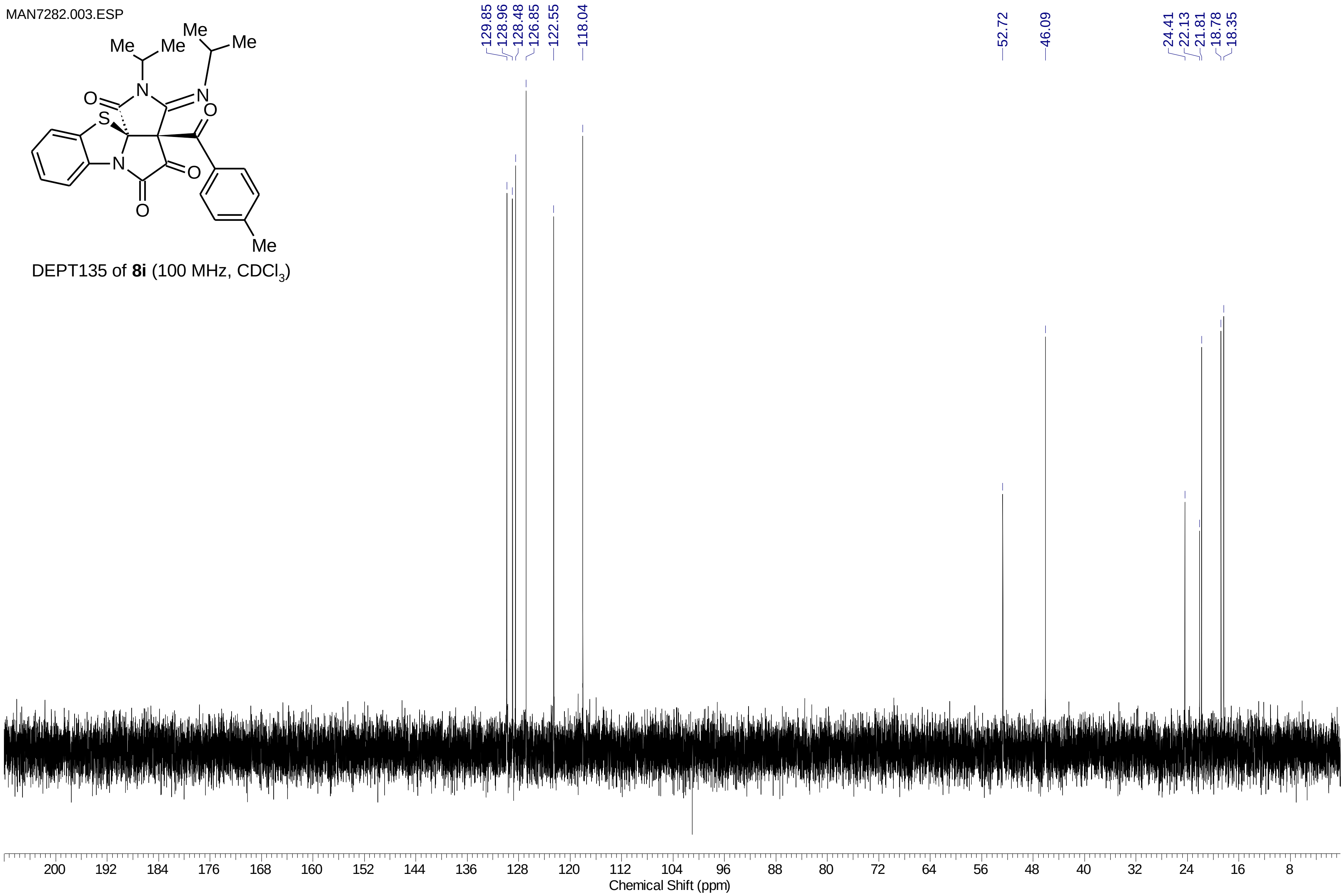


DEPT135 of **8i** (100 MHz, $CDCl_3$)

MAN7281.001.ESP

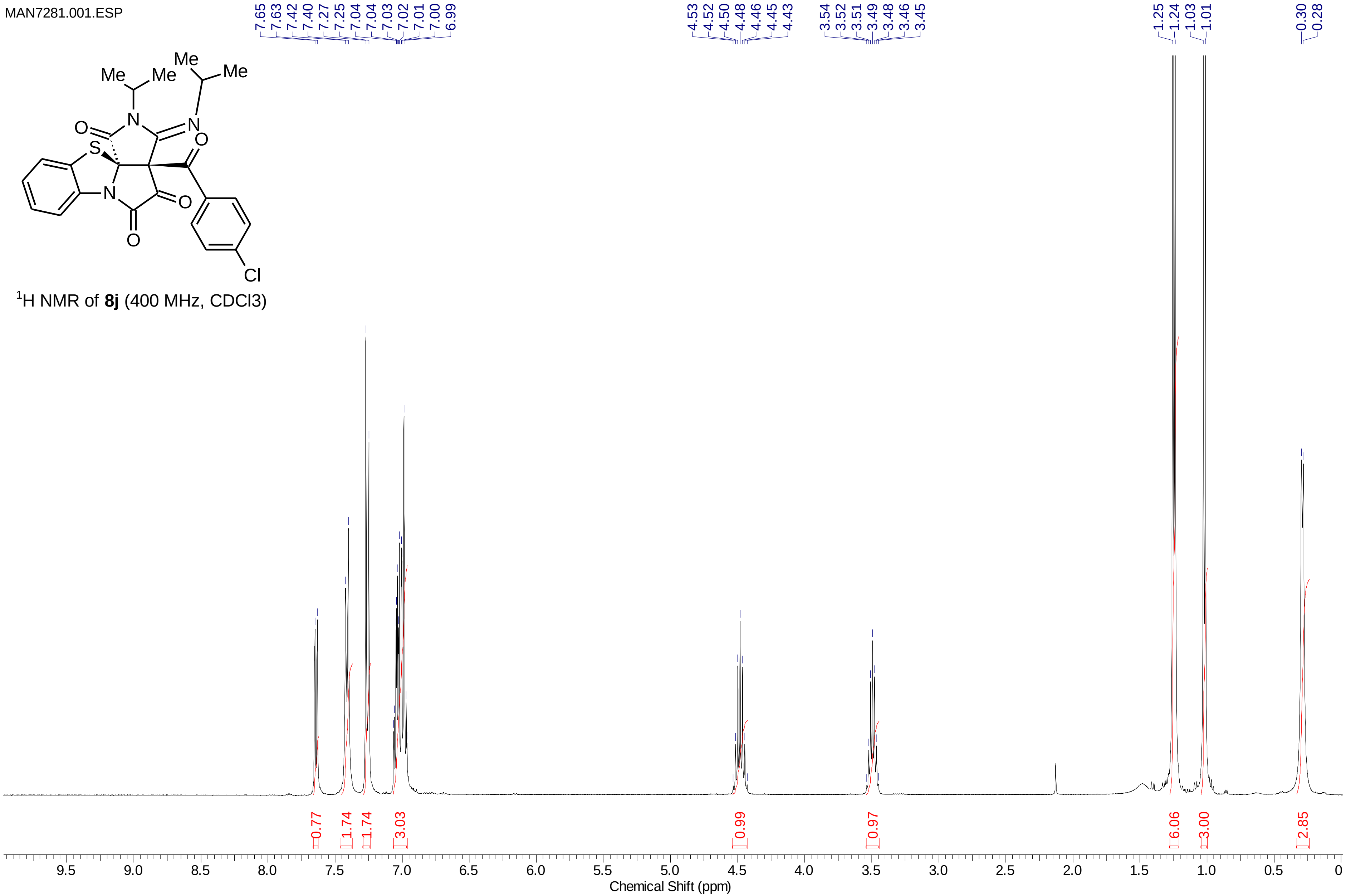


$^{1}$H NMR of **8j** (400 MHz, CDCl3)

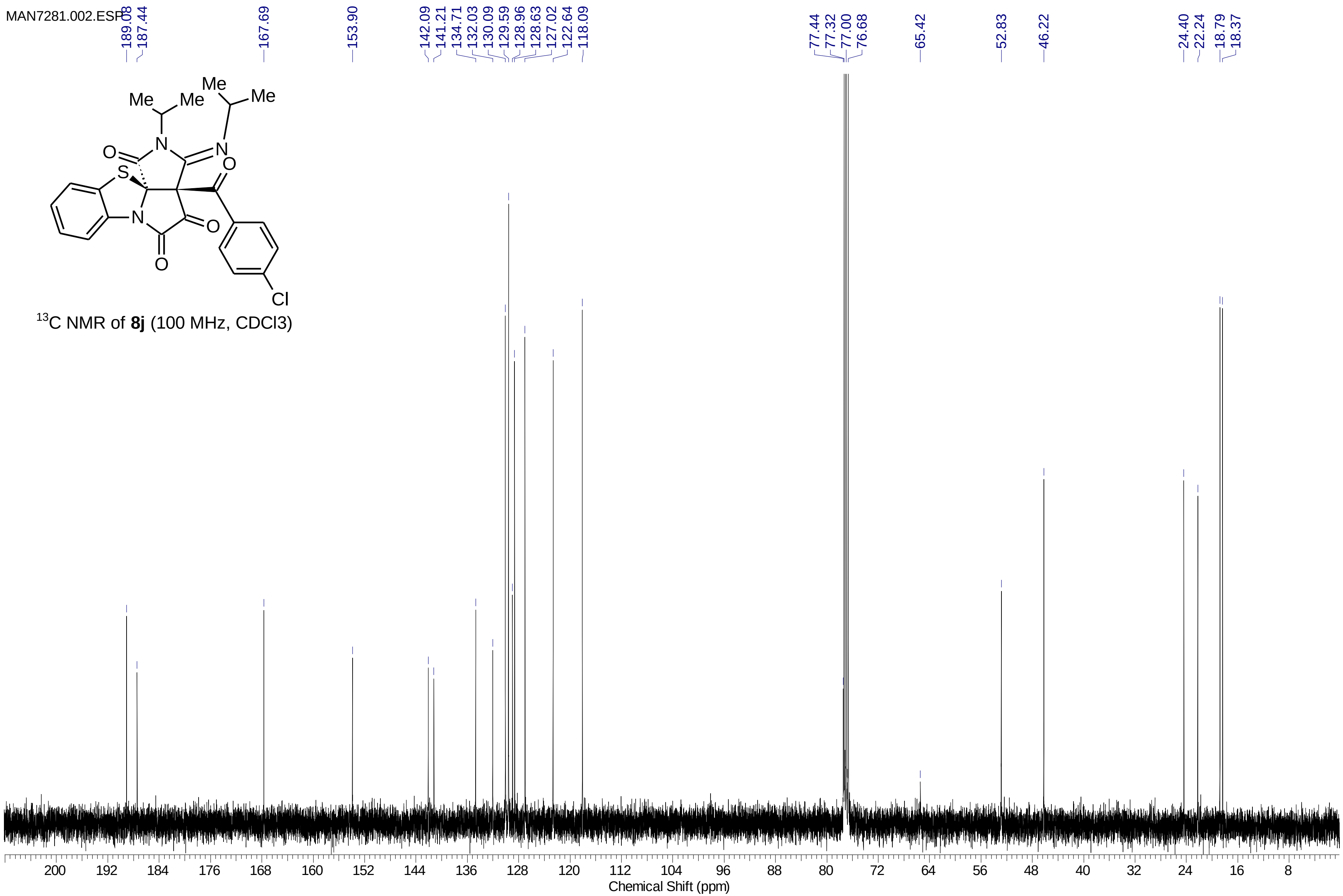


$^{13}$C NMR of **8j** (100 MHz, CDCl3)

MAN7281.003.ESP

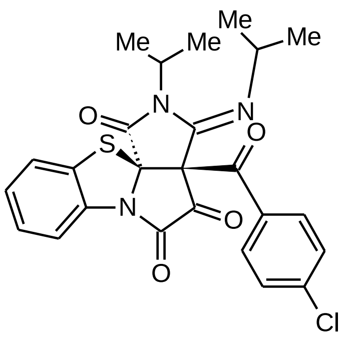

DEPT135 of **8j** (100 MHz, CDCl3)

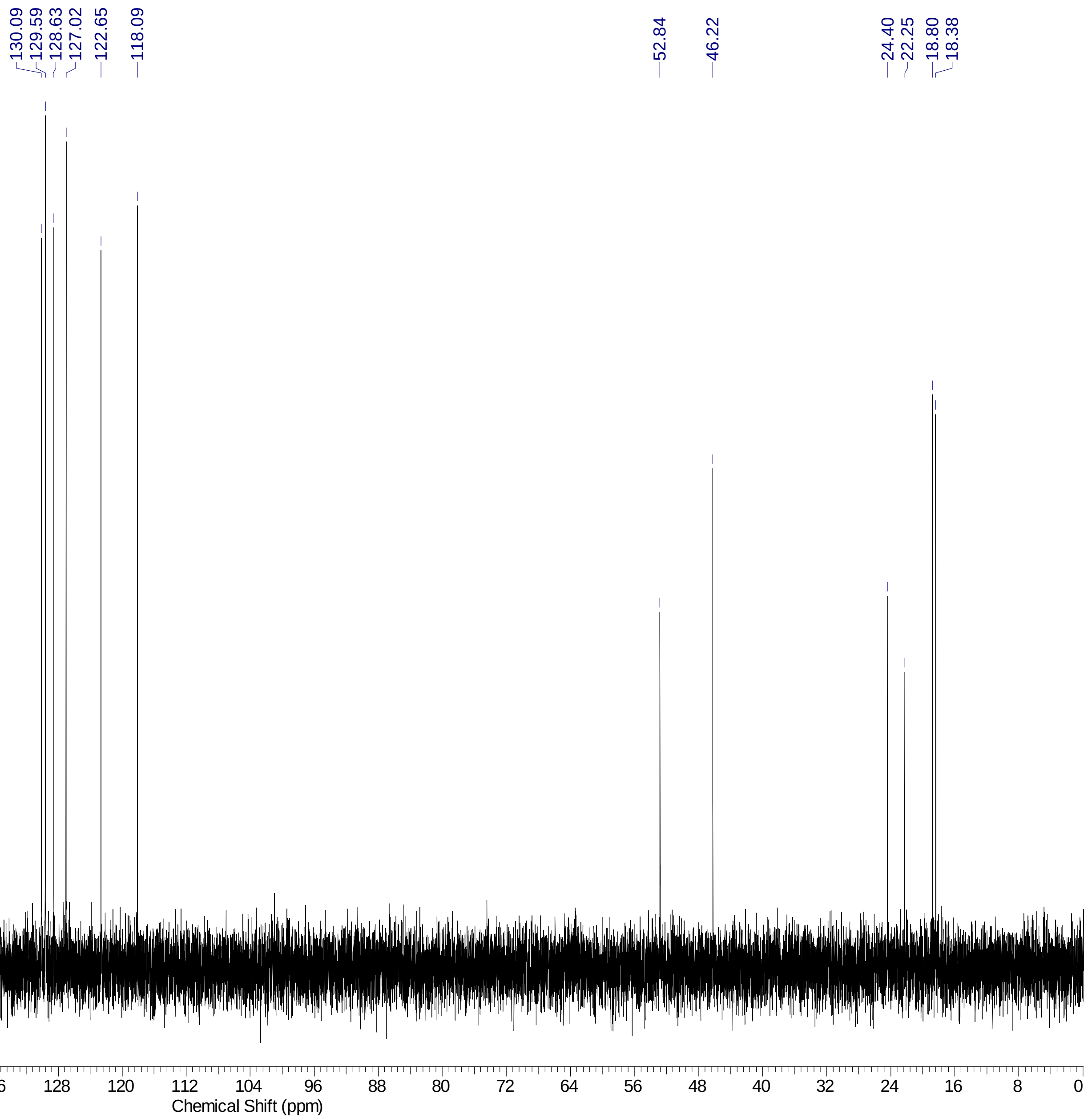

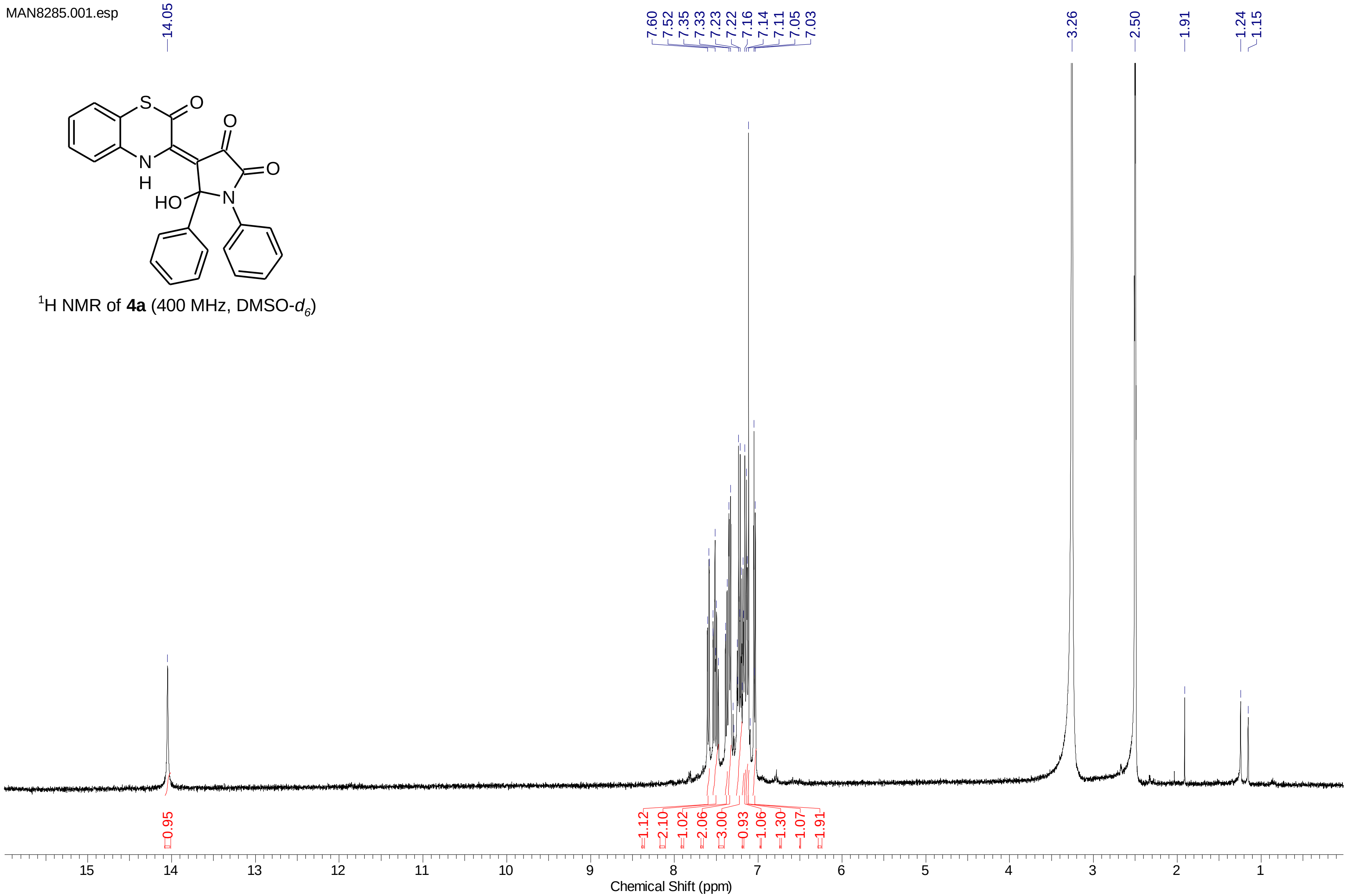
MAN8285.001.esp
14.05
7.60
7.52
7.35
7.33
7.23
7.22
7.16
7.14
7.11
7.05
7.03
3.26
2.50
1.91
1.24
1.15
S
O
O
O
N
H
HO
N
$^1$H NMR of **4a** (400 MHz, DMSO-$d_6$)
0.95
1.12
2.10
1.02
2.06
3.00
0.93
1.06
1.30
1.07
1.91
15
14
13
12
11
10
9
8
7
6
5
4
3
2
1
Chemical Shift (ppm)

MAN8465.001.esp

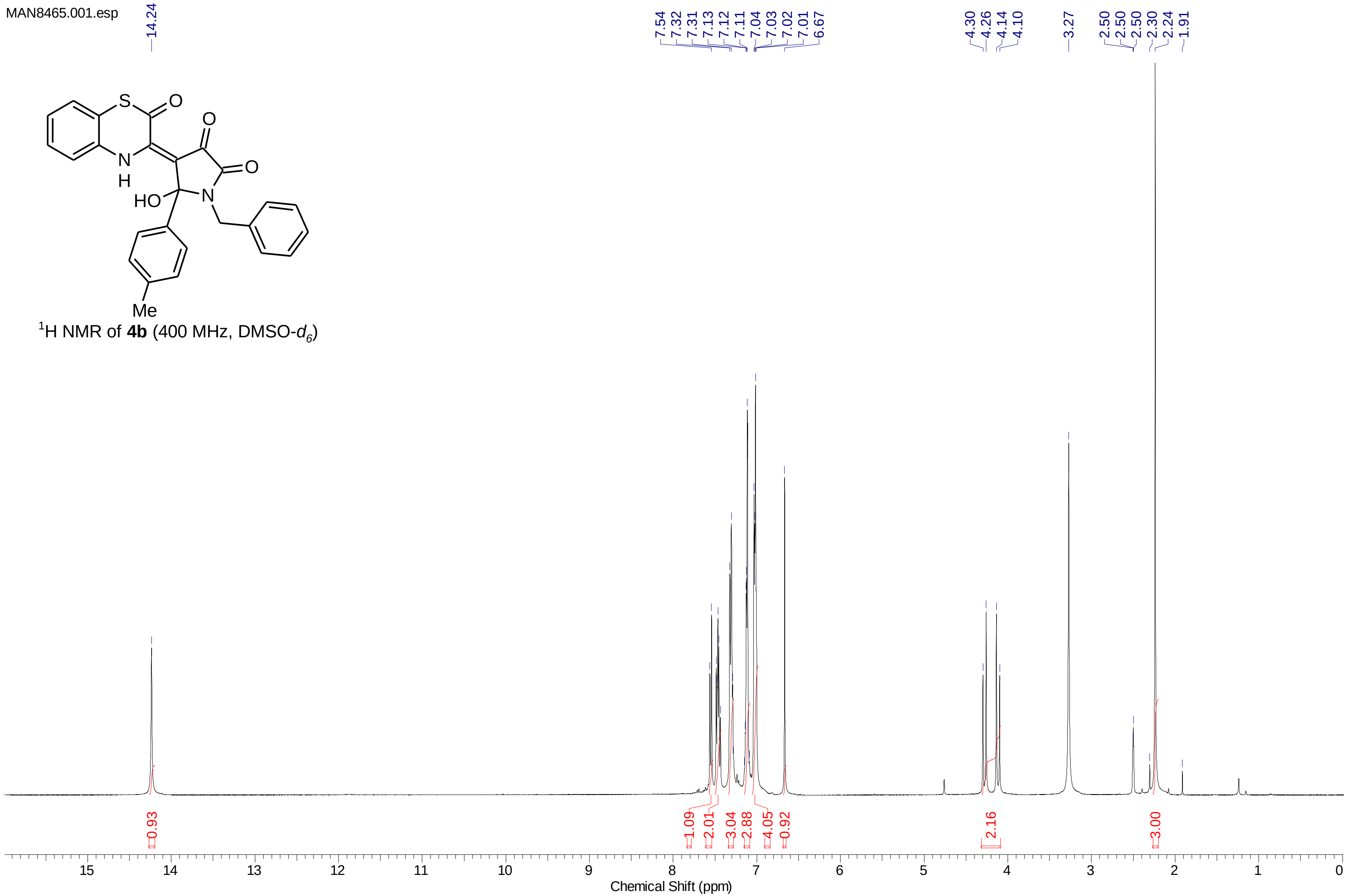


$^{1}$H NMR of **4b** (400 MHz, DMSO-$d_6$)

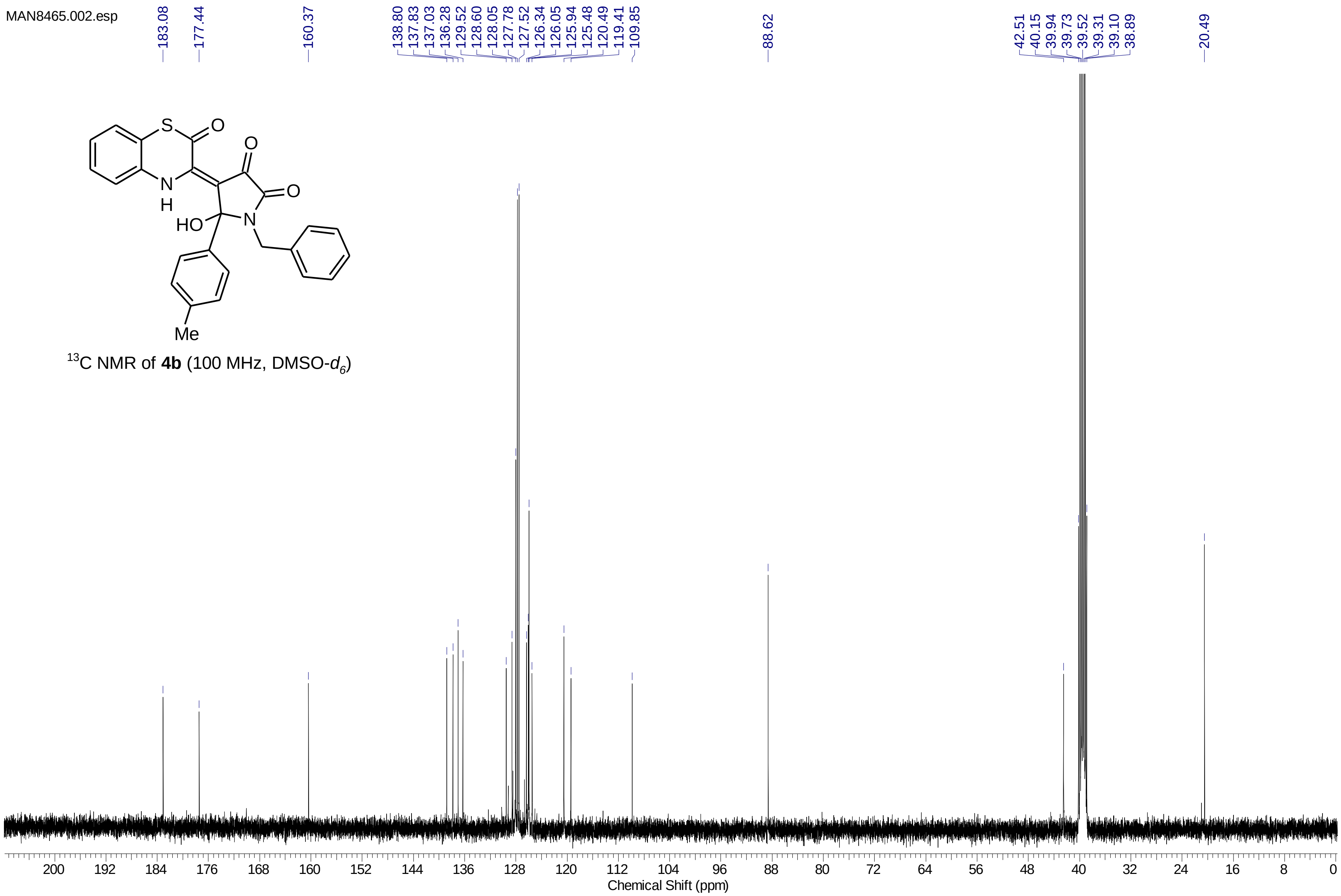


$^{13}$C NMR of **4b** (100 MHz, DMSO-$d_6$)

MAN8465.003.esp

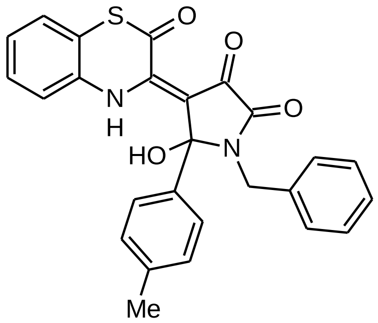


DEPT135 of **4b** (100 MHz, DMSO-$d_6$)

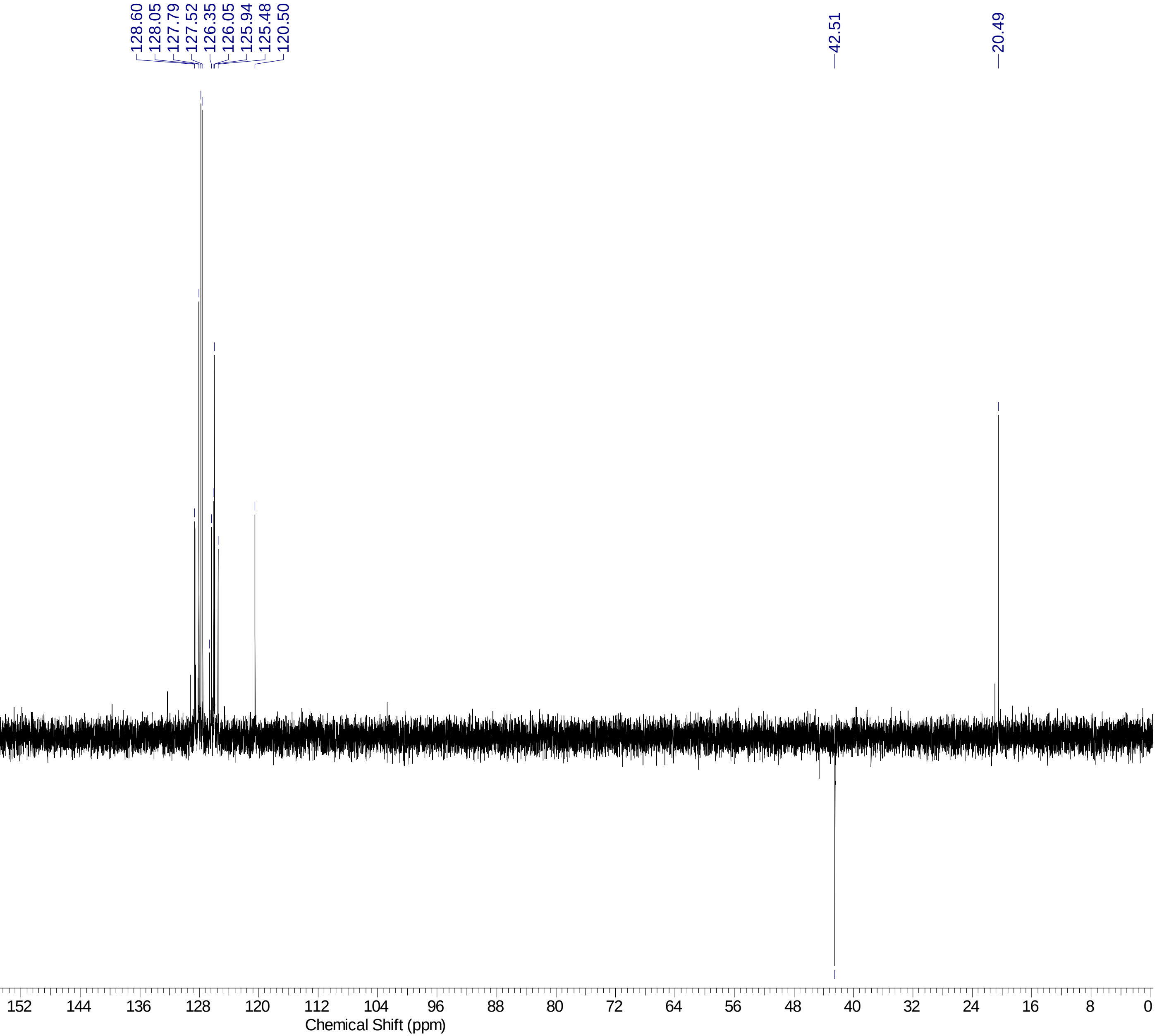

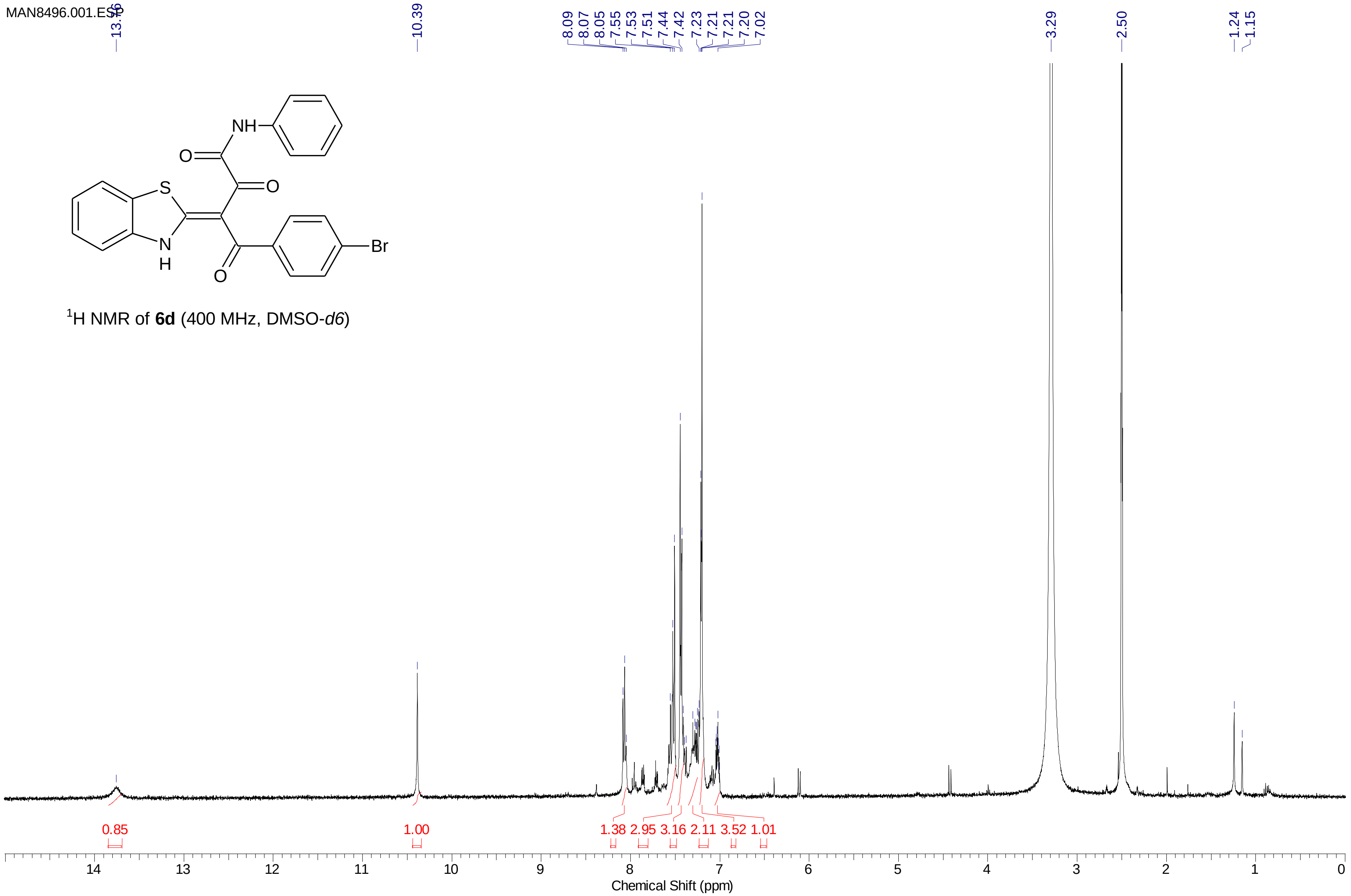


$^{1}$H NMR of **6d** (400 MHz, DMSO-*d6*)

**Calculated total electronic energies (E, in Hartree), enthalpies (H, in Hartree), Gibbs free energies (G, in Hartree), and entropies (S, cal/mol•K) for optimized equilibrium model structures.**

| Model structure | E | H | G | S |
|---|---|---|---|---|
| **2a** | -556.505478837 | -556.288000 | -556.337930 | 105.087 |
| **1a** | -1444.70735189 | -1444.455577 | -1444.525748 | 147.689 |
| **OC** | -2001.22427330 | -2000.752484 | -2000.853026 | 211.608 |
| **I2** | -2001.20499337 | -2000.732654 | -2000.826957 | 198.477 |
| **3a** | -2001.27537318 | -2000.801112 | -2000.892382 | 192.095 |
| **3'a** | -2001.28342773 | -2000.808838 | -2000.899955 | 191.773 |
| **TS** | -2001.21741473 | -2000.745789 | -2000.837371 | 192.749 |
| **TS’** | -2001.21575382 | -2000.744961 | -2000.834876 | 189.242 |

## ORTEP images of X-ray crystal structures of compounds 3a, 4b, 6d, 8a

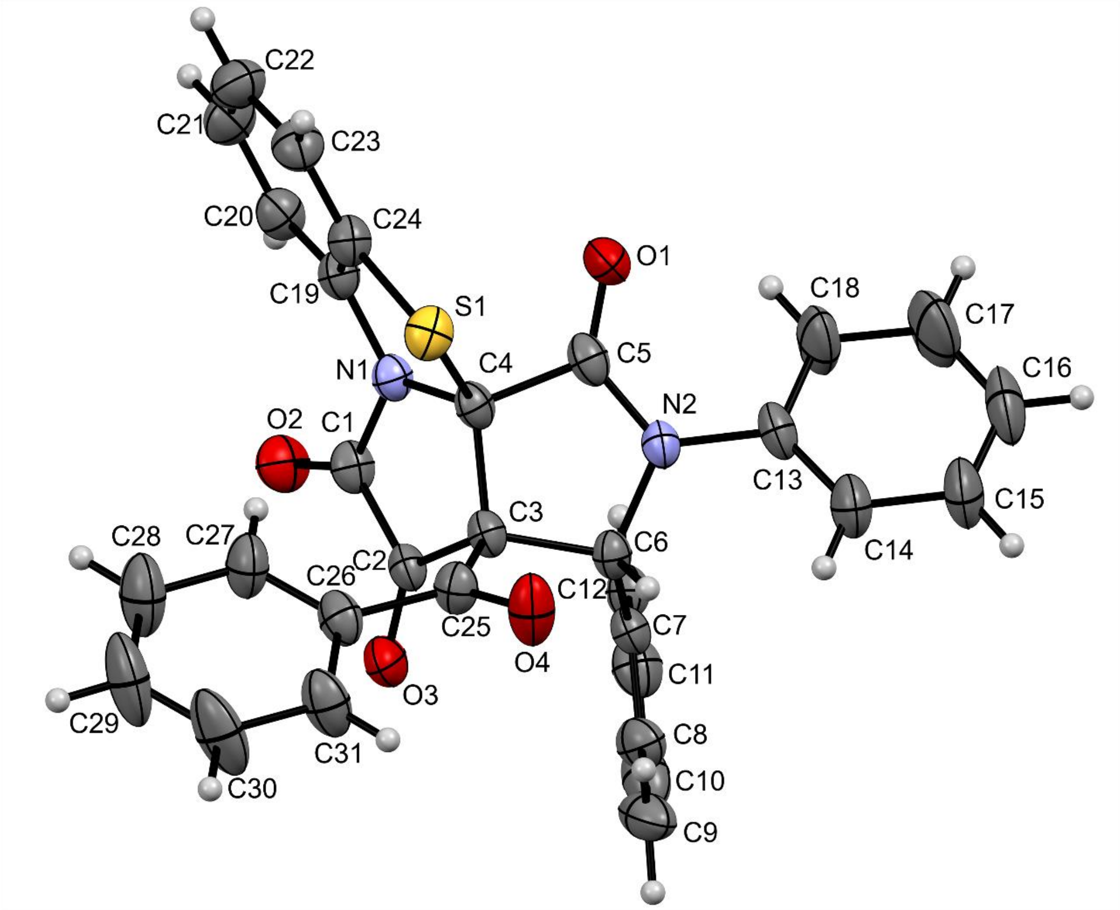


***Figure S1.*** *Molecular structure of compound* ***3a*** *showing 30% probability amplitude displacement ellipsoids (CCDC 2341688). Toluene solvate molecule is not shown.*

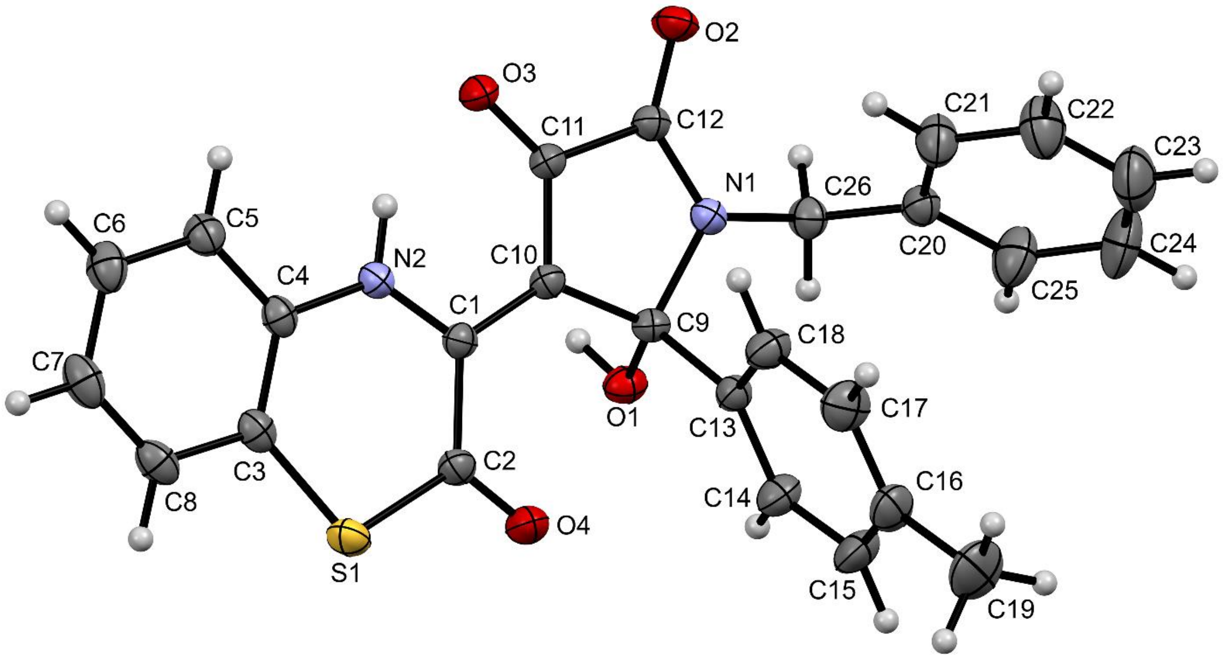


***Figure S2.*** *Molecular structure of compound* ***4b*** *showing 30% probability amplitude displacement ellipsoids (CCDC 2341690).*

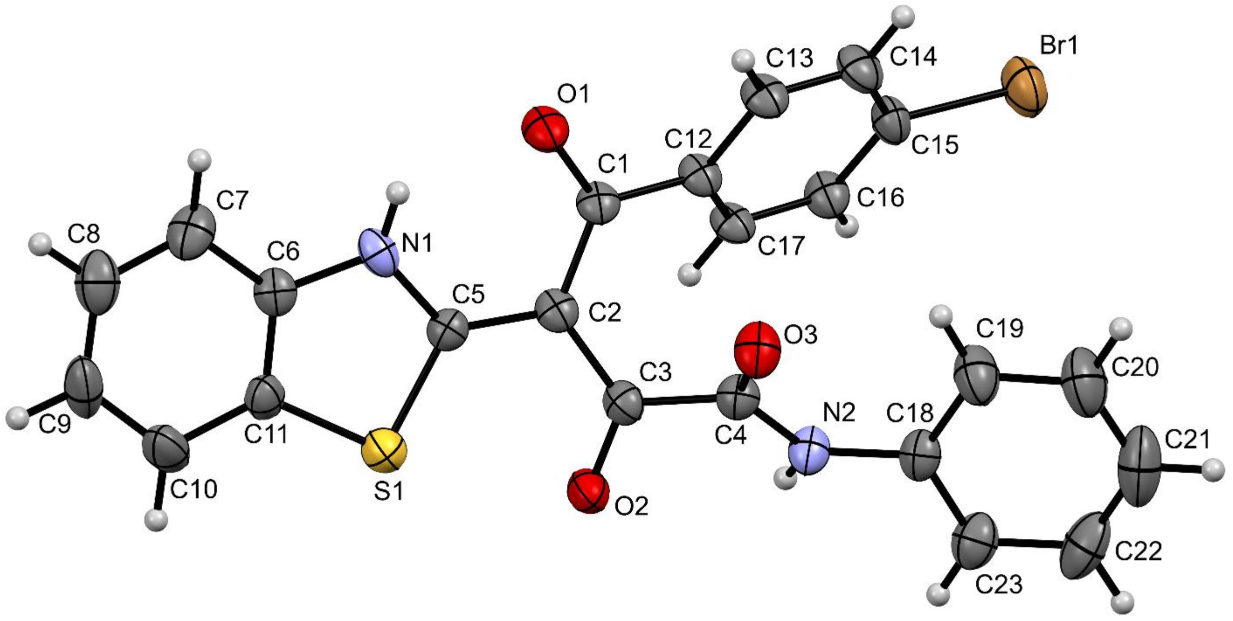


***Figure S3.*** *Molecular structure of compound* ***6d*** *showing 30% probability amplitude displacement ellipsoids (CCDC 2341691).*

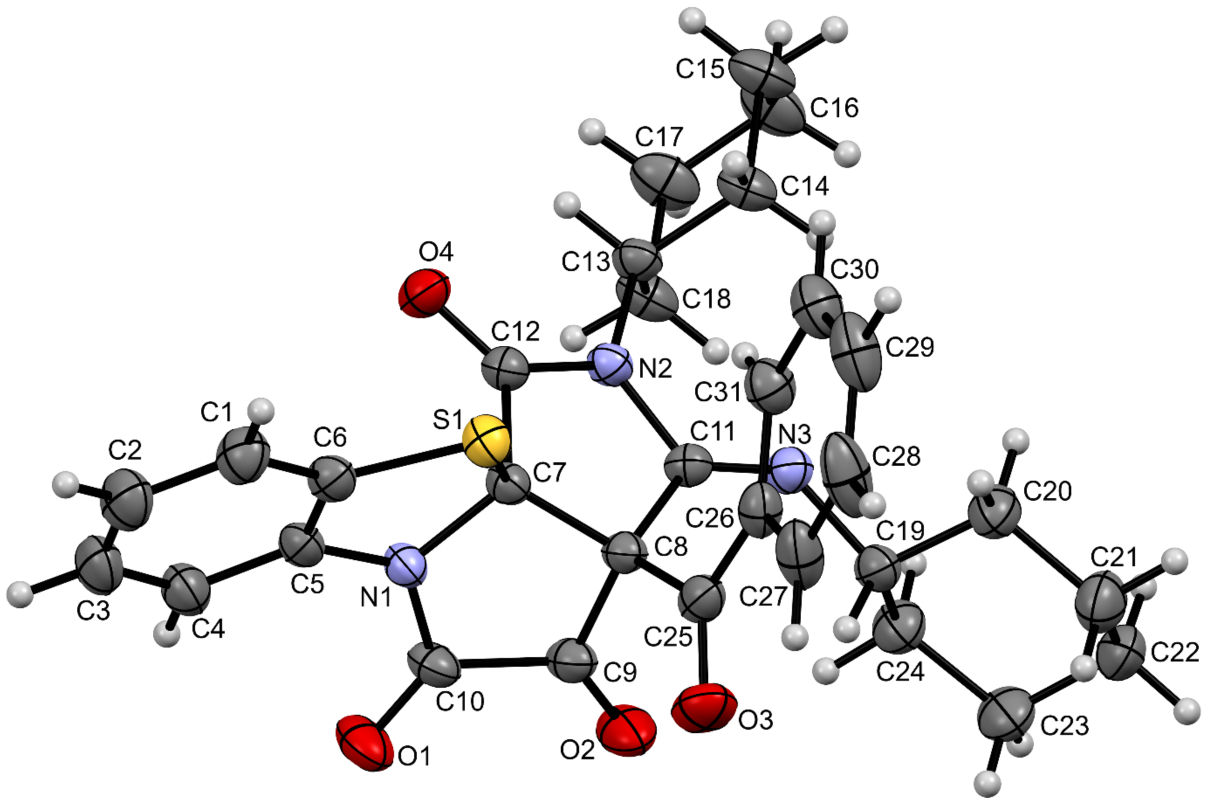


***Figure S4.*** *Molecular structure of compound **8a** showing 30% probability amplitude displacement ellipsoids (CCDC 2341689).*
