# Supplementary material for: Reaction of Pyrrolobenzothiazines with Schiff Bases and Carbodiimides: Approach to Angular 6/5/5/5-Tetracyclic Spiroheterocycles
Source: Molecules. 2024 May 1;29(9):2089. doi: 10.3390/molecules29092089 (PMC11085407; doi:10.3390/molecules29092089)

checkCIF/PLATON report

Structure factors have been supplied for datablock(s) 3a

THIS REPORT IS FOR GUIDANCE ONLY. IF USED AS PART OF A REVIEW PROCEDURE FOR PUBLICATION, IT SHOULD NOT REPLACE THE EXPERTISE OF AN EXPERIENCED CRYSTALLOGRAPHIC REFEREE.

No syntax errors found. CIF dictionary Interpreting this report

Datablock: 3a

```
Bond precision:   C-C = 0.0143 A          Wavelength=0.71073

Cell:          a=8.1892(18)       b=9.5061(16)      c=11.1371(19)
               alpha=86.021(14)   beta=71.607(18)   gamma=70.324(18)
Temperature:   295 K

                Calculated                   Reported
Volume          774.0(3)                     774.0(3)
Space group     P 1                          P 1
Hall group      P 1                          P 1
Moiety formula  C31 H20 N2 O4 S, C7 H8       C31 H20 N2 O4 S, C7 H8
Sum formula     C38 H28 N2 O4 S              C38 H28 N2 O4 S
Mr              608.68                       608.68
Dx,g cm-3       1.306                        1.306
Z               1                            1
Mu (mm-1)       0.149                        0.149
F000            318.0                        318.0
F000'           318.26
h,k,lmax        11,13,15                     11,13,15
Nref            8476[ 4238]                  5123
Tmin,Tmax       0.975,0.993                  0.795,1.000
Tmin'           0.968

Correction method= # Reported T Limits: Tmin=0.795 Tmax=1.000
AbsCorr = MULTI-SCAN

Data completeness= 1.21/0.60       Theta(max)= 29.328

                                                  wR2(reflections)=
R(reflections)= 0.0661( 3682)                     0.1916( 5123)

S = 1.084                  Npar= 408
```

The following ALERTS were generated. Each ALERT has the format
test-name_ALERT_alert-type_alert-level.
Click on the hyperlinks for more details of the test.

Alert level B

```
PLAT340_ALERT_3_B Low Bond Precision on  C-C Bonds ...............    0.01433 Ang.
PLAT915_ALERT_3_B No Flack x Check Done: Low Friedel Pair Coverage         35 %
```

Alert level C

```
PLAT230_ALERT_2_C Hirshfeld Test Diff for    N2       --C5       .       5.5 s.u.
PLAT234_ALERT_4_C Large Hirshfeld Difference C10      --C11      .      0.16 Ang.
PLAT234_ALERT_4_C Large Hirshfeld Difference C15      --C16      .      0.18 Ang.
PLAT234_ALERT_4_C Large Hirshfeld Difference C6S      --C7S      .      0.22 Ang.
PLAT241_ALERT_2_C High   'MainMol' Ueq as Compared to Neighbors of       C17 Check
PLAT241_ALERT_2_C High   'MainMol' Ueq as Compared to Neighbors of       C30 Check
PLAT242_ALERT_2_C Low    'MainMol' Ueq as Compared to Neighbors of       C13 Check
PLAT243_ALERT_4_C High   'Solvent' Ueq as Compared to Neighbors of       C3S Check
PLAT243_ALERT_4_C High   'Solvent' Ueq as Compared to Neighbors of       C6S Check
PLAT244_ALERT_4_C Low    'Solvent' Ueq as Compared to Neighbors of       C2S Check
PLAT244_ALERT_4_C Low    'Solvent' Ueq as Compared to Neighbors of       C7S Check
PLAT250_ALERT_2_C Large U3/U1 Ratio for <U(i,j)> Tensor(Resd    2)       2.9 Note
PLAT260_ALERT_2_C Large Average Ueq of Residue Including       C1S     0.196 Check
PLAT362_ALERT_2_C Short  C(sp3)-C(sp2) Bond  C1S      - C2S      .      1.39 Ang.
```

Alert level G

```
PLAT002_ALERT_2_G Number of Distance or Angle Restraints on AtSite         6 Note
PLAT003_ALERT_2_G Number of Uiso or Uij Restrained non-H Atoms ...         6 Report
PLAT172_ALERT_4_G The CIF-Embedded .res File Contains DFIX Records         1 Report
PLAT177_ALERT_4_G The CIF-Embedded .res File Contains DELU Records         1 Report
PLAT178_ALERT_4_G The CIF-Embedded .res File Contains SIMU Records         1 Report
PLAT192_ALERT_3_G A Non-default DELU Restraint Value for First Par    0.0050 Report
PLAT192_ALERT_3_G A Non-default DELU Restraint Value for SecondPar    0.0050 Report
PLAT231_ALERT_4_G Hirshfeld Test (Solvent)   C2S      --C7S      .       6.7 s.u.
PLAT720_ALERT_4_G Number of Unusual/Non-Standard Labels .........          3 Note
              H1SA    H1SB    H1SC
PLAT860_ALERT_3_G Number of Least-Squares Restraints ............         47 Note
PLAT910_ALERT_3_G Missing # of FCF Reflection(s) Below Theta(Min).         1 Note
               0  0  1,
PLAT912_ALERT_4_G Missing # of FCF Reflections Above STh/L=  0.600       611 Note
PLAT933_ALERT_2_G Number of HKL-OMIT Records in Embedded .res File         4 Note
               -2 -2  8,  -1  1  9,  -4 -8 -5,  -4 -4  6,
PLAT941_ALERT_3_G Average HKL Measurement Multiplicity ..........        1.9 Low
PLAT969_ALERT_5_G The 'Henn et al.' R-Factor-gap value ..........       1.98 Note
              Predicted wR2: Based on SigI**2  9.66 or SHELX Weight 18.43
PLAT978_ALERT_2_G Number C-C Bonds with Positive Residual Density.         1 Info
PLAT992_ALERT_5_G Repd & Actual _reflns_number_gt Values Differ by         7 Check
```

0 **ALERT level A** = Most likely a serious problem - resolve or explain
2 **ALERT level B** = A potentially serious problem, consider carefully
14 **ALERT level C** = Check. Ensure it is not caused by an omission or oversight
17 **ALERT level G** = General information/check it is not something unexpected

```
 0 ALERT type 1 CIF construction/syntax error, inconsistent or missing data
11 ALERT type 2 Indicator that the structure model may be wrong or deficient
 7 ALERT type 3 Indicator that the structure quality may be low
13 ALERT type 4 Improvement, methodology, query or suggestion
 2 ALERT type 5 Informative message, check
```

It is advisable to attempt to resolve as many as possible of the alerts in all categories. Often the minor alerts point to easily fixed oversights, errors and omissions in your CIF or refinement strategy, so attention to these fine details can be worthwhile. In order to resolve some of the more serious problems it may be necessary to carry out additional measurements or structure refinements. However, the purpose of your study may justify the reported deviations and the more serious of these should normally be commented upon in the discussion or experimental section of a paper or in the "special_details" fields of the CIF. checkCIF was carefully designed to identify outliers and unusual parameters, but every test has its limitations and alerts that are not important in a particular case may appear. Conversely, the absence of alerts does not guarantee there are no aspects of the results needing attention. It is up to the individual to critically assess their own results and, if necessary, seek expert advice.

Publication of your CIF in IUCr journals

A basic structural check has been run on your CIF. These basic checks will be run on all CIFs submitted for publication in IUCr journals (*Acta Crystallographica*, *Journal of Applied Crystallography*, *Journal of Synchrotron Radiation*); however, if you intend to submit to *Acta Crystallographica Section C* or *E* or *IUCrData*, you should make sure that full publication checks are run on the final version of your CIF prior to submission.

Publication of your CIF in other journals

Please refer to the *Notes for Authors* of the relevant journal for any special instructions relating to CIF submission.

PLATON version of 06/01/2024; check.def file version of 05/01/2024

Datablock 3a - ellipsoid plot

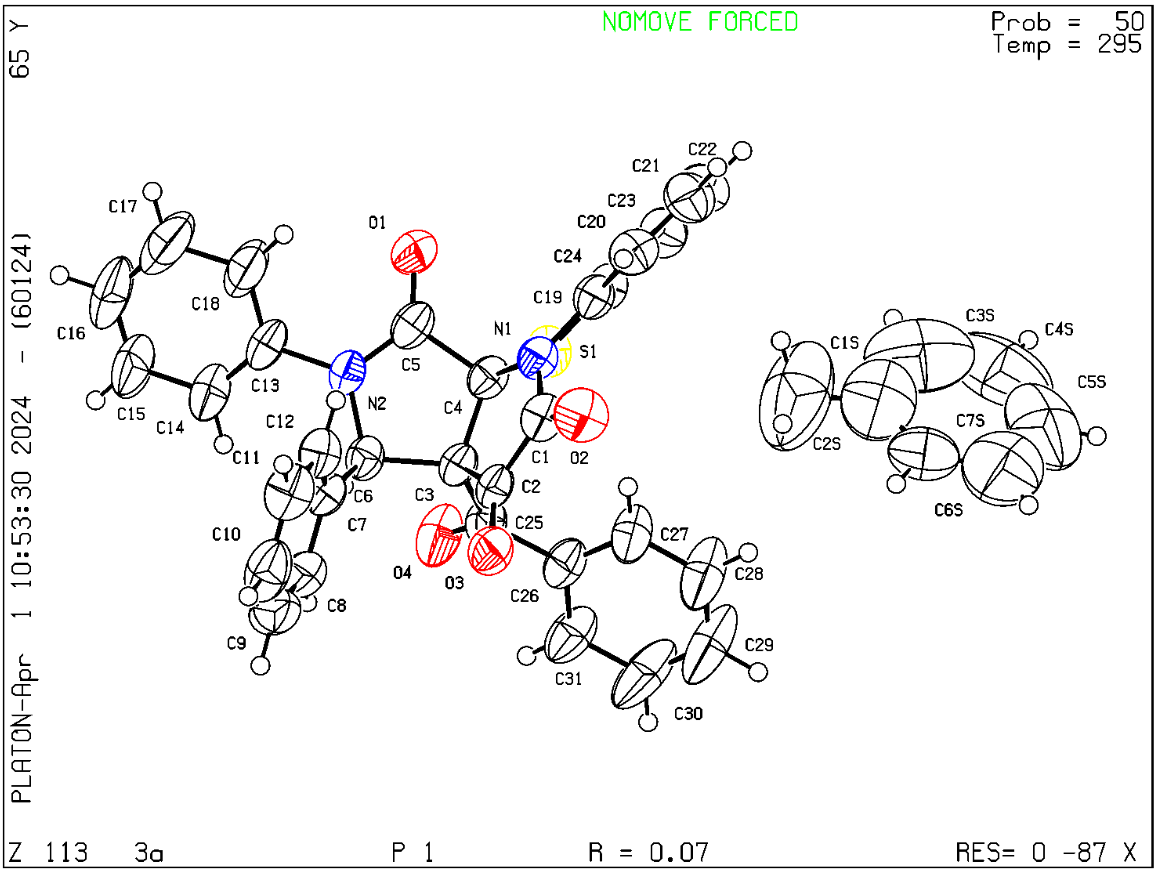

checkCIF/PLATON report

Structure factors have been supplied for datablock(s) compound_4b

THIS REPORT IS FOR GUIDANCE ONLY. IF USED AS PART OF A REVIEW PROCEDURE FOR PUBLICATION, IT SHOULD NOT REPLACE THE EXPERTISE OF AN EXPERIENCED CRYSTALLOGRAPHIC REFEREE.

No syntax errors found. CIF dictionary Interpreting this report

Datablock: compound_4b

Bond precision: C-C = 0.0030 A Wavelength=0.71073

Cell:	a=13.548(2)	b=7.2804(11)	c=23.188(4)
	alpha=90	beta=103.495(15)	gamma=90
Temperature:	295 K		

	Calculated	Reported
Volume	2224.0(6)	2224.0(6)
Space group	P 21/n	P 1 21/n 1
Hall group	-P 2yn	-P 2yn
Moiety formula	C26 H20 N2 O4 S	C26 H20 N2 O4 S
Sum formula	C26 H20 N2 O4 S	C26 H20 N2 O4 S
Mr	456.50	456.50
Dx,g cm-3	1.363	1.363
Z	4	4
Mu (mm-1)	0.182	0.182
F000	952.0	952.0
F000'	952.92	
h,k,lmax	18,10,32	18,10,30
Nref	6170	5254
Tmin,Tmax	0.937,0.964	0.749,1.000
Tmin'	0.897	

Correction method= # Reported T Limits: Tmin=0.749 Tmax=1.000
AbsCorr = MULTI-SCAN

Data completeness= 0.852 Theta(max)= 29.456

R(reflections)= 0.0469(3911) wR2(reflections)= 0.1268(5254)

S = 1.031 Npar= 307

The following ALERTS were generated. Each ALERT has the format
test-name_ALERT_alert-type_alert-level.
Click on the hyperlinks for more details of the test.

Alert level C

```
PLAT242_ALERT_2_C Low    'MainMol' Ueq as Compared to Neighbors of        C20 Check
PLAT906_ALERT_3_C Large K Value in the Analysis of Variance ......      2.752 Check
```

Alert level G

```
PLAT910_ALERT_3_G Missing # of FCF Reflection(s) Below Theta(Min).          2 Note
               -1  0  1,   0  0  2,
PLAT912_ALERT_4_G Missing # of FCF Reflections Above STh/L=  0.600        894 Note
PLAT941_ALERT_3_G Average HKL Measurement Multiplicity ..........         2.3 Low
PLAT952_ALERT_5_G Calculated (ThMax) and CIF-Reported Lmax Differ.          2 Units
PLAT958_ALERT_1_G Calculated (ThMax) and Actual (FCF) Lmax Differ.          2 Units
PLAT969_ALERT_5_G The 'Henn et al.' R-Factor-gap value ..........        2.46 Note
              Predicted wR2: Based on SigI**2  5.14 or SHELX Weight 12.68
PLAT978_ALERT_2_G Number C-C Bonds with Positive Residual Density.          6 Info
```

0 **ALERT level A** = Most likely a serious problem - resolve or explain
0 **ALERT level B** = A potentially serious problem, consider carefully
2 **ALERT level C** = Check. Ensure it is not caused by an omission or oversight
7 **ALERT level G** = General information/check it is not something unexpected

1 ALERT type 1 CIF construction/syntax error, inconsistent or missing data
2 ALERT type 2 Indicator that the structure model may be wrong or deficient
3 ALERT type 3 Indicator that the structure quality may be low
1 ALERT type 4 Improvement, methodology, query or suggestion
2 ALERT type 5 Informative message, check

It is advisable to attempt to resolve as many as possible of the alerts in all categories. Often the minor alerts point to easily fixed oversights, errors and omissions in your CIF or refinement strategy, so attention to these fine details can be worthwhile. In order to resolve some of the more serious problems it may be necessary to carry out additional measurements or structure refinements. However, the purpose of your study may justify the reported deviations and the more serious of these should normally be commented upon in the discussion or experimental section of a paper or in the "special_details" fields of the CIF. checkCIF was carefully designed to identify outliers and unusual parameters, but every test has its limitations and alerts that are not important in a particular case may appear. Conversely, the absence of alerts does not guarantee there are no aspects of the results needing attention. It is up to the individual to critically assess their own results and, if necessary, seek expert advice.

Publication of your CIF in IUCr journals

A basic structural check has been run on your CIF. These basic checks will be run on all CIFs submitted for publication in IUCr journals (*Acta Crystallographica*, *Journal of Applied Crystallography*, *Journal of Synchrotron Radiation*); however, if you intend to submit to *Acta Crystallographica Section C* or *E* or *IUCrData*, you should make sure that full publication checks are run on the final version of your CIF prior to submission.

Publication of your CIF in other journals

Please refer to the *Notes for Authors* of the relevant journal for any special instructions relating to CIF submission.

PLATON version of 06/01/2024; check.def file version of 05/01/2024

Datablock compound_4b - ellipsoid plot

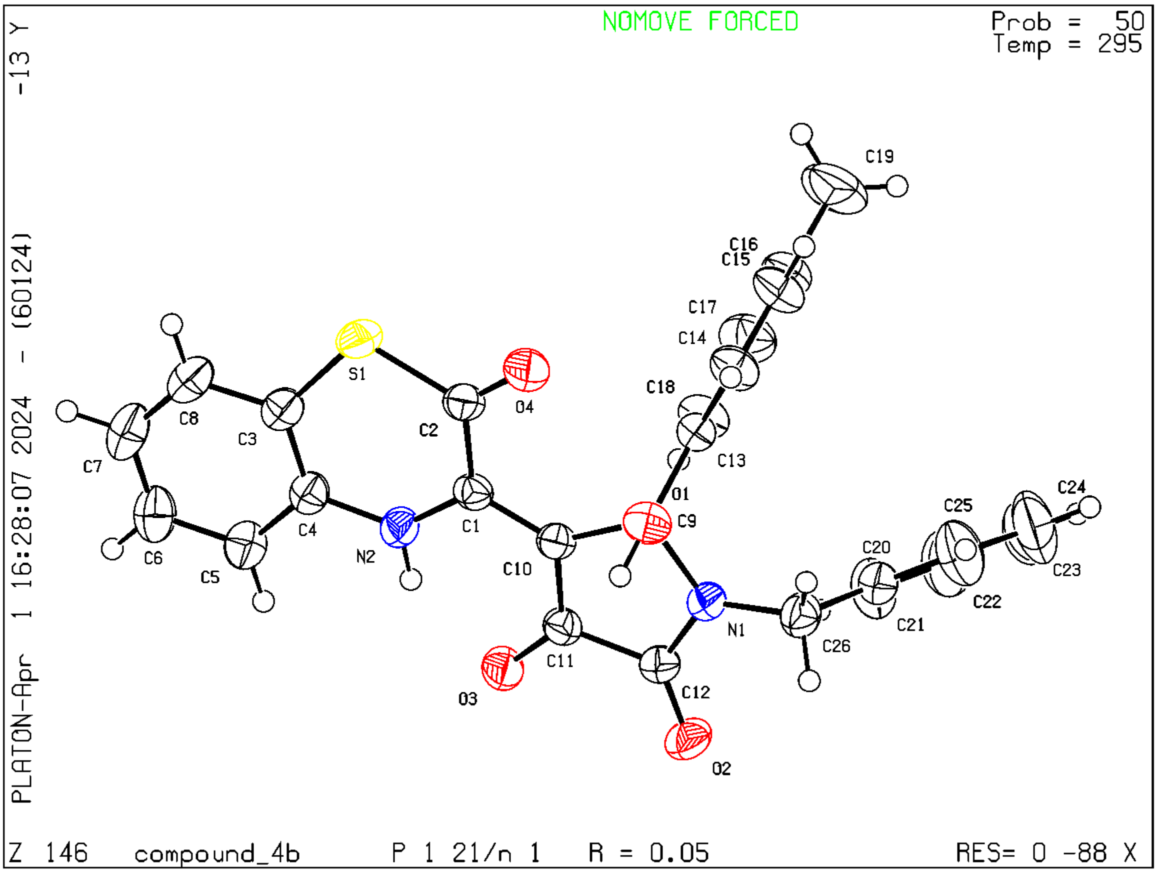

checkCIF/PLATON report

Structure factors have been supplied for datablock(s) compound_6d

THIS REPORT IS FOR GUIDANCE ONLY. IF USED AS PART OF A REVIEW PROCEDURE FOR PUBLICATION, IT SHOULD NOT REPLACE THE EXPERTISE OF AN EXPERIENCED CRYSTALLOGRAPHIC REFEREE.

No syntax errors found. CIF dictionary Interpreting this report

Datablock: compound_6d

```
Bond precision:   C-C = 0.0072 A          Wavelength=0.71073

Cell:             a=20.406(4)     b=9.309(3)           c=11.069(2)
                  alpha=90        beta=93.739(19)      gamma=90
Temperature:      295 K

                Calculated                  Reported
Volume          2098.2(9)                   2098.2(9)
Space group     P 21/c                      P 1 21/c 1
Hall group      -P 2ybc                     -P 2ybc
Moiety formula  C23 H15 Br N2 O3 S          C23 H15 Br N2 O3 S
Sum formula     C23 H15 Br N2 O3 S          C23 H15 Br N2 O3 S
Mr              479.33                      479.34
Dx,g cm-3       1.517                       1.517
Z               4                           4
Mu (mm-1)       2.086                       2.086
F000            968.0                       968.0
F000'           967.72
h,k,lmax        28,12,15                    27,12,15
Nref            5758                        4934
Tmin,Tmax       0.644,0.864                 0.361,1.000
Tmin'           0.349

Correction method= # Reported T Limits: Tmin=0.361 Tmax=1.000
AbsCorr = MULTI-SCAN

Data completeness= 0.857          Theta(max)= 29.353

                                                    wR2(reflections)=
R(reflections)= 0.0670( 2424)                       0.2110( 4934)

S = 1.009                  Npar= 278
```

The following ALERTS were generated. Each ALERT has the format
test-name_ALERT_alert-type_alert-level.
Click on the hyperlinks for more details of the test.

Alert level B

```
PLAT230_ALERT_2_B Hirshfeld Test Diff for    N1       --C5       .        9.0 s.u.
```

Alert level C

```
PLAT026_ALERT_3_C Ratio Observed / Unique Reflections (too) Low ..        49% Check
PLAT242_ALERT_2_C Low    'MainMol' Ueq as Compared to Neighbors of        C18 Check
PLAT341_ALERT_3_C Low Bond Precision on  C-C Bonds ...............    0.00717 Ang.
PLAT906_ALERT_3_C Large K Value in the Analysis of Variance ......     23.398 Check
PLAT906_ALERT_3_C Large K Value in the Analysis of Variance ......      4.623 Check
```

Alert level G

```
PLAT002_ALERT_2_G Number of Distance or Angle Restraints on AtSite          2 Note
PLAT172_ALERT_4_G The CIF-Embedded .res File Contains DFIX Records          1 Report
PLAT860_ALERT_3_G Number of Least-Squares Restraints ............           1 Note
PLAT910_ALERT_3_G Missing # of FCF Reflection(s) Below Theta(Min).          1 Note
                1  0  0,
PLAT912_ALERT_4_G Missing # of FCF Reflections Above STh/L=  0.600        819 Note
PLAT941_ALERT_3_G Average HKL Measurement Multiplicity ..........         2.2 Low
PLAT969_ALERT_5_G The 'Henn et al.' R-Factor-gap value ..........        2.70 Note
              Predicted wR2: Based on SigI**2  7.81 or SHELX Weight 21.54
PLAT978_ALERT_2_G Number C-C Bonds with Positive Residual Density.          0 Info
```

0 **ALERT level A** = Most likely a serious problem - resolve or explain
1 **ALERT level B** = A potentially serious problem, consider carefully
5 **ALERT level C** = Check. Ensure it is not caused by an omission or oversight
8 **ALERT level G** = General information/check it is not something unexpected

0 ALERT type 1 CIF construction/syntax error, inconsistent or missing data
4 ALERT type 2 Indicator that the structure model may be wrong or deficient
7 ALERT type 3 Indicator that the structure quality may be low
2 ALERT type 4 Improvement, methodology, query or suggestion
1 ALERT type 5 Informative message, check

It is advisable to attempt to resolve as many as possible of the alerts in all categories. Often the minor alerts point to easily fixed oversights, errors and omissions in your CIF or refinement strategy, so attention to these fine details can be worthwhile. In order to resolve some of the more serious problems it may be necessary to carry out additional measurements or structure refinements. However, the purpose of your study may justify the reported deviations and the more serious of these should normally be commented upon in the discussion or experimental section of a paper or in the "special_details" fields of the CIF. checkCIF was carefully designed to identify outliers and unusual parameters, but every test has its limitations and alerts that are not important in a particular case may appear. Conversely, the absence of alerts does not guarantee there are no aspects of the results needing attention. It is up to the individual to critically assess their own results and, if necessary, seek expert advice.

Publication of your CIF in IUCr journals

A basic structural check has been run on your CIF. These basic checks will be run on all CIFs submitted for publication in IUCr journals (*Acta Crystallographica*, *Journal of Applied Crystallography*, *Journal of Synchrotron Radiation*); however, if you intend to submit to *Acta Crystallographica Section C* or *E* or *IUCrData*, you should make sure that full publication checks are run on the final version of your CIF prior to submission.

Publication of your CIF in other journals

Please refer to the *Notes for Authors* of the relevant journal for any special instructions relating to CIF submission.

PLATON version of 06/01/2024; check.def file version of 05/01/2024

Datablock compound_6d - ellipsoid plot

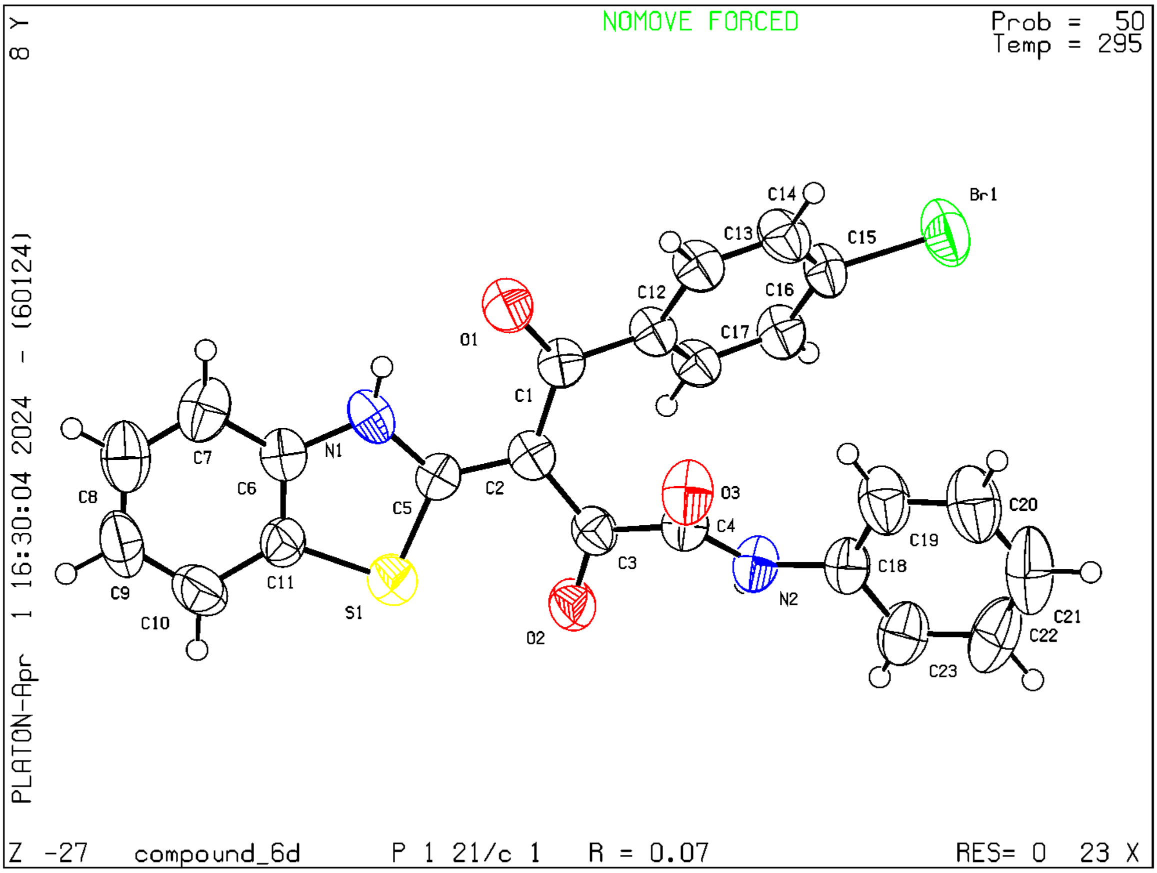

checkCIF/PLATON report

Structure factors have been supplied for datablock(s) compound_8a

THIS REPORT IS FOR GUIDANCE ONLY. IF USED AS PART OF A REVIEW PROCEDURE FOR PUBLICATION, IT SHOULD NOT REPLACE THE EXPERTISE OF AN EXPERIENCED CRYSTALLOGRAPHIC REFEREE.

No syntax errors found. CIF dictionary Interpreting this report

Datablock: compound_8a

```
Bond precision:   C-C = 0.0042 A          Wavelength=0.71073

Cell:             a=10.121(2)     b=18.482(5)        c=14.756(3)
                  alpha=90        beta=99.202(19)    gamma=90
Temperature:      295 K

                Calculated                  Reported
Volume          2724.7(11)                  2724.6(12)
Space group     P 21/n                      P 1 21/n 1
Hall group      -P 2yn                      -P 2yn
Moiety formula  C31 H31 N3 O4 S             C31 H31 N3 O4 S
Sum formula     C31 H31 N3 O4 S             C31 H31 N3 O4 S
Mr              541.65                      541.65
Dx,g cm-3       1.320                       1.320
Z               4                           4
Mu (mm-1)       0.161                       0.161
F000            1144.0                      1144.0
F000'           1144.97
h,k,lmax        14,25,20                    13,25,20
Nref            7571                        6501
Tmin,Tmax       0.958,0.971                 0.471,1.000
Tmin'           0.942

Correction method= # Reported T Limits: Tmin=0.471 Tmax=1.000
AbsCorr = MULTI-SCAN

Data completeness= 0.859          Theta(max)= 29.486

                                                      wR2(reflections)=
R(reflections)= 0.0590( 3346)                         0.1748( 6501)

S = 1.043                  Npar= 353
```

The following ALERTS were generated. Each ALERT has the format
test-name_ALERT_alert-type_alert-level.
Click on the hyperlinks for more details of the test.

Alert level C

```
PLAT230_ALERT_2_C Hirshfeld Test Diff for    C25      --C26      .        6.0 s.u.
PLAT340_ALERT_3_C Low Bond Precision on  C-C Bonds ...............    0.00419 Ang.
PLAT906_ALERT_3_C Large K Value in the Analysis of Variance ......      9.117 Check
PLAT906_ALERT_3_C Large K Value in the Analysis of Variance ......      2.211 Check
PLAT910_ALERT_3_C Missing # of FCF Reflection(s) Below Theta(Min).          7 Note
                1  1  0,   0  2  0,  -1  0  1,   1  0  1,  -1  1  1,   0  1  1,
                0  2  1,
```

Alert level G

```
PLAT912_ALERT_4_G Missing # of FCF Reflections Above STh/L=  0.600       1059 Note
PLAT941_ALERT_3_G Average HKL Measurement Multiplicity ..........        2.6 Low
PLAT969_ALERT_5_G The 'Henn et al.' R-Factor-gap value ..........       2.29 Note
              Predicted wR2: Based on SigI**2  7.64 or SHELX Weight 17.24
PLAT978_ALERT_2_G Number C-C Bonds with Positive Residual Density.          1 Info
```

0 **ALERT level A** = Most likely a serious problem - resolve or explain
0 **ALERT level B** = A potentially serious problem, consider carefully
5 **ALERT level C** = Check. Ensure it is not caused by an omission or oversight
4 **ALERT level G** = General information/check it is not something unexpected

0 ALERT type 1 CIF construction/syntax error, inconsistent or missing data
2 ALERT type 2 Indicator that the structure model may be wrong or deficient
5 ALERT type 3 Indicator that the structure quality may be low
1 ALERT type 4 Improvement, methodology, query or suggestion
1 ALERT type 5 Informative message, check

It is advisable to attempt to resolve as many as possible of the alerts in all categories. Often the minor alerts point to easily fixed oversights, errors and omissions in your CIF or refinement strategy, so attention to these fine details can be worthwhile. In order to resolve some of the more serious problems it may be necessary to carry out additional measurements or structure refinements. However, the purpose of your study may justify the reported deviations and the more serious of these should normally be commented upon in the discussion or experimental section of a paper or in the "special_details" fields of the CIF. checkCIF was carefully designed to identify outliers and unusual parameters, but every test has its limitations and alerts that are not important in a particular case may appear. Conversely, the absence of alerts does not guarantee there are no aspects of the results needing attention. It is up to the individual to critically assess their own results and, if necessary, seek expert advice.

Publication of your CIF in IUCr journals

A basic structural check has been run on your CIF. These basic checks will be run on all CIFs submitted for publication in IUCr journals (*Acta Crystallographica*, *Journal of Applied Crystallography*, *Journal of Synchrotron Radiation*); however, if you intend to submit to *Acta Crystallographica Section C* or *E* or *IUCrData*, you should make sure that full publication checks are run on the final version of your CIF prior to submission.

Publication of your CIF in other journals

Please refer to the *Notes for Authors* of the relevant journal for any special instructions relating to CIF submission.

PLATON version of 06/01/2024; check.def file version of 05/01/2024

Datablock compound_8a - ellipsoid plot

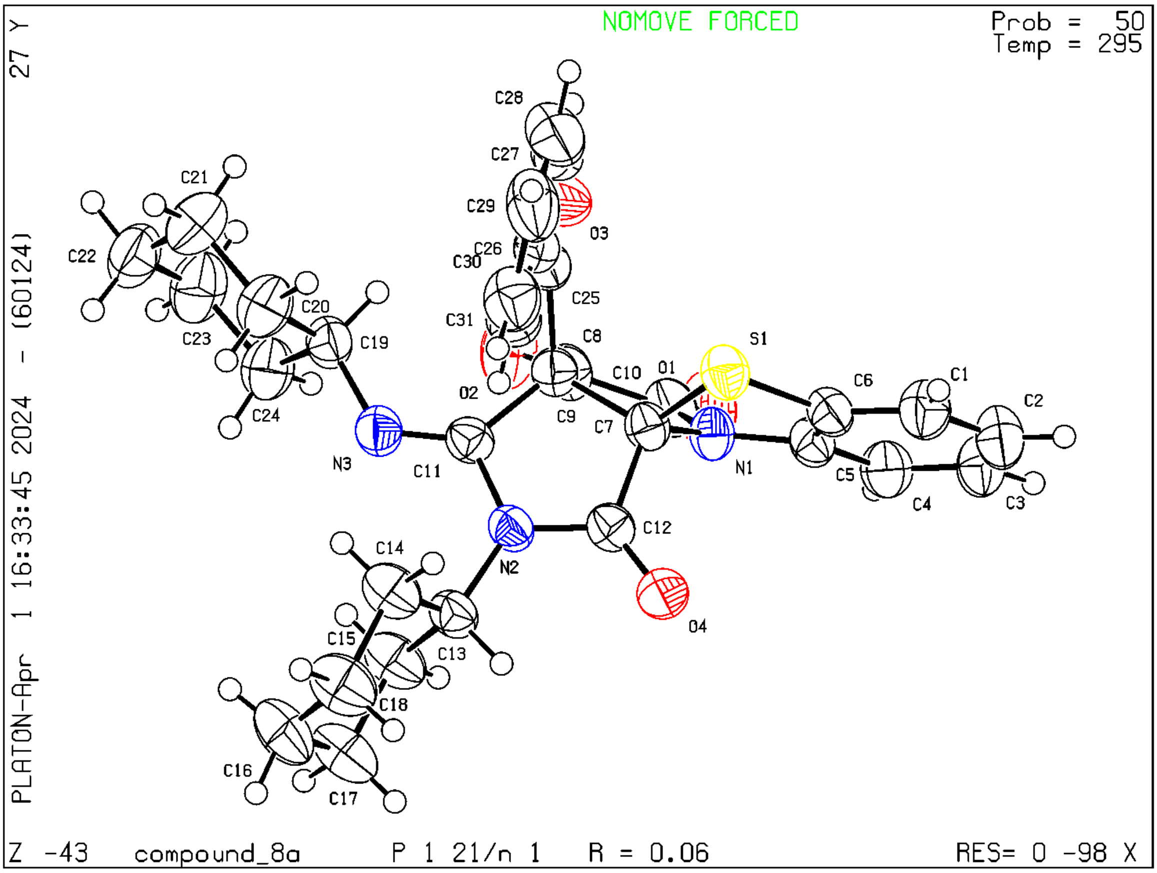

Supplement: Supplementary file 1 [file molecules-29-02089-s001.zip › X-ray/checkcif 3a, 4b, 6d, 8a.pdf]
