# Supplementary figures and images for: Reaction of Pyrrolobenzothiazines with Schiff Bases and Carbodiimides: Approach to Angular 6/5/5/5-Tetracyclic Spiroheterocycles
Source: Molecules. 2024 May 1;29(9):2089. doi: 10.3390/molecules29092089 (PMC11085407; doi:10.3390/molecules29092089)

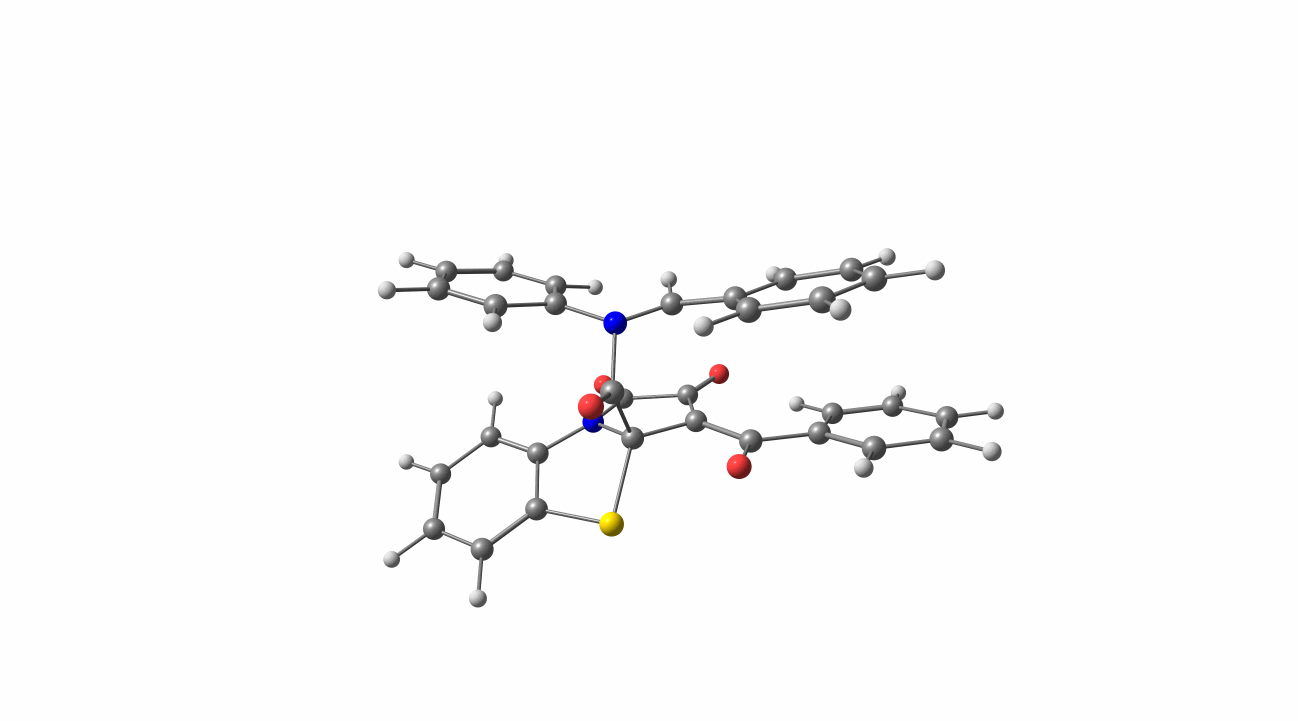

Supplement: Supplementary file 1 [file molecules-29-02089-s001.zip › xyz_files_for_optimized_model_structures/TS'.gif]

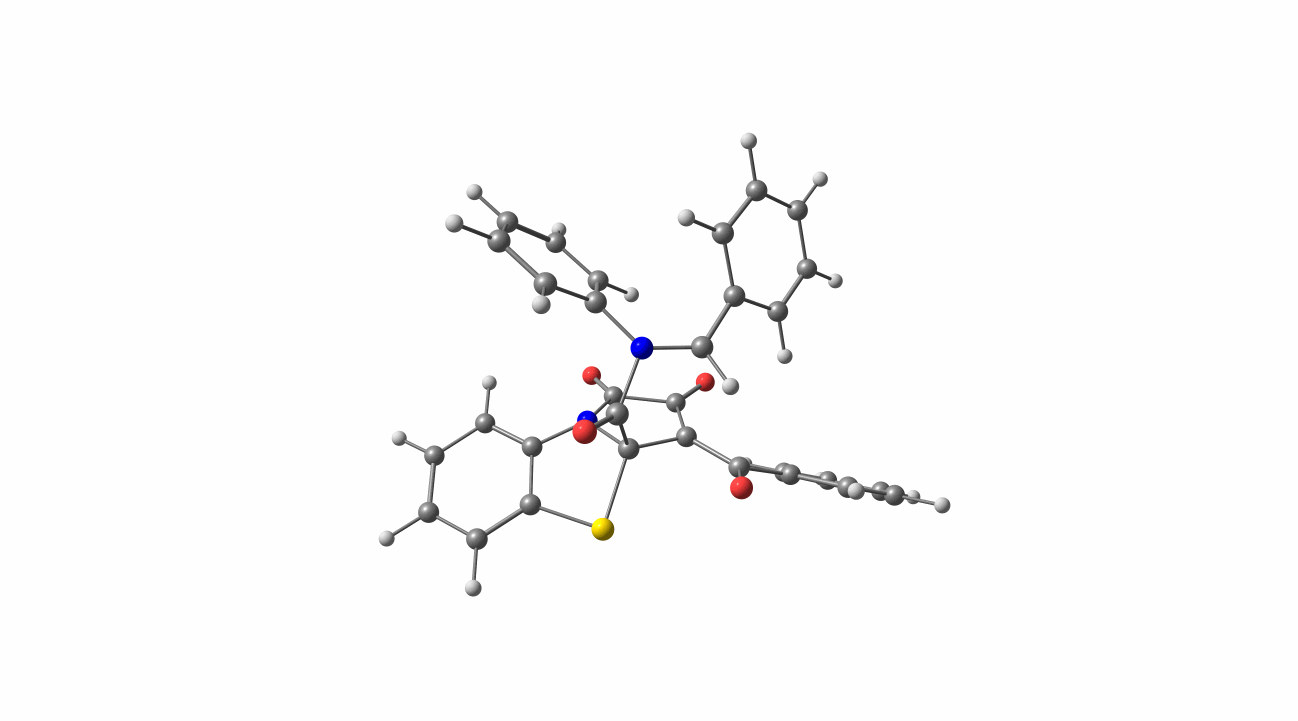

Supplement: Supplementary file 1 [file molecules-29-02089-s001.zip › xyz_files_for_optimized_model_structures/TS.gif]
